# Multiomics analysis reveals dermokine as a regulator of keratinocyte differentiation and adhesion

**DOI:** 10.1172/jci.insight.197838

**Published:** 2026-04-23

**Authors:** Vahap Canbay, Till Wüstemann, Weihua Tian, Tobias A. Beyer, Camilla Reiter Elbæk, Michael Stumpe, Gaetana Restivo, Chatpakorn Christiansen, Anabel Migenda Herranz, Susanne Mailand, Jürg Hafner, Rune Busk Damgaard, Steffen Goletz, Jörn Dengjel, Ulrich auf dem Keller, Chiara Francavilla

**Affiliations:** 1 Department for Biotechnology and Biomedicine, Technical University of Denmark, Lyngby, Denmark.; 2 Department of Biology, Swiss Federal Institute of Technology Zurich, Zurich, Switzerland.; 3 Cytosurge AG, Glattbrugg, Zurich, Switzerland.; 4 Division of Molecular and Cellular Function, Faculty of Biology, Medicine and Health, The University of Manchester, Manchester, United Kingdom.; 5 Department of Dermatology, University Hospital Zurich, Zurich, Switzerland.; 6 Department of Biology, University of Fribourg, Fribourg, Switzerland.

**Keywords:** Cell biology, Dermatology, Cell migration/adhesion, Proteomics, Signal transduction

## Abstract

Impaired adhesion and differentiation of keratinocytes is a hallmark of several skin diseases, but only some of the factors that regulate these processes have been identified. Here, we studied the role of isoform-rich dermokine — a wound- and tumor-regulated protein — in keratinocytes using a combination of multiomics and functional approaches. CRISPR/Cas9-induced knockout of dermokine isoforms in human keratinocytes inhibited differentiation of these cells in 3-dimensional organotypic skin cultures, which was confirmed by quantitative proteomics. In 2-dimensional monocultures, dermokine deficiency affected the proteome and phosphoproteome as revealed by mass spectrometry. We found reduced abundance of differentiation-specific proteins and increased phosphorylation of the cell adhesion protein p120 (catenin δ-1). The adhesive strength of dermokine-knockout keratinocytes was impaired, which was rescued by p120 knockdown or ROCK inhibition. Finally, we verified the correlation between decreased dermokine expression and increased p120 phosphorylation in human non-healing wounds. These results identify dermokine as a regulator of keratinocyte adhesion and differentiation, involving at least in part its effect on p120 phosphorylation and ROCK. Our data point to a function of dermokine in the pathogenesis of chronic wounds.

## Abstract 2

Our inter-disciplinary investigation reveals a novel molecular signature underlying skin keratinocytes differentiation and adhesion in vitro and in patient-derived samples

## Graphical abstract

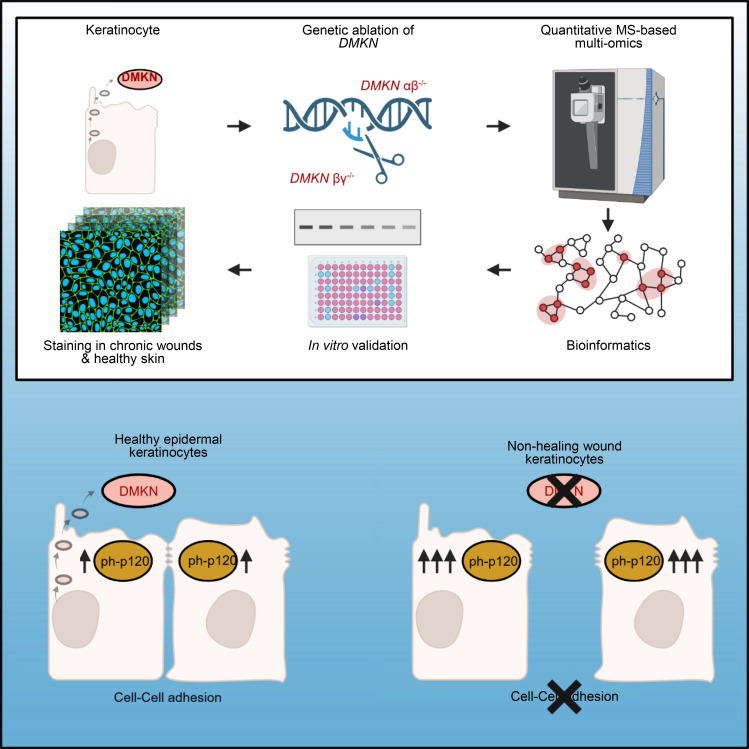

## Introduction

The skin is the largest organ of the human body and is composed of epidermis, dermis, and subcutis (1). Keratinocytes, the most prominent cell type of the epidermis, form a stratified epithelium through their gradual differentiation (2). The epidermis serves as a protective barrier against external threats, water loss, and mechanical strain (1). Therefore, impaired keratinocyte differentiation and epidermal stratification have severe consequences and are a hallmark of a plethora of skin diseases, such as psoriasis, epithelial skin cancers, and non-healing wounds (3). In squamous cell carcinomas (SCCs), keratinocytes lose the ability to differentiate into a stratified epithelium and undergo epithelial-mesenchymal transition mediated by the loss of cell-cell adhesion proteins (4). In psoriatic tissue, keratinocytes are exposed to high levels of inflammatory cytokines, causing excessive proliferation and aberrant differentiation (5). Further, in the context of non-healing wounds, keratinocytes at the wound edge remain in a vicious cycle of chronic inflammation, which also affects the wound bed. This prevents keratinocytes from migrating and re-epithelializing the wound (6–8). In summary, keratinocyte function must be tightly regulated to prevent such devastating diseases, and a deeper understanding of the molecular determinants underlying keratinocyte function, including the keratinocyte differentiation process, will open new avenues for the treatment of skin diseases.

Several molecules have been described as regulators of keratinocyte differentiation, including growth factors, calcium, and proteases (9, 10). Specifically, changes in the abundance, activity, and substrate availability as well as inhibition of protease activities have been associated with keratinocyte differentiation. Among the relevant players are kallikrein-related peptides (KLKs), the desquamation cascade, and the cathepsin-induced activation of transglutaminases (TGMs) (9). Studying the protease substrate repertoire is important for the understanding of keratinocyte differentiation (9). Among proteases with a function in keratinocytes, the wound- and cancer-associated matrix metalloproteinase 10 (MMP10), which is expressed in wound-edge keratinocytes of skin lesions, plays a crucial role in the cleavage of proteins regulating keratinocyte migration and adhesion (11, 12). We have previously identified dermokine, a member of the stratified epithelium–secreted peptide complex, as a substrate of MMP10 in basal keratinocytes (12). Dermokine is expressed by differentiating keratinocytes, absent in the basal layer, minimally present in the spinous layer, and most abundant in the granular layer (13). Even though a role of dermokine in modulating keratinocyte differentiation in mice has been suggested (14), the functions and mechanisms of action of dermokine in human keratinocytes remain unknown. This is relevant because of the multiple differences between murine and human epidermis (15, 16).

Dermokine is encoded by the *DMKN* gene, which encodes 3 major protein isoforms: dermokine-α, -β, and -γ (17). Alternatively spliced dermokine-β and dermokine-γ, which are generated from the same heterogeneous nuclear RNA, are secreted into the extracellular space and found in the granular layer of the epidermis, whereas dermokine-α RNA transcription is initiated 11,739 nucleotides further downstream and expressed. This isoform is expressed at similar levels throughout all epidermal layers (13, 18). In mice, knockout of dermokine-β and -γ caused neonatal skin hyperkeratosis, resembling psoriasis-like skin disorders (14). As mice deficient in all dermokine isoforms show a more severe phenotype, dermokine-α is likely to compensate for the loss of the other isoforms during keratinocyte differentiation (14). Here, to reveal the role of dermokine in keratinocyte differentiation in the human context, we generated human keratinocytes with CRISPR/Cas9-mediated knockout of different dermokine isoforms and characterized their behavior in 3-dimensional (3D) organotypic skin cultures (19, 20). These 3D cultures form epidermis-like structures, including the presence of differentiating keratinocytes, and have been widely used as an experimental model to study the mechanisms underlying skin diseases, including SCC (21, 22). To characterize the functions of DMKN in keratinocyte differentiation, these genetically engineered 3D cultures were analyzed by quantitative multiomics approaches, and the results were validated by functional assays in vitro and in vivo. We show that dermokine knockout affects the expression of epidermal proteins in 3D cultures, including p120, a known regulator of cell-cell adhesion (23). In summary, multiomics, functional analyses of keratinocyte models, and skin tissue staining revealed that dermokine regulates differentiation and cell-cell adhesion of keratinocytes.

## Results

### Dermokine is important for early keratinocyte differentiation in 3D skin equivalents.

**Figure 1:**
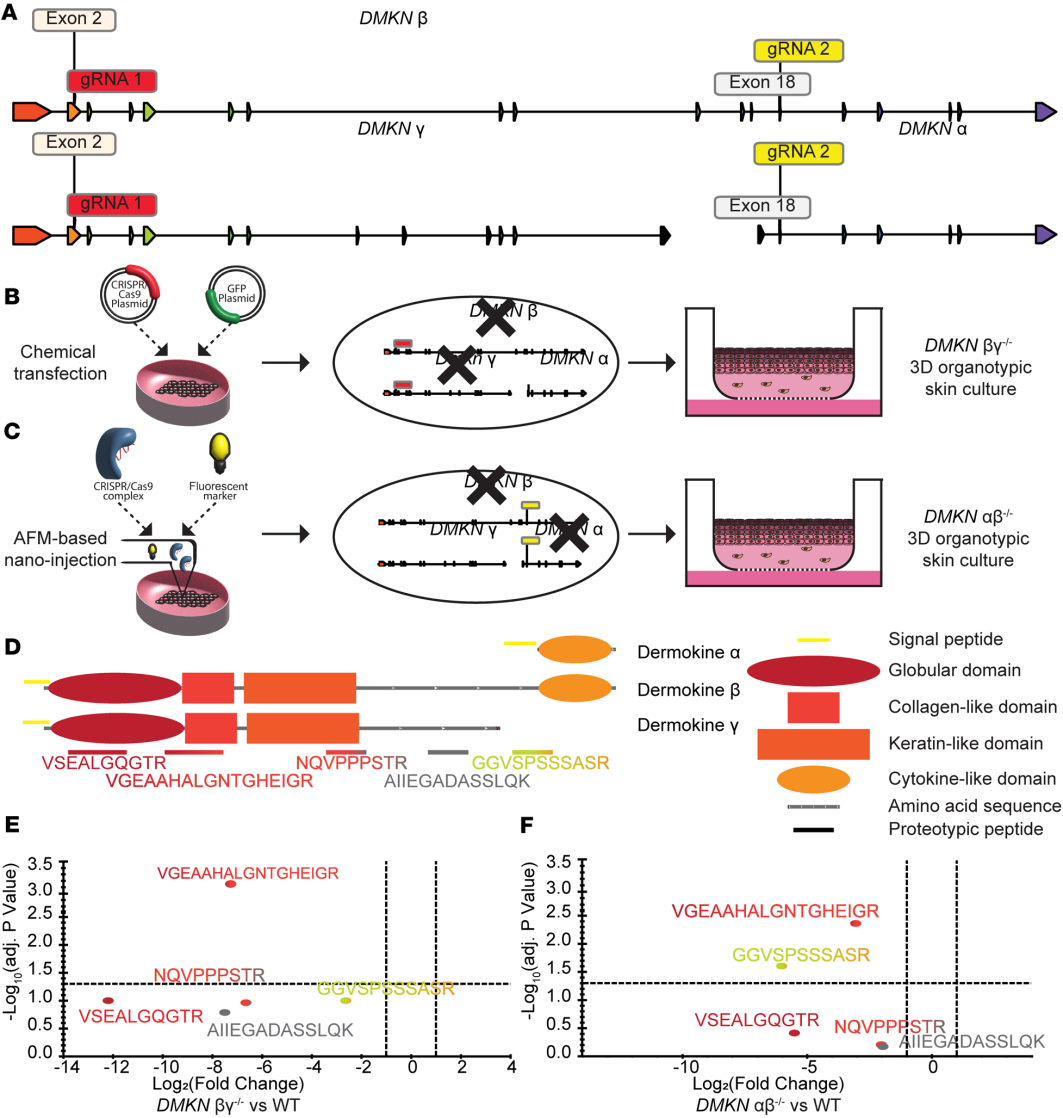
Targeted proteomics validates the knockout of dermokine in human keratinocytes. (**A**) Schematic of the genetic loci encoding the different dermokine isoforms and locations of guide RNAs (gRNA 1 and gRNA 2) targeting isoform-specific exons. (**B** and **C**) Methodologies used to delete dermokine in keratinocytes through either chemical transfection (**B**) or the FluidFM CRISPR approach (**C**). After transfection and clonal expansion, WT, *DMKN* αβ^–/–^, or *DMKN* βγ^–/–^ keratinocytes were used for the generation of 3D organotypic skin cultures. (**D**) Schematic of the protein domains of the dermokine isoforms and of the 5 isoform-specific and proteotypic peptides used for targeted proteomics analysis. (**E** and **F**) Proteotypic dermokine peptide abundances are log_2_-transformed fold changes of summed transition areas from *DMKN* βγ^–/–^ (**E**) or *DMKN* αβ^–/–^ (**F**) relative to WT keratinocytes analyzed by targeted proteomics. *N* = 3 different 3D organotypic skin cultures. Statistical significance was calculated using 2-sided Student’s *t* tests on log_2_-transformed intensities, with multiple-testing correction performed using the Benjamini-Hochberg false discovery rate.

**Figure 2:**
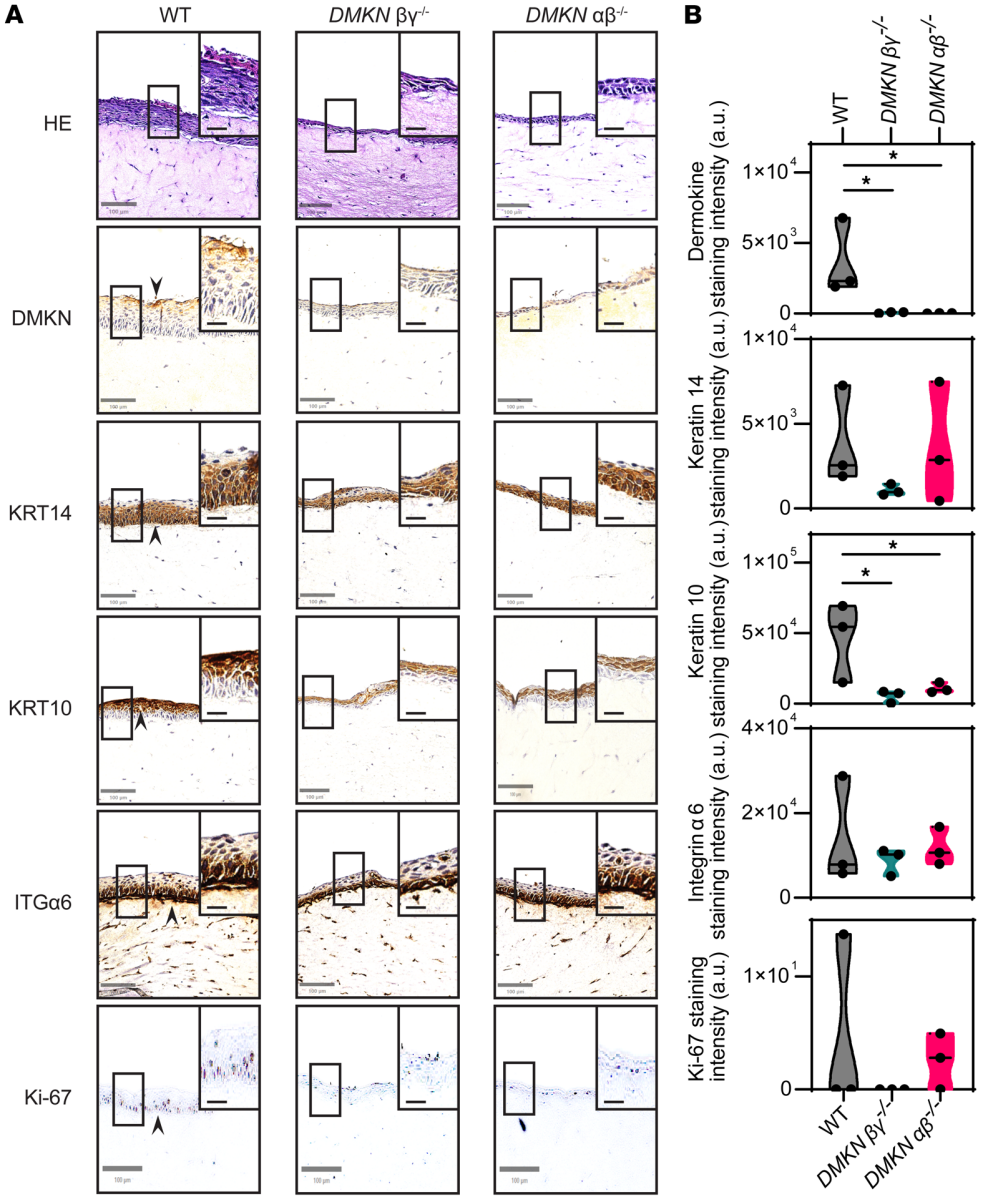
Staining of DMKN-KO and WT 3D organotypic skin cultures shows changes in epidermal marker proteins. (**A**) H&E and IHC staining with the indicated antibodies of WT and *DMKN* αβ^–/–^ or *DMKN* βγ^–/–^ keratinocyte 3D organotypic skin cultures. *N* = 3. Scale bars: 100 μm; and 25 μm in magnified images. (**B**) Truncated violin plots showing the semiquantitative IHC staining intensity with the indicated antibodies. *N* = 3 different 3D organotypic skin cultures. *P < 0.05 (1-way ANOVA and Fisher’s least significant difference test). a.u., arbitrary units.

To study a possible role of dermokine in keratinocyte differentiation (12), we edited the genomic sites coding for either dermokine-β/γ or -α/β (*DMKN* βγ or αβ) in human keratinocytes by two different CRISPR/Cas9 methods (19, 24) (Figure 1A). The immortalized but non-tumorigenic N/TERT keratinocyte cell line was used for this purpose, because it undergoes normal differentiation and stratification in 3D skin equivalents and allows the generation of clonally expanded lines (25). The first method, chemical transfection, which is based on fluorescence-activated cell sorting (FACS) of single-cell clones and clonal expansion, generated the *DMKN* βγ^–/–^ cell line (26) (Figure 1B). The second method, the FluidFM CRISPR approach, which was based on coinjection of CRISPR ribonucleoprotein (RNP) and green fluorescent protein (GFP) mRNA into keratinocytes, generated the *DMKN* αβ^–/–^ cell line (27) (Figure 1C). We confirmed the lack of expression of *DMKN* generated with either method by Sanger sequencing (28) (Supplemental Figure 1, A–D; supplemental material available online with this article; https://doi.org/10.1172/jci.insight.197838DS1). We then established 3D organotypic skin cultures by plating either WT or *DMKN* βγ^–/–^ or *DMKN* αβ^–/–^ keratinocytes on top of primary human fibroblasts incorporated in Matrigel, as previously described (19) (Figure 1, B and C). Under these conditions, keratinocytes form a multilayered, stratified epidermis, resembling human skin (19). We evaluated whether changing the calcium concentration in our experimental conditions from 1 mM to 5 mM would affect cell proliferation and differentiation by staining for keratin 14 (KRT14), a marker for non-differentiated keratinocytes, and for the early-differentiation marker KRT10 (Supplemental Figure 1E). Quantification of immunofluorescence stainings for KRT10 and KRT14 in varying calcium concentrations showed that the differentiation marker KRT10 did not change, while KRT14 levels decreased in response to elevated calcium (Supplemental Figure 1E). These results suggest that cells undergo the differentiation program in 3D organotypic skin culture, giving rise to a stratified, epidermis-like structure. To confirm changes in the expression of dermokine isoforms in this model system at the protein level, we could not use immunoblotting because of the lack of antibodies recognizing the specific isoforms. Instead, we used targeted proteomics and synthetic reference peptides (29, 30). We selected 5 proteotypic peptides spread across the entire dermokine sequence, including 2 near the N-terminus, 2 after the keratin-like domain, and 1 at the C-terminus (Figure 1D and Supplemental Table 1). As the C-terminal peptide GGVSPSSSASR is shared by both dermokine-β and -α (Figure 1D), deletion of the sole dermokine-β/γ isoform would still give a signal in the mass spectrometer because of the expression of the α isoform. Indeed, only a slight reduction in the peptide peak area was observed when only isoforms β and γ were genetically deleted (Figure 1E, Supplemental Figure 1F, and Supplemental Table 1). However, removing the dermokine-α and -β isoforms simultaneously resulted in a substantial decrease in the abundance of such a shared C-terminal peptide (Figure 1F, Supplemental Figure 1F, and Supplemental Table 1). Among the remaining 4 peptides, the N-terminal peptide VGEAAHALGNTGHEIGR showed the highest decrease in abundance upon depletion of both *DMKN* βγ and *DMKN* αβ in 3D organotypic skin cultures (Supplemental Figure 1, F–J, and Supplemental Table 1). Then, we analyzed WT and *DMKN*-KO 3D cultures by immunohistochemical (IHC) staining and showed the strong reduction of dermokine expression in both *DMKN* βγ^–/–^ and *DMKN* αβ^–/–^ compared with WT cultures (Figure 2). Hematoxylin and eosin as well as IHC staining revealed the lack of suprabasal layers beyond the spinous layer in the *DMKN*-KO compared with the WT samples (Figure 2). This observation was consistent with the known dermokine expression pattern in keratinocytes (13). Together, these data suggest that the expression of *DMKN* isoforms is indeed reduced at the protein level in our model system, which seems to affect keratinocyte differentiation.

Next, we evaluated whether the lack of dermokine affected the early-stage differentiation of keratinocytes by staining for KRT14 and KRT10 (1). The expression of KRT14 was comparable in WT and in both *DMKN*-KO cultures, whereas the expression of KRT10 decreased in the KOs compared with the WT samples (1) (Figure 2). These data suggest that dermokine is important for early keratinocyte differentiation. Expression of integrin α_6_ (ITGα6), a marker of basal keratinocytes (31), and expression of the proliferation marker Ki-67 were similar in WT and *DMKN*-KO keratinocytes, suggesting that hemidesmosome formation and proliferation do not change upon dermokine depletion (1) (Figure 2). Altogether, the IHC analysis suggests that the *DMKN* knockout reduces the amounts of differentiation-specific proteins.

### Knockout of dermokine variants alters the keratinocyte proteome in organotypic skin cultures.

**Figure 3:**
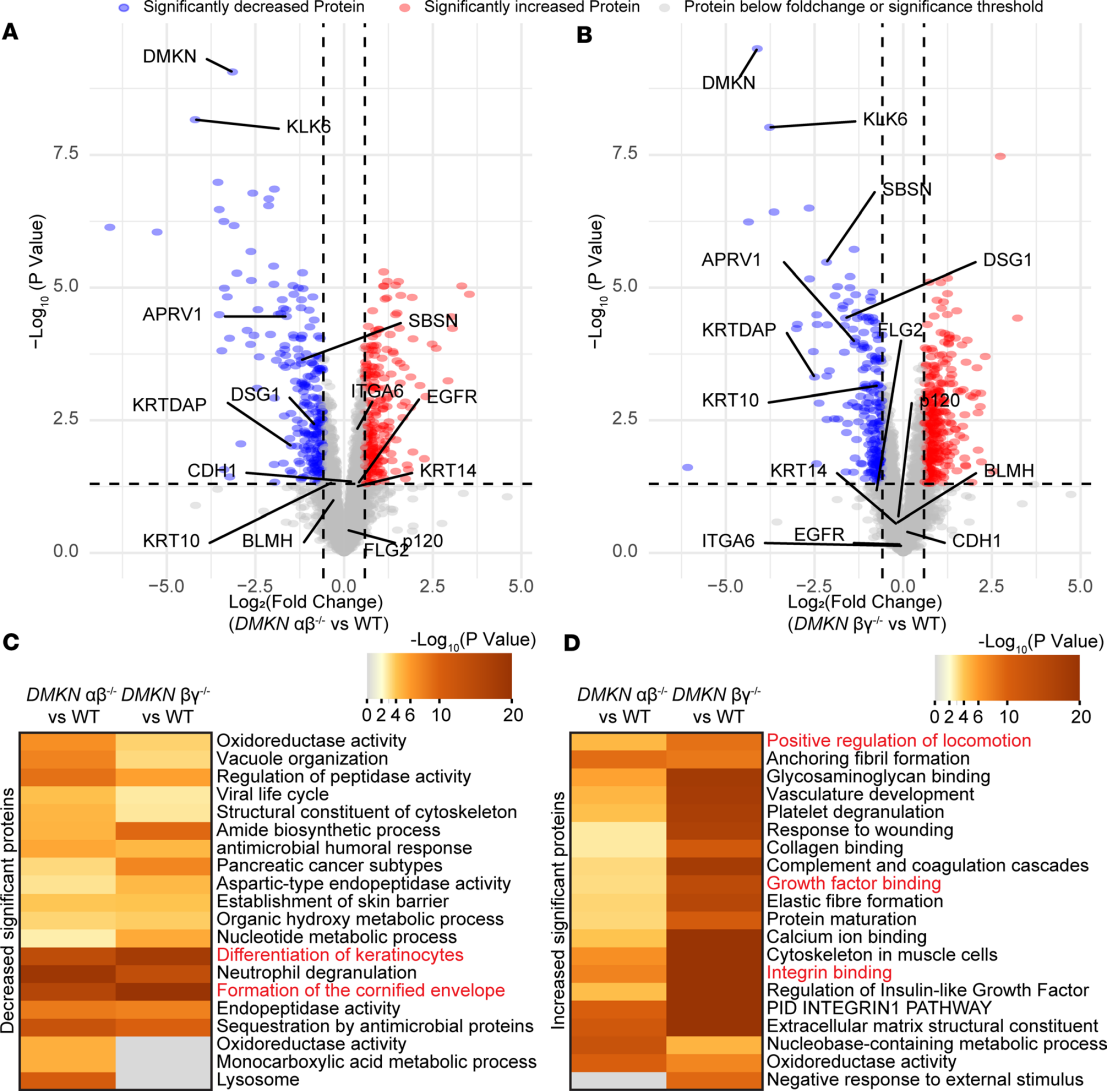
Proteomic analysis of dermokine-ablated 3D organotypic skin cultures shows phenotypic differences. (**A** and **B**) Volcano plots showing the differential protein abundance between *DMKN* αβ^–/–^ (**A**) or *DMKN* βγ^–/–^ (**B**) and WT keratinocytes in 3D organotypic skin cultures. Proteins characteristic of different epidermal strata are highlighted (1). Values are log_2_-transformed fold changes and –log_10_-transformed *P* values (2-sided moderated *t* test [empirical Bayes]) of 3 different (3D) organotypic skin cultures. (**C** and **D**) Gene Ontology analysis of significantly decreased (**C**) or increased (**D**) proteins from *DMKN* αβ^–/–^ (**A**) or *DMKN* βγ^–/–^ (**B**) relative to the WT proteome.

To characterize the phenotype of *DMKN*-KO samples in an unbiased manner, we analyzed changes in the proteome of the total 3D organotypic skin cultures grown from either WT or *DMKN*-KO keratinocytes via quantitative liquid chromatography/mass spectrometry–based (LC-MS–based) proteomics. In total, we quantified 3,753 proteins in WT, *DMKN* βγ^–/–^, and *DMKN* αβ^–/–^ 3D cultures with a coefficient of variation (CV) of less than 15% between the replicates (Supplemental Figure 2A and Supplemental Table 2). The Pearson’s correlation analysis of WT, *DMKN* βγ^–/–^, and *DMKN* αβ^–/–^ 3D cultures revealed high correlation among replicates for each experimental condition (Supplemental Figure 2B), in line with previous publications (32, 33). Finally, principal component analysis (PCA) demonstrated clear differences in the proteome of cultures with the 3 different genotypes (Supplemental Figure 2C). Hierarchical clustering identified clusters including proteins that are more abundant in the WT (cluster 2), in both the KO (cluster 4), or only in one of the two KO samples (clusters 1 and 3) (Supplemental Figure 2D), suggesting individual but also overlapping functions of the two dermokine isoforms in keratinocytes. Gene Ontology (GO) term enrichment analysis showed that proteins in cluster 2 were enriched in terms like “formation of the cornified envelope” and proteins in cluster 4 were associated with signaling pathways (Supplemental Figure 2E). The differential abundance analysis, visualized by volcano plot, revealed 312 and 231 significantly more abundant proteins in the *DMKN* βγ^–/–^ and *DMKN* αβ^–/–^ 3D cultures, respectively, when compared with WT (Figure 3A and Supplemental Table 2). We also identified 217 and 230 significantly less abundant proteins in the respective *DMKN* βγ^–/–^ and *DMKN* αβ^–/–^ keratinocytes in comparison with the WT (Figure 3B and Supplemental Table 2). The protein abundance of dermokine and KRT10 was reduced in both *DMKN*-KO 3D cultures, confirming the results of the IHC staining (Figure 2 and Figure 3, A and B). KRT14 and ITGα6 showed no differential abundance in the *DMKN* βγ^–/–^ clone, as seen in the IHC analysis, but were slightly increased in the *DMKN* αβ^–/–^ clone (Figure 2 and Figure 3, A and B). Along with dermokine, the abundance of suprabasin (SBSN) and keratinocyte differentiation–associated protein (KRTDAP), which are all members of the stratified epithelium–secreted peptides complex (SSC) expressed in the suprabasal layers of the stratified epithelium (34), was also reduced (Figure 3, A and B). GO enrichment analysis of significantly less abundant proteins from both the *DMKN* KOs compared with WT clones showed the enrichment of pathways such as “keratinocyte differentiation” and “formation of the cornified envelope” (Figure 3C), whereas the GO enrichment analysis of significantly more abundant proteins showed the enrichment of “integrin binding,” “positive regulation of locomotion,” and “growth factor binding” pathways (Figure 3D).

In conclusion, dermokine regulates the abundance of proteins of the suprabasal layers of the epidermis in human skin equivalents.

### Phosphoproteomics analysis reveals that dermokine regulates cell-cell adhesion proteins.

To uncover how loss of dermokine may affect keratinocyte differentiation, we studied changes in cellular signaling by deep quantitative proteomics and phosphoproteomics analyses of WT, *DMKN* αβ^–/–^, and *DMKN* βγ^–/–^ keratinocytes in monocultures. In addition, we tested whether treatment of the KO samples with recombinant dermokine-β (35) rescues the alterations in the proteome and phosphoproteome.

We first focused on the proteome and found 5,118 unique quantified proteins (Supplemental Table 3) with the median CV of the protein groups being below 20% for all samples and the correlation analysis showing high correlation between replicates, but clear differences between the different genotypes in the PCA analysis (Supplemental Figure 3, B–D). The *DMKN* αβ^–/–^ or *DMKN* βγ^–/–^ proteomes differed from the WT proteome, as shown by hierarchical clustering (Supplemental Figure 4A), and this difference was not rescued by treatment with recombinant dermokine. When we subjected the significantly differentially abundant proteins in the different samples to GO enrichment analysis, we found several enriched GO terms, including the term “cadherin binding”, reciprocally enriched in 2 conditions: when comparing the most abundant proteins from non-treated *DMKN* αβ^–/–^ and *DMKN* βγ^–/–^ cells with those from WT keratinocytes (Supplemental Figure 4B) and when comparing the significantly less abundant proteins from the dermokine-treated *DMKN* αβ^–/–^ and *DMKN* βγ^–/–^ cell lines with those from WT keratinocytes (Supplemental Figure 4C). These 2D results confirm that dermokine knockout changes the keratinocyte proteome (Figure 3).

**Figure 4:**
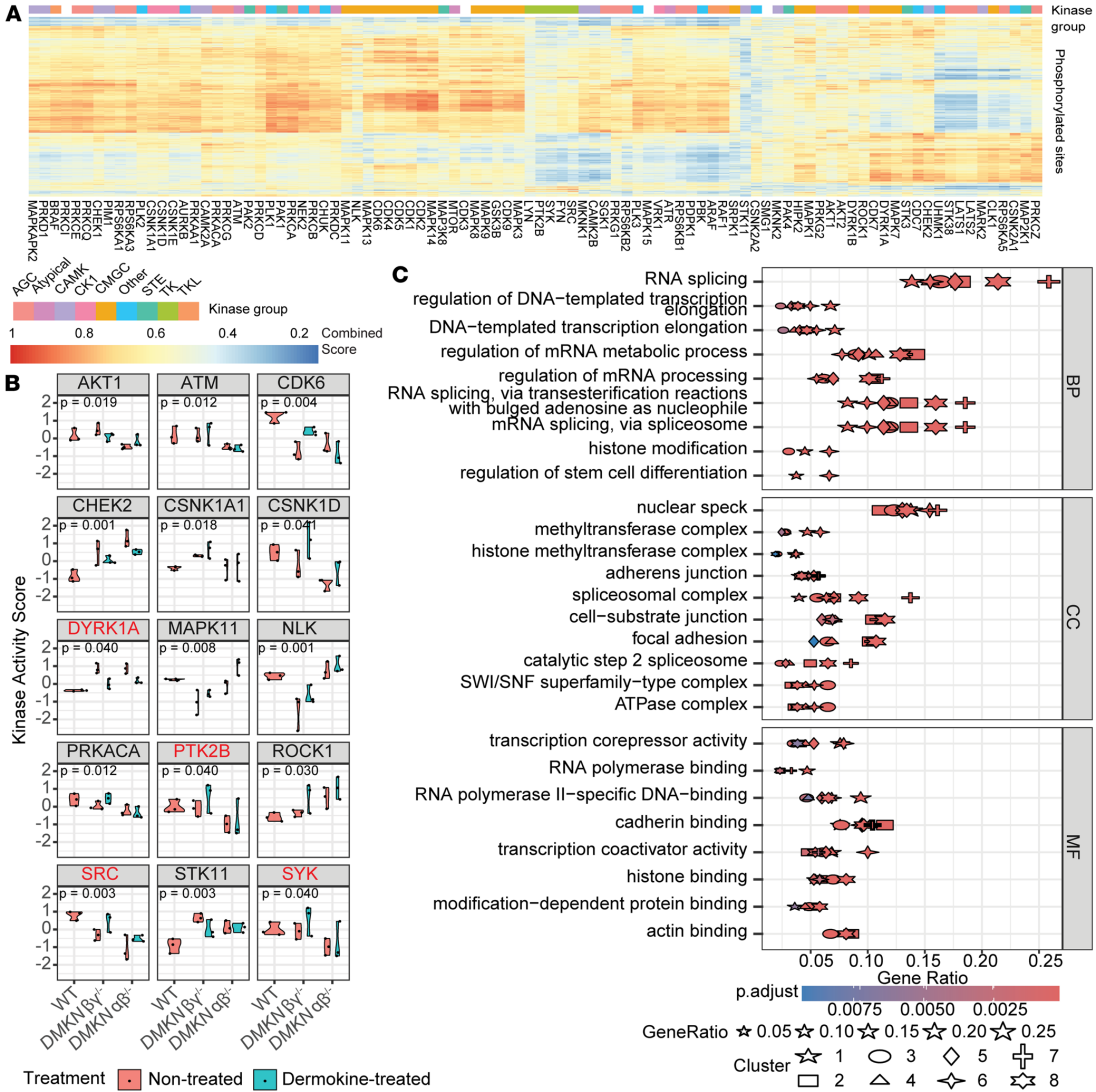
Absence of dermokine results in phosphorylation changes in adhesion proteins. (**A**) The kinase-substrate prediction approach (39) matched quantified phosphorylated sites in the WT and the two KO samples with the 94 identified putative kinases (Supplemental Table 5). The heatmap shows the combined score of the in vivo or in vitro kinases and sequence recognition motifs (39). The vertical axis shows substrates, and the horizontal axis represents kinases. Families of kinases are color-coded on top of the graph. (**B**) The kinase activity score for *DMKN* αβ^–/–^, *DMKN* βγ^–/–^, and WT conditions, treated with recombinant dermokine or vehicle, is shown for 15 significantly regulated kinases out of the 94 identified kinases. Values are kinase activity scores from *N* = 3 technical replicates from the different genotypes and treatments. One-way ANOVA test determined changes among all genotypes. (**C**) Gene Ontology analysis from proteins whose phosphorylated sites were annotated to kinases identified in clusters shown in Supplemental Figure 6. BP, biological process; CC, cellular component; MF, molecular function.

**Figure 5:**
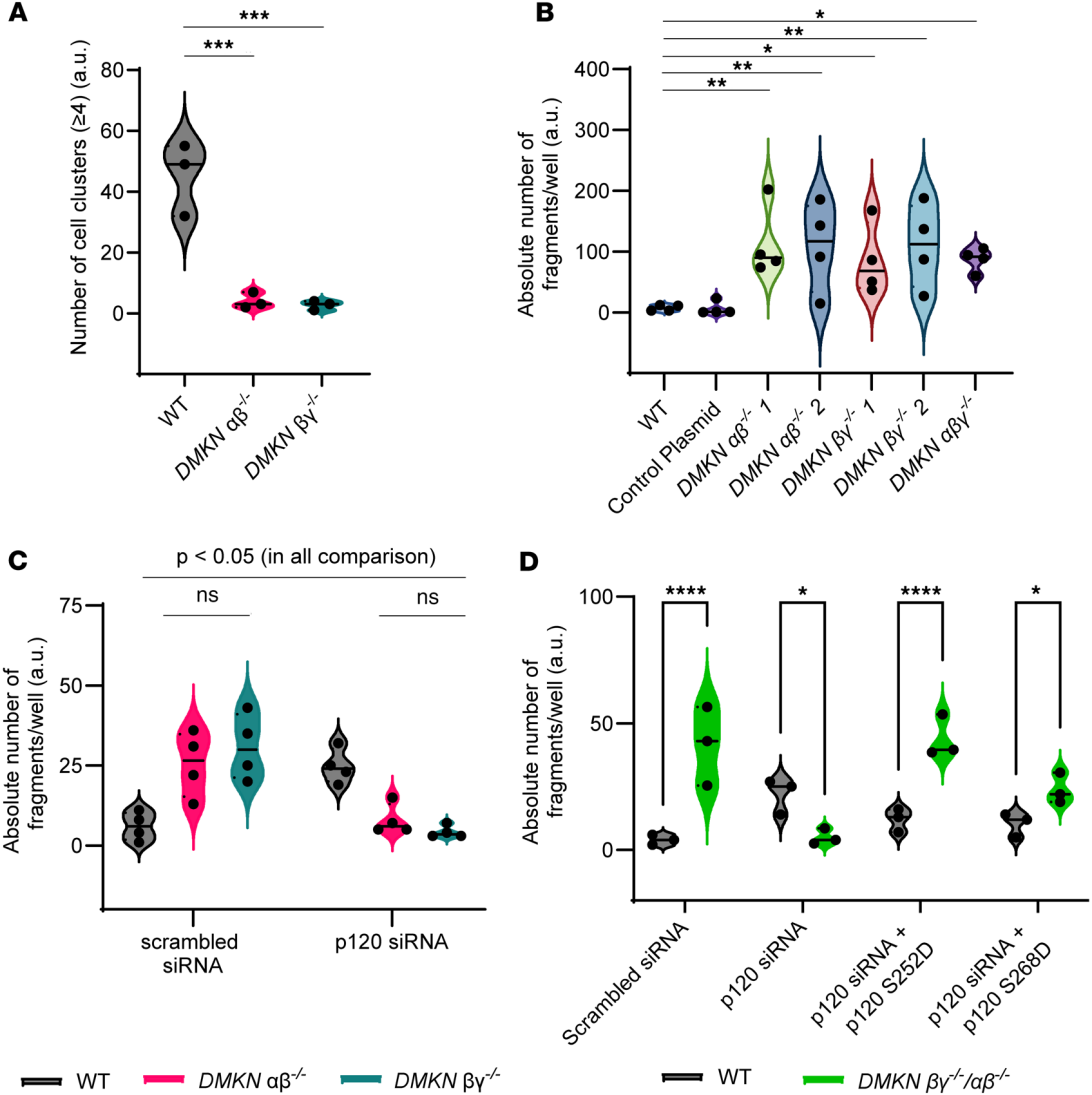
Cell-cell adhesion decreases in dermokine*-*depleted keratinocytes. (**A**) Quantification of 4 or more cell clusters from the cell-cell adhesion assay using *DMKN* αβ^–/–^, *DMKN* βγ^–/–^, and WT keratinocytes. Cell clusters (≥4) were counted, and each replicate was visualized as a dot in the violin plot. *N* = 3 biological replicates. ***P < 0.001 (1-way ANOVA and Fisher’s least significant difference test). (**B**) Quantification of dispase dissociation assay data using independent clones of human *DMKN* αβ^–/–^, *DMKN* βγ^–/–^, *DMKN* αβγ^–/–^, and WT keratinocytes transfected for 72 hours with p120 or scrambled siRNA. Values are normalized to total fragment size. *N* = 4 biological replicates. *P < 0.05, **P < 0.01 (1-way ANOVA and Fisher’s least significant difference test). (**C**) Quantification of dispase dissociation assay data using *DMKN* αβ^–/–^, *DMKN* βγ^–/–^, and WT keratinocytes transfected for 72 hours with p120 (1, 2, and 3) or scrambled siRNA. *N* = 4 biological replicates. All conditions are significant (*P* < 0.05) unless indicated otherwise (ns: *P* > 0.05) (2-way ANOVA and Fisher’s least significant difference test). (**D**) Quantification of dispase dissociation assay data of *DMKN* αβ^–/–^, *DMKN* βγ^–/–^, and WT keratinocytes transfected for 48 hours with p120 or scrambled siRNA followed by transfection with expression vectors encoding p120 mutants with a siRNA-resistant mutation and either S252D or S268D mutations. *N* = 3 biological replicates. *P < 0.05, ****P < 0.0001 (2-way ANOVA and Fisher’s least significant difference test).

Next, we analyzed changes in the phosphoproteome of non-treated and dermokine-treated *DMKN* βγ^–/–^ and *DMKN* αβ^–/–^ cell lines compared with WT keratinocytes (Supplemental Figure 3A and Supplemental Table 4). We enriched phosphorylated peptides using automated iron(III)–nitrilotriacetic acid [Fe(III)-NTA] and used a modified high-resolution MS–data-independent acquisition (HRMS1-DIA) strategy covering a 400 to 1,400 *m*/*z* mass range (36–38) (Supplemental Figure 5A). In total, we identified 11,778 phosphorylation sites after filtering for localization site probability (≥0.75) and 4,453 fully quantifiable phosphorylated sites across all conditions on 2,695 proteins (Supplemental Figure 5B and Supplemental Table 4). All replicates showed high correlation scores (Supplemental Figure 5C), and we found 10,027 singly, 1,367 doubly, and 384 multiply phosphorylated sites (Supplemental Figure 5D) and 10,315, 1,164, and 299 phosphorylated serine, threonine, and tyrosine amino acids, respectively (Supplemental Figure 5E). Overall, the quality of the phosphoproteomics datasets was consistent with previous publications (38). We observed significant differences between the phosphoproteome of *DMKN* βγ^–/–^ and *DMKN* αβ^–/–^ cell lines compared with the WT cell phosphoproteome (4,453 phosphorylated sites) by hierarchical clustering (Supplemental Figure 5F). The phosphoproteome of dermokine-treated *DMKN* βγ^–/–^ and *DMKN* αβ^–/–^ keratinocytes was similar to the phosphoproteome of WT cells, suggesting a considerable reversal of the abnormal phosphorylation pattern of the KO keratinocytes upon treatment with recombinant dermokine (Supplemental Figure 5F). To study how dermokine affects cellular signaling, we applied a global kinase-substrate prediction approach (39), which reveals the likelihood of a kinase modifying a phosphorylated site by linking kinase recognition sequence motifs and known in vivo and in vitro phosphorylation profiles (39) to the 4,453 phosphorylated sites identified in the WT and the KO samples. This approach identified 94 putative kinases (Supplemental Table 5) whose known downstream phosphorylated sites were visualized in a heatmap (Figure 4A). Among the 94 kinases and based on the kinase activity score, we identified 30 kinases with a high change and 15 kinases that showed significantly high changes between the endogenous WT and all the *DMKN* βγ^–/–^ and *DMKN* αβ^–/–^ samples (Figure 4B and Supplemental Table 5). Four of the latter kinases were cytoplasmic tyrosine kinases (DYRK1A, PTK2B, SRC, and SYK), of which both SRC and SYK showed decreased activity scores in the *DMKN* βγ^–/–^ and *DMKN* αβ^–/–^ keratinocytes compared with WT (Figure 4B). Similarly, our data showed significantly higher activity scores of ROCK1, a key regulator of the cytoskeleton (40, 41), in KO keratinocytes relative to WT cells (Figure 4B). Next, we used the kinase-substrate annotations generated by the global kinase-substrate prediction approach to cluster substrates sharing similar kinase profiles and regulation (39), and we identified 8 clusters (Supplemental Figure 6 and Supplemental Table 5). Interestingly, each cluster had a different distribution of phosphorylated serine, threonine, and tyrosine residues, ranging from clusters 2, 3, 4, and 6 not containing any phosphorylated tyrosines, to cluster 8 containing only phosphorylated serines, and clusters 5 and 7 mainly containing phosphorylated tyrosine and threonine sites, with cluster 7 being associated with PTK2B, ROCK1, SRC, and SYK activity (Supplemental Figure 6). GO enrichment analysis of the 8 clusters revealed enrichment of terms related to RNA regulation in all clusters, specifically in cluster 7, which, together with cluster 2, also contained several proteins associated with “adherens junctions” and “cadherin binding” (Figure 4C). This finding is in line with SRC, a known player in cell-cell adhesion (42), being the most likely kinase regulating the proteins belonging to cluster 7 (Supplemental Figure 6) and with “cadherin binding” being the most strongly dysregulated pathway in the *DMKN* βγ^–/–^ and *DMKN* αβ^–/–^ keratinocytes compared with WT (Supplemental Figure 4). To identify potential dermokine-regulated phosphorylated sites, we counted the number of sites on each phosphorylated protein belonging to the pathways associated with “cadherin binding” and “adherens junctions” associated with clusters 2, 4, 5, and 7. We found several phosphorylated sites (between 8 and 12) on proteins associated with cell-cell adhesion, including on TNKS1BP1 (12), TJP1 (11), SCRIB (10), TJP2 (10), CTNNB1 (9), AFDN (8), and CTNND1 (or p120) (8) (Supplemental Figure 6 and Supplemental Table 5). Among these proteins, p120 was phosphorylated on S252, S268, S288, and T916, all sites playing crucial roles in modulating cell-cell adhesion complexes and cytoskeletal dynamics (23, 43, 44). To confirm that the regulated phosphorylated sites identified on p120 as well as other proteins were bona fide phosphorylated sites due to changes in kinase activity and independent of alterations in protein abundance (45), we normalized the phosphoproteome on the proteome (Supplemental Figure 7A). We found that the normalized phosphoproteome of WT differed from that of *DMKN* βγ^–/–^ and *DMKN* αβ^–/–^ keratinocytes. When the *DMKN* βγ^–/–^ and *DMKN* αβ^–/–^ keratinocytes were treated with recombinant dermokine, their phosphoproteome became similar to the phosphoproteome of WT keratinocytes (Supplemental Figure 7A). This finding is consistent with the result observed before normalization (Figure 5, A and B). The phosphorylation pattern on p120 also remained unchanged after normalization (Supplemental Table 4). We validated the phosphorylation of p120 on two of the identified phosphorylated sites, serine 252 (S252) and serine 268 (S268), for which antibodies and fully annotated MS/MS spectra were available (Supplemental Figure 7, B and C). Immunoblot analysis of lysates from *DMKN* βγ^–/–^, *DMKN* αβ^–/–^, and WT keratinocytes showed that the phosphorylation of both S252 and S268 on p120 increased in the absence of dermokine (Supplemental Figure 7D). Furthermore, we observed that p120 phosphorylation was similar to WT when recombinant dermokine was added to *DMKN* βγ^–/–^, *DMKN* αβ^–/–^, and WT keratinocytes (Supplemental Figure 7E). In summary, the phosphoproteomics analyses of dermokine KO samples identified differentially phosphorylated proteins, including p120.

### Dermokine regulates cell-cell adhesion in keratinocytes.

As p120 is known to regulate cell-cell adhesion (44) and “cell-cell adhesion” was also one of the dysregulated GO terms in the proteomics and phosphoproteomics analysis of the KO samples (Figure 4C, Supplemental Figure 4, and Supplemental Table 5), we next tested whether dermokine regulates cell-cell adhesion of keratinocytes using 2 different cell-cell adhesion assays (46, 47). An EDTA-based cell-cell adhesion assay showed that dermokine-ablated keratinocytes had significantly fewer cell clusters containing more than 4 cells compared with WT keratinocytes after EDTA treatment (Figure 5A and Supplemental Figure 8A). This finding suggests impaired cell-cell adhesion in dermokine-depleted cells. To further test this possibility, we performed a dispase dissociation assay using independent *DMKN* βγ^–/–^, *DMKN* αβ^–/–^, and *DMKN* αβγ^–/–^ clones (47), and found an increase in the fragmentation of all the KO cells after dispase treatment (Figure 5B). These results confirm the impaired cell-cell adhesion in the KO cells, regardless of dermokine isoforms and clones. As we did not observe a change in cadherin-1 (CDH1) abundance by proteomic analysis of 2D cultures and IHC staining of WT, *DMKN* βγ^–/–^, and *DMKN* αβ^–/–^ 3D organotypic skin cultures (Figure 3, A and B, and Supplemental Figure 8, B and C), we tested whether p120 may be involved in the dermokine-mediated effect on cell-cell adhesion in keratinocytes. We performed the same cell-cell adhesion assays in keratinocytes depleted of p120 by small interfering RNA (siRNA). The efficient knockdown was confirmed by immunoblotting (Supplemental Figure 8D). As previously shown (44), knockdown of p120 significantly decreased cell-cell adhesion in WT cells (Figure 5C and Supplemental Figure 8, E–G). Surprisingly, however, depletion of p120 in *DMKN* αβ^–/–^ and *DMKN* βγ^–/–^ keratinocytes decreased fragmentation in the dispase dissociation assay and increased the number of cell clusters containing more than 4 cells in the EDTA-based cell-cell adhesion assay, reaching values similar to those seen with WT cells (Figure 5C and Supplemental Figure 8, E–H). These findings suggest that the increased phosphorylation of p120 observed in KO cells (Supplemental Figure 7, D and E) may have a negative effect on the adhesive properties of p120 downstream of dermokine, confirming observations in other model systems (23, 48, 49). To test this idea, we generated siRNA-resistant mutants of p120 in which aspartic acid (D) was substituted for S252 and S268 (S252D, S268D) to mimic constitutive phosphorylation (Supplemental Figure 8I) and used them in the dispase dissociation assay. We confirmed that p120 depletion reduced fragmentation in KO keratinocytes (Figure 5, C and D). Most importantly, upon expression of the 2 phosphomimetic p120 mutants in p120-knockdown cells, the fragmentation in KO keratinocytes returned to the level seen in unperturbed KO cells (Figure 5D). These data suggest that dermokine modulates cell-cell adhesion, at least in part, through p120 phosphorylation. Together, our data demonstrate that dermokine regulates cell-cell adhesion in keratinocytes, possibly by affecting phosphorylation of p120.

### Dermokine is a regulator of ROCK signaling.

**Figure 6:**
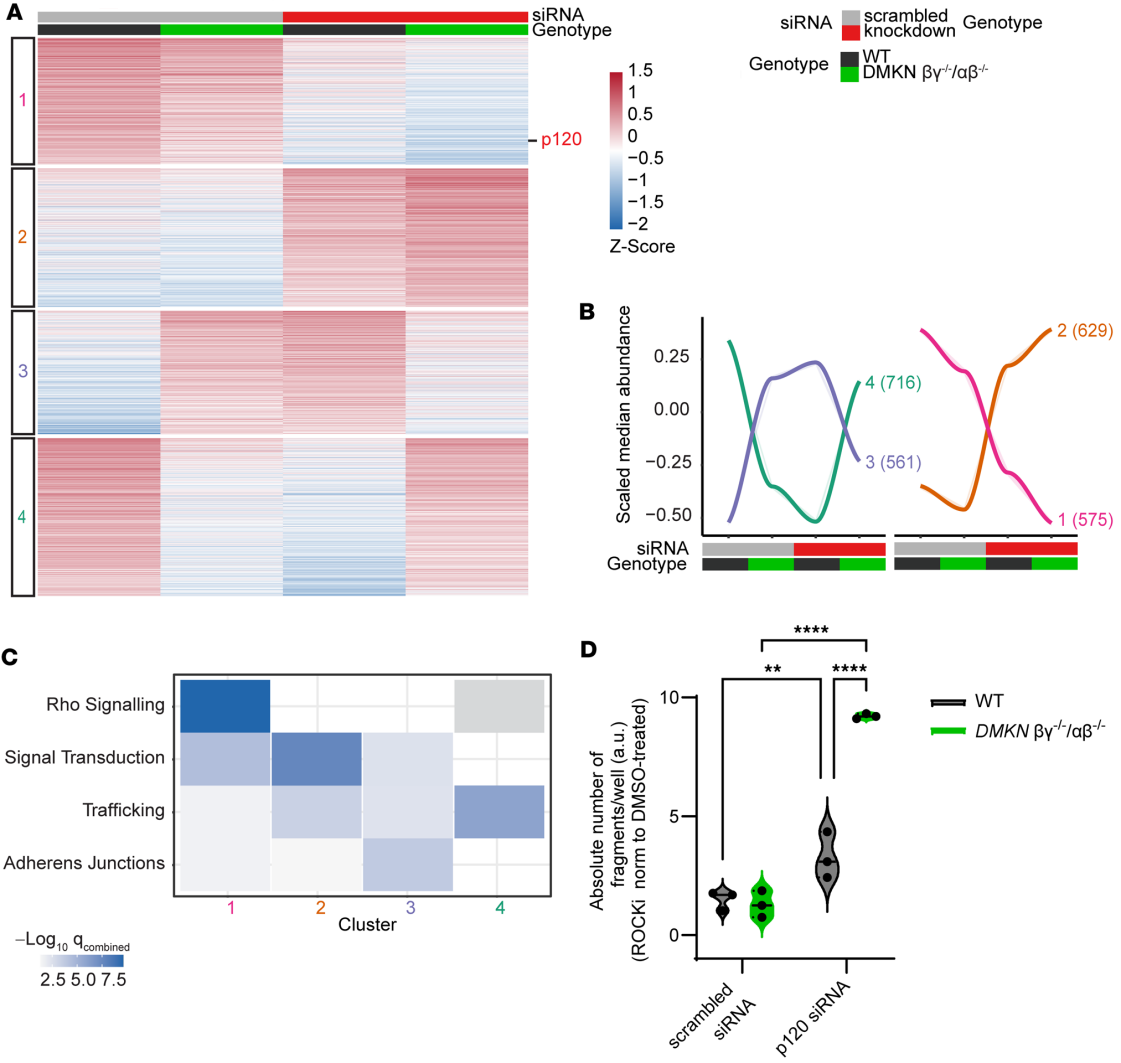
p120 perturbation and ROCK1 inhibition in keratinocytes regulate cell-cell adhesion. (**A**) Heatmap of the *Z*-scored differentially expressed proteins in the indicated genetic background show 4 distinct clusters. Genotypes and conditions are color-coded on top of the graph. (**B**) Line plot of scaled abundances of all proteins within clusters from **A**. Numbers indicate cluster number and total proteins for each cluster (in parentheses). (**C**) Pathway enrichment analysis of proteins in clusters 1, 2, 3, and 4. The color scale [–log_10_(*q*_combined_)] represents the aggregated and multiple-testing-corrected significance of the module in each cluster. Shown are overhead terms, and all terms can be found in Supplemental Table 8. (**D**) Quantification of dispase dissociation assay using *DMKN* αβ^–/–^, *DMKN* βγ^–/–^, and WT keratinocytes transfected for 48 hours with p120 or scrambled siRNA and treated with ROCK inhibitor (ROCKi) for 24 hours. Data are normalized to DMSO-treated values. *N* = 3 biological replicates. **P < 0.01, ****P < 0.0001 (2-way ANOVA and Fisher’s least significant difference test).

To gain insight into the molecular mechanisms underlying the dermokine-p120 interplay, we transfected WT and dermokine-KO keratinocytes with p120 or control siRNA and analyzed changes in the proteome. Using HRMS1-DIA, we identified 101,288 precursors, 78,704 peptides, and 7,394 proteins across all conditions, with high Pearson’s correlations between biological replicates (Supplemental Figure 9, A and B, and Supplemental Table 6). Hierarchical clustering of differentially abundant proteins revealed 4 clusters with distinct abundance patterns (Figure 6A). For instance, cluster 1 contained proteins whose abundance decreased upon p120 perturbation, including p120 itself and proteins regulating cell adhesion, such as α- and β-catenin and cadherin (Figure 6, A and B, and Supplemental Table 7). Interestingly, cluster 4 included proteins whose abundance followed the pattern observed in the dispase and wound closure assays (Figure 5 and Figure 6, A and B). Pathway enrichment analysis across clusters highlighted overhead terms associated with RhoA signaling, signal transduction, transport, and adherens junctions (Figure 6C, Supplemental Figure 9C, and Supplemental Table 8). Specifically, cluster 4 was enriched for RhoA-associated proteins, like cluster 1, suggesting that p120-perturbed KO cells and WT unperturbed keratinocytes shared similar levels of proteins associated with Rho signaling (Figure 6C, Supplemental Figure 9C, and Supplemental Table 8). These findings align with the results of the kinase-substrate prediction approach of phosphoproteomics data, which indicated significantly higher activity score of ROCK1 in KO cells relative to WT keratinocytes (Figure 4B and Supplemental Table 5). Based on these data, we hypothesized that RhoA signaling and its target ROCK1 (50) modulate the adhesion phenotype downstream of p120 in dermokine-KO cells. We functionally tested this possibility using the ROCK inhibitor (ROCKi) Y-27632 in dispase assays (Figure 6D). In WT and KO cells transfected with control siRNA, we did not observe a difference between ROCKi- and DMSO-treated cells (Figure 6D). Upon p120 knockdown, both WT cells and KO cells showed significantly more fragments relative to unperturbed cells when we normalized ROCKi- to DMSO-treated cells (Figure 6D). Furthermore, the number of fragments, indicating the extent of adhesion, was higher in KO than in WT cells in the latter conditions (Figure 6D). Together with data presented in Figure 5, these findings support the idea that, upon dermokine ablation, the p120-dependent adhesion phenotype is more sensitive to ROCK1 inhibition than in p120-perturbed cells alone.

Taken together, these data point to ROCK1 as a regulator of cellular adhesion downstream of the dermokine-p120 system in keratinocytes.

### Dermokine expression is reduced in the hyperthickened epidermis of non-healing wounds.

**Figure 7:**
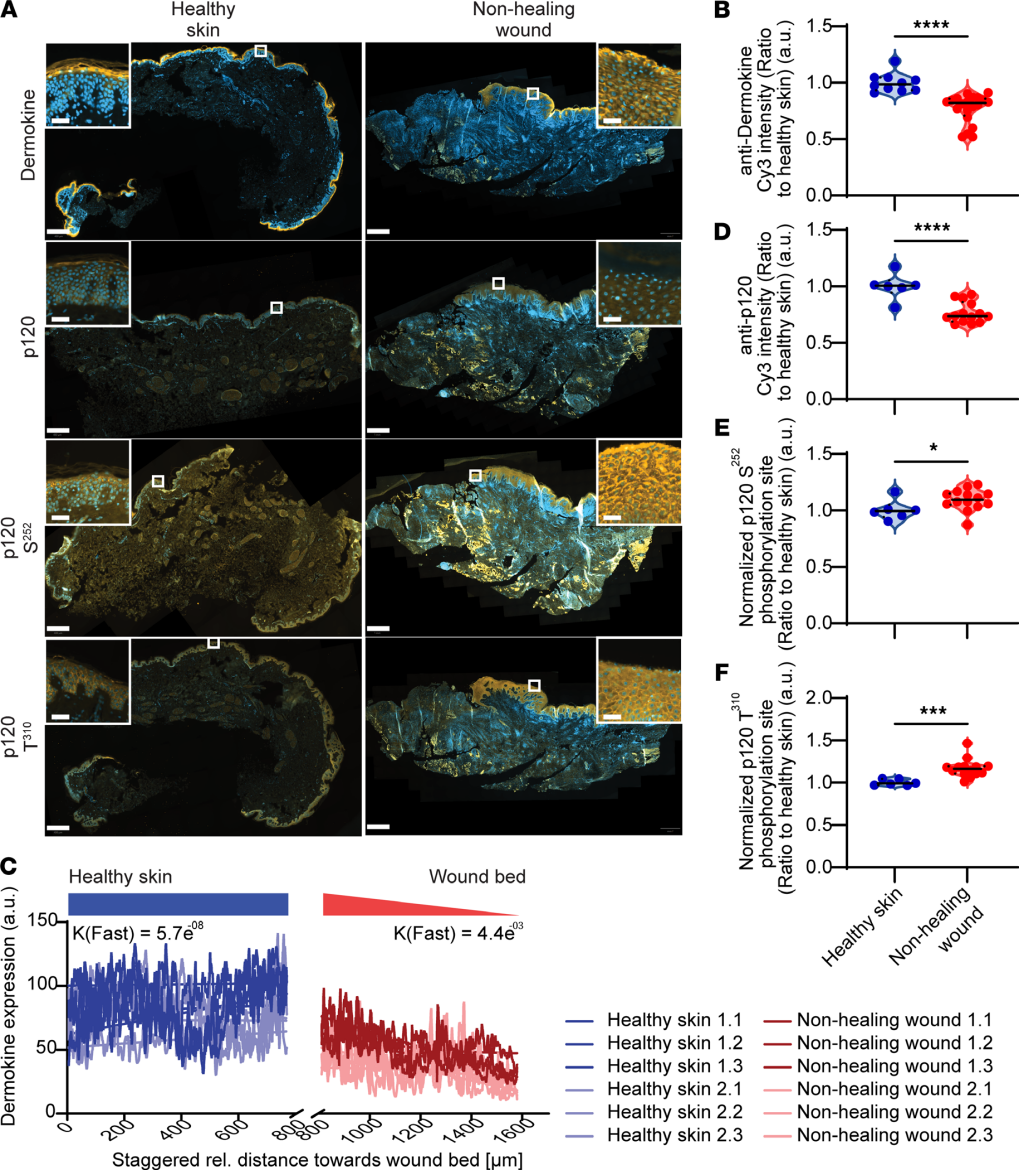
Dermokine expression decreases and p120 phosphorylation increases in keratinocytes of non-healing wounds. (**A**) Representative images of immunofluorescent staining of biopsies from healthy skin around the breast tissue from healthy individuals and non-healing wounds of venous leg ulcers (Supplemental Table 6). Scale bars: 400 μm for healthy skin, 1 mm for non-healing wounds, 50 μm for magnified images. (**B** and **D**–**F**) Quantification of immunofluorescent staining with the indicated antibodies from **A**. Anti-dermokine antibody values are the median Cy3 intensity values of *N* = 30 samples of 4 patients (non-healing wounds) and of 6 healthy individuals (healthy skin). Anti-p120 and anti–phosphorylated p120 antibody values are median Cy3 intensity values of *N* = 20 samples of 3 patients (non-healing wounds) and 3 patients (healthy skin). *P < 0.05, ***P < 0.001, ****P < 0.0001 (1-tailed, unpaired *t* test). (**C**) Quantification of dermokine intensity across 770.25 μm from the wound edge toward healthy keratinocytes. The quantified area was measured in 3 horizontal rectangles across the epidermal profile (Supplemental Figure 9D). Values are Cy3 anti-dermokine intensities along 3 equally shaped rectangles placed within the same biological sample at 2 different anatomical sites.

As abnormal dermokine expression has been previously associated with skin disorders (51), we stained skin biopsies from healthy and psoriatic skin and poorly and well-differentiated SCCs, as well as non-healing wounds (venous leg ulcers), for dermokine and p120 (Figure 7, Supplemental Figure 10, and Supplemental Table 9). We observed dermokine expression in suprabasal keratinocytes of healthy skin, psoriasis, and poorly and well-differentiated SCCs (Supplemental Figure 10, A–C), but a much weaker staining intensity in the hyperthickened epidermis of non-healing wounds (Figure 7, A and B, and Supplemental Figure 10D). We quantified dermokine expression relative to the distance to the wound bed in chronic wounds and found that keratinocytes proximal to the wound edge showed significantly lower dermokine expression across the epidermal profile, whereas, within the same biopsy, keratinocytes further away from the wound showed no change in dermokine expression (Figure 7C and Supplemental Figure 10E). To visualize the distance that keratinocytes express dermokine relative to the wounded tissue, we fitted a nonlinear regression curve previously used to explain differences in patient data from non-healing wounds and healthy skin (52). This analysis revealed that the average rate [*K*(fast)] at which dermokine expression decreases was higher in non-healing wound-edge keratinocytes [*K*(fast) = 4.4 × 10^–3^] compared with wound-edge distal keratinocytes in the respective biopsies [*K*(fast) = 5.7 × 10^–8^] (Figure 7C). Therefore, our data show less dermokine expression only in keratinocytes adjacent to the wound bed, which is consistent with the impaired differentiation of suprabasal keratinocytes in such wounds. Next, we evaluated changes in total and phosphorylated p120. For the latter, we used antibodies against phosphorylated S252 to recapitulate the immunoblotting findings and against phosphorylated T310 quantified in the phosphoproteomics dataset (Supplemental Figure 7, D and E, and Supplemental Table 4). After normalization of phosphorylated over total levels of p120, we found a significant increase in S252- and T310-phosphorylated p120 in non-healing wounds compared with healthy skin (Figure 7, D–F, and Supplemental Figure 10F).

**Figure 8:**
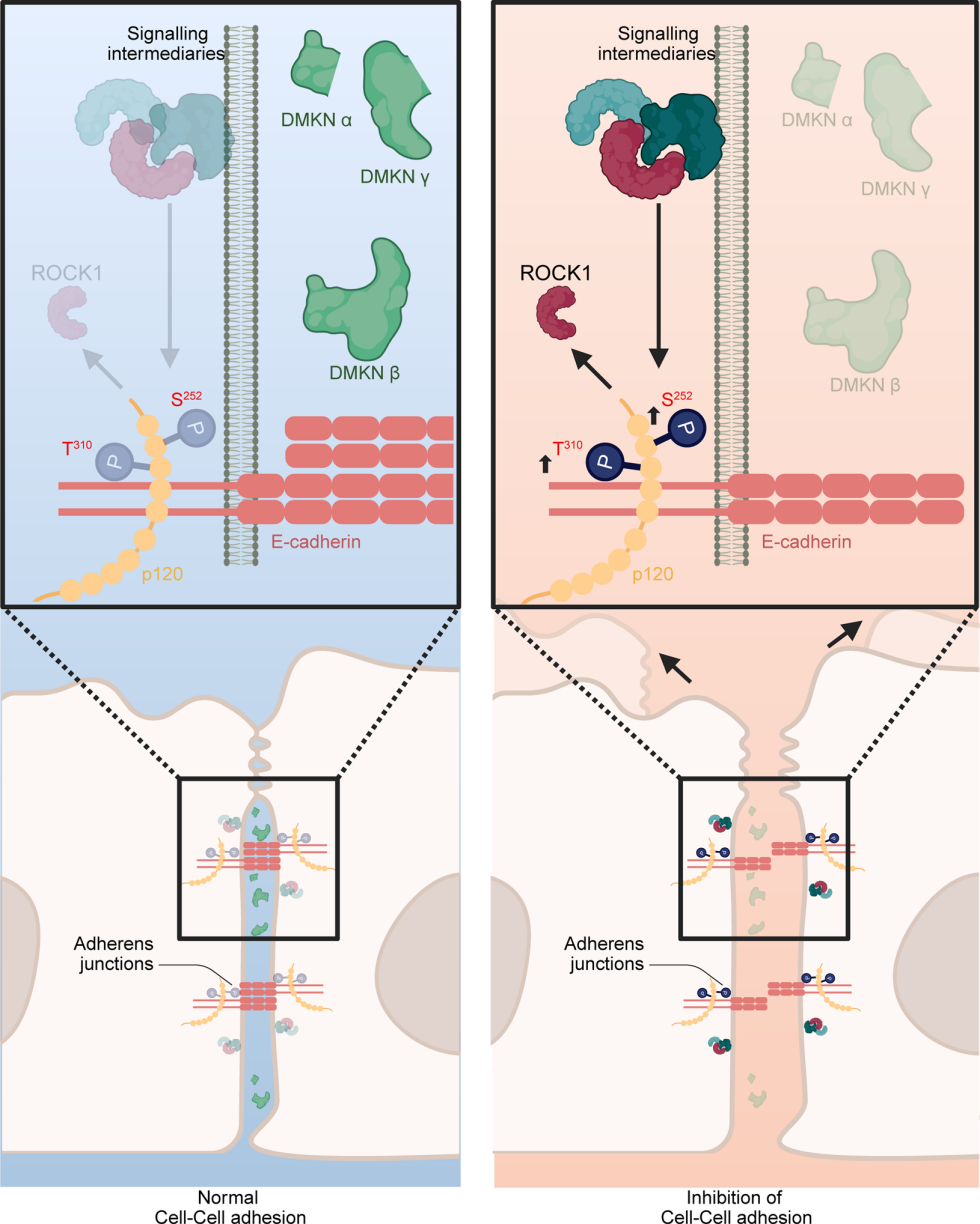
Model of dermokine function in human keratinocytes. Schematic of the putative relationship between dermokine, cell-cell adhesion, and p120 phosphorylation. Created in BioRender (biorender.com).

In conclusion, as all the tested phosphorylated sites on p120 are known to inhibit cell-cell adhesion (23, 48, 49), our results demonstrate that reduction of dermokine expression correlates with increased p120 phosphorylation, suggesting that the results obtained in vitro are also relevant to diseased skin in vivo (Figure 8).

## Discussion

This study combines genome engineering, multiproteomics analyses of 2D keratinocyte monocultures and 3D organotypic cultures, and staining of patient-derived samples to elucidate the function of dermokine in keratinocytes. Unlike earlier studies, which relied on mRNA quantification to distinguish isoforms (14), we quantified domain-specific proteotypic peptides via targeted proteomics and suggest an alternative to immunoblotting for validation of protein ablations (53). The dermokine locus encodes multiple transcripts generated via alternative splicing or promoter usage, resulting in isoforms with distinct expression patterns. While dermokine-β and -γ are highly expressed in differentiating keratinocytes, dermokine-α mRNA was detected in all epidermal layers and in the placenta (17). Genetic ablation of dermokine-βγ in mice had no effect on cutaneous wound healing but led to transient defects in cornification after birth (13). However, the expression of dermokine-α is strongly increased in the skin of these animals, potentially compensating for the loss of other isoforms. This is important, because dermokine-α has a 77.27% homology to the C-terminal domain of dermokine-β, yet it is regulated via a distinct promoter (17). Interestingly, our data revealed no major functional differences between dermokine isoforms in cultured human keratinocytes, suggesting functional overlap. The difference from the mouse data may be explained by differences in murine versus human keratinocytes. Consistent with this possibility, murine skin and human skin differ in epidermal thickness, hair follicle density, and keratinocyte biomechanics (50, 52). Alternatively, factors that are present in vivo but not in vitro may affect the function of different dermokine isoforms. A major advantage of our system is the focus on direct effects of dermokine on keratinocytes, because of the lack of vasculature and immune cells. However, effects of such cells on dermokine function should be determined in future studies, for example after transplantation of the 3D skin equivalents onto immunodeficient mice.

Dermokine, along with SBSN and KRTDAP, is part of the SSC expressed in suprabasal layers of the stratified epidermis (34). These genes are grouped in the same genomic locus and are transcribed in the same direction during epidermal development (34). Our data show that the dermokine knockout reduced the abundance of SBSN and KRTDAP, both known to be expressed during the late differentiation state of keratinocytes (34), as well as filaggrin, another late-stage differentiation marker (54). Therefore, dermokine regulates the abundance of several late-stage keratinocyte differentiation proteins essential for epidermal homeostasis, most likely through effects on keratinocyte differentiation. To uncover the function of dermokine in keratinocytes, we performed phosphoproteomics of *DMKN* αβ^–/–^, *DMKN* βγ^–/–^, and WT keratinocytes using optimized DIA assays, combined with semi-automated phosphorylated peptide enrichment, which allowed us to substantially reduce our input material from commonly used 3 to 5 mg to 200 μg lysate (38). Despite the relatively low amount of starting material, we could fully quantify almost 5,000 phosphorylated sites. This workflow will benefit other research groups who are limited by the amount of biological material, e.g., when using FFPE tissues or single cells (55, 56). Using this approach, we identified increased phosphorylation of p120 in dermokine-depleted keratinocytes. Technological developments, including the de novo sequencing tool (57), might uncover other phosphorylation sites. Functionally, impaired adhesion of dermokine-depleted keratinocytes was rescued by p120 knockdown, whereas expression of a phosphomimetic p120 mutant failed to restore it, confirming previous observations of a negative effect of p120 phosphorylation on adhesion (23, 43, 44). It is possible that phosphorylated p120 has a shorter half-life compared with the non-phosphorylated form and that the knockdown mainly affects the phosphorylated form within the experimental time frame. Our data point to a direct role of p120 phosphorylation in dermokine-dependent regulation of cell-cell adhesion both in vitro and in vivo (23, 43, 44). Although the role of p120 phosphorylation in regulating cell-cell adhesion remains controversial, Mendonsa et al. (23) showed that the phosphorylation state of p120 plays a role in the control of cadherin-1. By conversion of p120 serine/threonine sites (including T310, S268, and S252) to alanine, cell-cell adhesion was strengthened via cadherin-1–mediated binding in breast cancer cells. A possible underlying mechanism might be that the p120 armadillo repeat region, shortly after phosphorylated T310, may bind to the cadherin-1 juxtamembrane domain (23). Conceivably, the increased p120 phosphorylation in the absence of dermokine may inhibit the binding of p120 to cadherin-1 by steric hindrances and induce protein turnover by internalization of cadherin-1 via endocytosis (44). However, unchanged cadherin-1 observed by proteomics and staining of 3D organotypic cultures suggests that cadherin-1 remains present at the cell surface of the dermokine-KO cells, although its function may be affected. Our phosphomimetic and loss-of-function data identify p120 as a regulator of dermokine-dependent adhesion. Our data are consistent with prior work showing that phosphorylation of p120 on serine and threonine residues modulates adhesion (42, 58). We suggest a model in which p120 phosphorylation weakens keratinocyte adhesion by modulating Rho family kinases and downstream cytoskeletal rearrangement (59). Furthermore, we suggest that dermokine regulates signaling proteins, such as SRC, to modulate p120 phosphorylation, which in turn influences ROCK1 activity. Our phosphoproteomics data suggest that SRC, casein kinase 1ε (CSNK1E), and ERK1/ERK2, which are known to phosphorylate p120 at Y228, S268, and T310, respectively (23, 43, 60, 61), may be responsible for the dermokine/p120/cadherin-1–dependent phenotype. This hypothesis remains to be tested in future studies. Taken together, our phosphoproteomics and functional assays generate predictions that can be addressed in vivo once mice with keratinocyte-specific dermokine knockout are available.

Previous research investigating dermokine expression in human skin disorders has used an in-house anti–dermokine-βγ antibody and provided qualitative information (51). Here, we used a commercially available anti–dermokine-β and a putative dermokine-γ antibody (62) and quantified stainings in healthy and diseased human samples. Dermokine levels remained unchanged in healthy skin, psoriasis, and all SCCs, while non-healing wounds showed the strongest changes. Contrary to our findings, Hasegawa et al. showed reduced dermokine expression in poorly differentiated SCCs and increased dermokine expression in both psoriatic skin and acute wounds (51). A possible explanation for the different wound healing data might be that we stained non-healing human wounds (venous leg ulcers), while Hasegawa et al. used an acute murine wound healing model (51). Therefore, either species-specific differences or differences between acute and chronic wounds may account for these different findings. Keratinocytes at the edge of non-healing wounds fail to differentiate properly and show reduced adhesion (6, 63, 64). While dermokine expression decreased in non-healing wounds, phosphorylated p120 increased relative to healthy skin. Together with our in vitro data, these findings suggest that keratinocyte adhesion is impaired in chronic ulcers, consistent with previous studies reporting that certain cell-cell adhesion proteins are downregulated in venous leg ulcers (6). Further studies are necessary to establish whether impaired dermokine expression directly contributes to certain aspects of the chronic wound phenotype.

The functional multiomics approach used in this study improves our understanding of dermokine function and p120 phosphorylation in non-healing wounds. Our data show low dermokine expression and high p120 phosphorylation in non-healing wounds linking dermokine with cell-cell adhesion. Consistent with this, our 3D organotypic model provided cell-autonomous evidence for dermokine in keratinocytes without confounding species-specific or immunological effects. More broadly, our approach might guide drug development for cutaneous biology when integrating “omics” techniques with functional assays (65).

## Methods

Further information can be found in Supplemental Methods.

### Sex as a biological variable.

Our study examined male and female patients, and similar findings are reported for both sexes. The N/TERT-1 keratinocyte cell line used for this study is of male origin, and commercially available human dermal fibroblasts are derived from male and female donors. However, sex as a biological variable was not considered in this study.

### Cell culture.

Human keratinocyte telomerase reverse transcriptase–immortalized (h/TERT–immortalized) N/TERT-1 cells are derived from clinically normal foreskin tissue (gift from Edel O’Toole, Queen Mary University, London, United Kingdom). N/TERT-1 keratinocytes were grown in DMEM/F12 growth medium (DMEM/F12 [Thermo Fisher Scientific], 10% fetal bovine serum [FBS; Thermo Fisher Scientific], 1% penicillin/streptomycin [Sigma-Aldrich]) supplemented with RM+ medium (DMEM/F12 [Thermo Fisher Scientific], 10% FBS [Thermo Fisher Scientific], 1% penicillin/streptomycin [Sigma-Aldrich], 0.4 μg/mL hydrocortisone [Sigma-Aldrich], 0.5 μg/mL insulin [Sigma-Aldrich], 10 ng/mL epidermal growth factor [Bio-Rad], 0.1 nM cholera toxin [Sigma-Aldrich], 5 μg/mL transferrin [Sigma-Aldrich], 20 pM liothyronine [Sigma-Aldrich]) in a humidified incubator at 37°C and 5% CO_2_. Commercially available human dermal fibroblasts were purchased from Sigma-Aldrich and grown in DMEM (Thermo Fisher Scientific), 10% FBS (Thermo Fisher Scientific), and 1% penicillin/streptomycin (Sigma-Aldrich). All cell lines were tested mycoplasma negative before transfection using a commercial kit (Sigma-Aldrich). Keratinocytes were regularly tested for differentiation status using anti–keratin 14 (1:1,000; Abcam, ab7800) and anti–keratin 10 (1:150; Abcam, ab76318) antibodies, as previously published (66).

### Calcium-dependent keratinocyte assay.

Seventy-five thousand N/TERT keratinocytes were seeded into a coated 8-well chamber coverslip (Ibidi, 80806) and incubated for 24 hours. Cells were briefly washed with trypsin-EDTA (Sigma-Aldrich, T3924) and incubated 24 hours in full growth DMEM/F12 containing either 1 mM or 5 mM calcium. Cells were fixed with 4% paraformaldehyde for 15 minutes, washed with PBS, permeabilized with 0.5% Triton X-100 (Merck, X100-100ML) for 10 minutes, and blocked in 5% BSA in PBS for 60 minutes at room temperature. Cells were incubated overnight with primary antibodies anti–keratin 14 (0.1 μg/mL; Abcam, ab7800) and anti–keratin 10 (1:150; Abcam, ab76318) at 4°C. After 3 PBS washes, secondary antibodies anti-mouse Alexa Fluor 488 (1 μg/mL; Invitrogen, A11001) and anti-rabbit Alexa Fluor 568 (2 μg/mL; Invitrogen, A11011) were added for 1 hour at room temperature. Nuclei were counterstained with DAPI (4′,6-diamidino-2-phenylindole, dihydrochloride; Thermo Fisher Scientific, D1306) for 10 minutes, followed by washing and mounting (Thermo Fisher Scientific, 9990402). Coverslips were kept in the dark until imaging. Images were acquired with a Leica DMIL LED Fluo microscope. Three images per replicate were analyzed using Leica LAS X software (version 3.8.2.27713). Positively stained areas were automatically determined, and average intensity of stained area was reported. Statistical analysis was performed in GraphPad Prism (Dotmatics) using an unpaired 2-tailed *t* test.

### Guide RNA design.

Guide RNAs (gRNAs) were designed using the general settings of the CHOPCHOP web tool (https://bio.tools/chopchop) to target specific sequences associated with the isoforms of the dermokine gene.

For the chemical transfection approach, we used gRNA 1, targeting exon 2. For the atomic force microscopy–based approach, we used a Cas9 strategy to delete exon 18.

### Chemical transfection to generate DMKN βγ^–/–^ keratinocytes.

Gene editing was performed using a 2-plasmid system enabling intracellular assembly of Cas9 and DMKN-targeting gRNA (GGATACCCCGGAAACTCAGC). The Cas9 expression plasmid (CAS9PBKS, Addgene) encodes Cas9 in frame with GFP via a 2A peptide (Cas9-2A-GFP). The gRNA expression plasmid was reconstructed by cloning of *DMKN*-targeting gRNAs (Macrogen) into the U6GRNA (Addgene) vector. The vector was linearized with Bbs1, and annealed oligonucleotides were annealed using T4 ligase (New England Biolabs). Following ampicillin selection in *E*. *coli*, successful insertion was confirmed by Sanger sequencing (Macrogen). Keratinocytes were seeded at 500,000 cells/mL in 6-well plates 1 day before transfection, to achieve 50%–80% confluence. For each transfection, 1 μg of CAS9PBKS plasmid and 1 μg of gRNA plasmid were combined in 200 μL of JetOPTIMUS buffer with 2 μL of JetOPTIMUS reagent (Sartorius), incubated at room temperature for 10 minutes, and applied to keratinocytes. Two days after transfection, GFP-positive cells were sorted by flow cytometry using an MA900 cell sorter (Sony Biotechnology), with non-transfected keratinocytes used to define gating. The GFP-high (top 10%–15%) population was collected, cultured for 2 weeks, and single-cell-sorted into 96-well plates. Genomic DNA was extracted from 10,000 cells using QuickExtract DNA extraction solution (LGC Biosearch Technologies). Cells were washed with PBS, resuspended in 30–50 μL of QuickExtract DNA extraction solution, incubated at 70°C for 20 minutes, and heated to 95°C for 10 minutes. PCR was performed using PROFILase 2× Master Mix (COBO Technologies) with the following primers: forward, TCATTCTGGTTGCTGGCTCT; reverse TCTACCAGGGTCAGAGATGGT. Amplification was carried out on an ABI Veriti thermal cycler (Thermo Fisher Scientific). PCR products were purified with ExoSAP-IT and submitted to Macrogen for Sanger sequencing. Editing efficiency and genotype composition were analyzed using the Synthego ICE tool.

### FluidFM-based nano-injection to generate DMKN αβ^–/–^ keratinocytes.

The principle of FluidFM has been described previously (24), including its use for multiplex editing in CHO-K1 cells (27). Single keratinocytes were seeded into the center of a well of 12-well plates using a DispenCell (SEED Biosciences) and allowed to adhere overnight. Fifty to one hundred femtoliters of ribonucleoprotein solution was injected into the nucleus of single cells using FluidFM. The injection mixture contained 0.5 pmol gRNA (TCATCACTGCAGAAACGTGC; Integrated DNA Technologies), 0.366 pmol Cas9 (Integrated DNA Technologies), and 20 ng/μL GFP mRNA (Miltenyi Biotec). Successful injections were monitored 24 hours later by fluorescent microscopy. Single cells were clonally expanded. To improve outgrowth, untreated keratinocytes were seeded into coculture inserts within the same wells. When colonies reached 1,000 cells, cultures were rinsed with PBS, briefly incubated (3 minutes) in PBS/0.001 M EDTA, detached with TrypLE (Thermo Fisher Scientific; 15 minutes), resuspended, and re-seeded into the same well. After 48–72 hours, cells were detached and split at a 1:3 ratio into a 12-well plate. A third of the cells were collected by genomic DNA isolation using QuickExtract (Lucigen). PCR amplification was performed using the Platinum Taq DNA polymerase and GC enhancer (Thermo Fisher Scientific) with 1 μL of DNA lysate under the following conditions: (a) 95°C, 240 seconds; (b) 95°C, 30 seconds; (c) 67.1°C, 30 seconds; (d) 72°C, 45 seconds; steps b–d were repeated 35 times. Primers (0.4 μM each) were as follows: forward, ACGGTCCAAGTGGAGAAGCCGT; reverse, TCCTGCCCTCAAGACCTCTGCC. PCR products were analyzed on 1.5% (wt/vol) agarose/TAE gels at 100 V for 25 minutes alongside a 100 bp ladder (GeneRuler). For Sanger sequencing, 7.5 μL of PCR product (50–300 ng) was purified using ExoSAPIT (Thermo Fisher Scientific), adjusted to 12 μL with ultrapure water, mixed with 3 μL of forward primer (20 μM), and submitted to Microsynth for sequencing.

### Analysis of CRISPR/Cas9-mediated genome engineering.

N/TERT-1 human keratinocytes edited by either FluidFM or chemical transfection were validated by Sanger sequencing as described above. Trace files were aligned to the Ensembl target sequence using Benchling, and chromatograms complementary to the gRNA were inspected for overlapping peaks or deletions indicative of indel formation. Sequencing files from selected clones were deconvoluted using Indigo (Gear Genomics), and individual allele sequences were aligned to the control sequence to visualize the deletions or insertions. Clones carrying frameshift mutations (indels not divisible by 3) in all alleles were considered knockouts (Supplemental Figure 1).

### Generation of human DMKN αβ^–/–^ and DMKN βγ^–/–^ keratinocyte 3D organotypic skin cultures.

We generated 3D organotypic skin cultures by placing 600 μL of collagen/Matrigel (Corning) matrix containing 100,000 human dermal fibroblasts (Sigma-Aldrich) (210 μL collagen I [Corning], 210 μL Matrigel [Corning], 59 μL 10× MEM [Thermo Fisher Scientific], 59 μL FBS [Thermo Fisher Scientific], and 59 μL fibroblasts [Sigma-Aldrich]) into a new 12-well plate Transwell insert (Sigma-Aldrich). After polymerization for 1 hour at 37°C, 59 μL of either WT, *DMKN* αβ^–/–^, or *DMKN* βγ^–/–^ keratinocytes (1,000,000 cells) were seeded onto the matrix, and RM+ medium was added below the insert. After overnight incubation at 37°C, cultures were airlifted and grown at the air-liquid interface for 15 days with daily medium changes. After 15 days, the 3D cultures were cut in half; one half was fixed in 4% paraformaldehyde (Sigma-Aldrich) for 30 minutes at room temperature and paraffin-embedded for IHC analysis, and the other half was snap-frozen in liquid nitrogen for proteomics analysis.

### Hematoxylin and eosin and IHC staining of WT, DMKN αβ^–/–^, and DMKN βγ^–/–^ 3D organotypic skin cultures.

DMKN-KO and WT 3D organotypic skin culture sections were stained with hematoxylin (Sigma-Aldrich) for 5 minutes and washed in tap water for 5 minutes, followed by eosin (Sigma-Aldrich) for 5 minutes and another 5-minute tap water wash. Sections were dehydrated with 96% and 99% ethanol (Sigma-Aldrich), air-dried, and mounted with Pertex (Histoline).

Paraffin sections (4 μm; Shandon microtome) were dried overnight at 35°C. IHC staining followed the manufacturer’s instructions (Thermo Fisher Scientific). Sections were heated at 60°C for 60 minutes, deparaffinized, rehydrated, and blocked with Ultra V Block (Thermo Fisher Scientific) for 5 minutes. Slides incubated with anti-dermokine antibody (1:100; Abcam) were directly treated for 1 hour and rinsed with TBS (Sigma-Aldrich). Slides stained with anti–Ki-67 (1:200; Abcam) or anti-ITGα6 (1:500; Abcam) were pretreated in EDTA buffer (10 mM Tris, 1 mM EDTA, pH 9.0). Slides stained with anti–cadherin-1 (1:50; BD Biosciences), anti-KRT10 (1:1,000; Abcam), or anti-KRT14 (1:500; Abcam) antibodies were pretreated in citrate buffer (100 mM citric acid, 100 mM sodium citrate, pH 6.0). All slides were boiled in EDTA or citrate buffer for 15 minutes, cooled for 15 minutes, and washed twice.

Primary antibody enhancer (Thermo Fisher Scientific) was applied for 10 minutes followed by three 5-minute washes. HRP polymer (Thermo Fisher Scientific) was applied for 15 minutes and washed similarly. Slides were developed with DAB (Thermo Fisher Scientific) for 10 minutes, washed, stained with hematoxylin (Sigma-Aldrich) for 15 seconds, rinsed for 5 minutes, and mounted in Pertex (Histoline). The images were acquired with an EVOS M5000 microscope (×20 magnification; Thermo Fisher Scientific).

Semiquantitative IHC analysis was performed in QuPath (version 0.5.0; https://qupath.github.io). Entire epidermal-like regions were drawn manually, DAB signal intensities were quantified using a pixel-based thresholder, and values were visualized in GraphPad Prism.

### Immunofluorescence and quantification of patient tissue.

FFPE tissue samples from healthy skin, SCCs, wounds, and psoriasis were selected and retrieved from the SKINTEGRITY.CH biobank of the Department of Dermatology, University Hospital Zurich. The blocks were used to prepare 7 μm tissue sections and stained. Patient information is available in Supplemental Table 9.

FFPE sections were dried for 30–60 minutes at 60°C before being dewaxed, rehydrated, and PBS-buffered. Antigen retrieval was performed with 0.1 M sodium citrate buffer (pH 6) for 45 minutes at 95°C. After 3 washes with PBST (PBS plus 0.1% Tween), unspecific binding to tissue sections was blocked for 1 hour at room temperature with 12% BSA in PBST. Primary antibody was diluted (catenin δ-1 [1:200; Cell Signaling Technology], dermokine [1:250; Abcam], phospho–catenin δ-1 Thr310 [1:250; Abcam], and phospho–catenin δ-1 Ser252 [1:250; Cell Signaling Technology]) in blocking buffer and incubated overnight at 4°C. After 3 washes, slides were incubated for 1 hour at room temperature with the secondary antibody (anti-rabbit IgG–Cy3, 1:200) and 1 μg/mL Hoechst 33342 (Sigma-Aldrich) in blocking buffer. After another 3 washes, sections were mounted using Mowiol (Sigma-Aldrich) with DABCO (Sigma-Aldrich). Slides were scanned with an Axioscan 7 slide scanner (Carl Zeiss AG).

Staining intensity was quantified using QuPath (version 0.5.0). Positively stained area was determined using a pixel thresholder, and median intensity of stained area was reported. Data were analyzed in GraphPad Prism.

### Statistics.

All experiments were repeated at least 3 times as independent biological replicates with similar results. Representative Western blots or immunofluorescence or IHC images are shown. The number of independent experiments, treatments, and relative controls and statistical analysis are indicated in the figure legends. *P* < 0.05 was considered significant. *P* values were calculated using a 2-tailed Student’s *t* test. One- or two-way ANOVA was used with post hoc tests as indicated in the figure legends.

### Study approval.

All patient-derived samples used were surplus material from routine surgeries and were provided by the Dermatology Department of the University Hospital Zurich, Switzerland, with the assistance of the SKINTEGRITY.CH biobank. The surplus biopsies stored in the biobank were from consenting patients. The use of material for research purposes was approved by the local ethics commissions (Cantonal Ethic Commission Zurich, project 2017-00684). All experiments conformed to the principles set out in the World Medical Association’s Declaration of Helsinki and the US Department of Health and Human Services Belmont Report. All human cell line experiments were performed with the approval of the Technical University of Denmark.

### Data availability.

The mass spectrometry proteomics data were deposited to the ProteomeXchange Consortium through the PRIDE partner repository with accession codes PXD050151, PXD055812, and PXD074943. The targeted proteomics data were uploaded to the Panorama repository, available via the Panorama Public DOI (doi.org/10.6069/hgvx-qe48).

This paper reports original code (https://github.com/VahapCan/Dermokine_Analysis_2026; commit ID b35065c). Underlying data points are available in the Supporting Data Values file.

## Author contributions

UADK conceived the project and acquired funding, now administered by CF. UADK, CF, JD, SG, RBD, and VC designed the experiments. VC, TW, WT, TAB, AMH, SM, CC, MS, GR, and CRE performed the experiments. JH provided patient samples. CF and VC wrote the manuscript. All authors read, revised, and approved the manuscript.

## Conflict of interest

TAB, AMH, and SM are employees of Cytosurge AG. TAB owns shares in Cytosurge AG.

## Funding support

- Wellcome Trust (107636/Z/15/Z and 107636/Z/15/A) to CF.
- Novo Nordisk Foundation Young Investigator Award (call 2022, NNF22OC0070845) to CF.
- Novo Nordisk Foundation Young Investigator Award (call 2016, NNF16OC0020670) to UADK.
- LEO Foundation (LF-OC-23-001220) to CF.
- LEO Foundation (LF-OC-19-000033) to UADK.

## Supplementary Material

Supplemental data

Unedited blot and gel images

Supplemental table 1

Supplemental table 2

Supplemental table 3

Supplemental table 4

Supplemental table 5

Supplemental table 6

Supplemental table 7

Supplemental table 8

Supplemental table 9

Supporting data values

## Version 1 04/23/2026

In-Press Preview

## Version 2 07/08/2026

Electronic publication
