# Supplementary material for: Multiomics analysis reveals dermokine as a regulator of keratinocyte differentiation and adhesion
Source: JCI Insight. 2026 Apr 23;11(13):e197838. doi: 10.1172/jci.insight.197838 (PMC13460826; doi:10.1172/jci.insight.197838)
Supplement: Supplementary file 1 [file jciinsight-11-197838-s065.pdf]

**Supplementary Information for**

**Multi-omics analysis reveals dermokine as a regulator of keratinocyte differentiation and adhesion**

Vahap Canbay[1], Till Wüstemann[2], Weihua Tian[1], Tobias A. Beyer[3], Camilla Reiter Elbæk[1], Michael Stumpe[4], Gaetana Restivo[5], Chatpakorn Christiansen[1], Anabel Migenda Herranz[3], Susanne Mailand[3], Jürg Hafner[5], Rune Busk Damgaard[1], Steffen Goletz[1], Jörn Dengjel[4], Ulrich auf dem Keller[1, t] and Chiara Francavilla[1, 6, *]

[1]Department for Bioengineering and Biomedicine, Technical University of Denmark, 2800, Copenhagen, Denmark.

[2]Department of Biology, Swiss Federal Institute of Technology Zurich, 8152, Zurich, Switzerland.

[3]Cytosurge AG, Glattbrugg, 8152, Zurich, Switzerland.

[4]Department of Biology, University of Fribourg, 1700, Fribourg, Switzerland.

[5]Department of Dermatology, University Hospital Zurich, 8091, Zurich, Switzerland

[6]Division of Cellular and Molecular Function, FBMH, The University of Manchester, M139PT, United Kingdom

[t]Deceased

*Corresponding author: Chiara Francavilla. Department for Bioengineering and Biomedicine, Technical University of Denmark, Søitofts Plads, 2800, Kongens Lyngby, Denmark. Phone: 004593511641 E-mail: chiafra@dtu.dk

This document file includes:

Supplemental Methods

Supplemental Figures 1-10 and Legends

Supplemental Table Legends 1-9

Supplemental References

## Supplemental Methods

### Mass spectrometry (MS)-based proteomics

Lysis buffer (4M guanidine hydrochloride (GuHCl) and 250 mM N-2-hydroxyethylpiperazine-N-2-ethane sulfonic acid (HEPES) (pH 7.8)) was added to 3D organotypic skin cultures, which were then homogenized using the steel beads (QIAGEN) and TissueLyser II (QIAGEN). The lysate was processed in the TissueLayser twice for 1 min each at varying frequencies (3 Hz to 30 Hz), beads were removed using a magnetic rack and the suspension was heated for 5 min at 95 °C. Following this, the samples underwent five cycles of sonication (30 Hertz for 30 seconds each) and cooling using the TissueLyser ii (QIAGEN). After centrifugation (13,000 *x g* for 15 minutes at 4 °C), the protein content was determined using both the Nanodrop (Thermo Fisher Scientific) and Quickstart Bradford Protein assay (Bio-Rad; Cat#5000001). The samples were reduced and alkylated on aliquots of 50 µg protein using 5 mM tris(2-carboxyethyl)phosphin (TCEP) and 20 mM 2-chloroacetamide (CAA). The samples were diluted with 50 mM HEPES buffer (pH 7.8) and incubated with Lys-C (FUJIFILM) for 4 h, 37 °C followed by trypsin (Promega) for 16 h at 37 °C. The enzyme activity was blocked by 1% tri-fluoroacetic acid (TFA) and samples were desalted using Solaµ plates (Thermo Fisher Scientific), according to manufacturer's instructions.

For each resuspended proteome, peptides were analyzed using the pre-installed '30 samples per day' method on the EvoSep One instrument, operating a 44-minute gradient. Peptides were eluted over an EvoSep One defined 44-min gradient and analyzed by mass spectrometry, Orbitrap Exploris 480 (Thermo Fisher Scientific), adapted from with the following settings: Spray voltage was set to 2.3 kV, funnel RF level at 40, and heated capillary at 240 °C. Full MS spectra were collected at a resolution of 120000, with an AGC target of 300% or maximum injection time set to 'custom' and a scan range of 350–1650 m/z. The $MS^2$ spectra were obtained in DIA mode in the Orbitrap operating at a resolution of 15000, equipped with FAIMS Pro™ Interface (Thermo Fisher Scientific) with a CV of -35, -55 and -75 V, with an AGC target 1000% or maximum injection time set to 'auto', a normalized HCD collision energy of 30. The isolation window was set to 38 m/z with a 1 m/z overlap and window placement off. MS performance was verified for consistency by running 60-minute gradient in-house HeLa complex cell lysate quality control standards - 20 ng and 200 ng - and chromatographic elution of the peptides was monitored to check for reproducibility, considering the homogenous distribution over the chromatographic column.

Raw files were analyzed using Spectronaut™ (Biognosys) directDIA+ (deep) default settings, spectra were matched against the homo sapiens reference FASTA database (UP000005640; downloaded from Uniprot on 27th of December 2022). Default settings were used without imputation. Dynamic modifications were set as Oxidation (M), Glutamine to pyro-Glutamine and Acetyl on protein N-termini. Cysteine carbamidomethyl was set as a static modification. Protein quantitation was done on the $MS^2$ level, data filtering set to default Q-values (1 % FDR). Data were processed using R (version 4.1.1) and functions implemented in the pipeline of limma (version 1.30.7). The volcano plots were visualized using ggplot2 (1) and the Gene Ontology analysis was performed using Metascape (2).

The methods used to acquire and analyze the p120 perturbed proteome were slightly modified. Peptides were eluted using a 58-minute gradient on the EvoSep One instrument and analyzed using the Orbitrap Eclipse mass spectrometer (Thermo Fisher Scientific). All settings for full MS spectra acquisition were kept the same apart from a scan range of 400 – 1200 m/z. Operating in DIA mode, we changed the $MS^2$ resolution to 60000, scan range 200 – 1200 m/z, maximum injection time to 118 ms and the HCD value to 28 %. We changed the CV value to a single -50 V. The isolation window was set to 8 m/z with a 1 m/z overlap and window placement optimization on. The maximum number of scan events was set to 99, loop to 3 s and loop count to 25.

Raw files were analyzed as before using Spectronaut™ (Biognosys). Results were processed in R (version 4.1.1). Gene ontology analysis was performed using Metascape and each exported term a module-dependent p value was calculated using Stouffer's Z method. Resulting p values were adjusted for multiple testing using Benjamini Hochberg corrections.

**Targeted proteomics of proteotypic dermokine peptides**

Peptides (500 ng and 0.25 pmol for each heavy peptide) from *DMKN* $\alpha\beta^{-/-}$, *DMKN* $\beta\gamma^{-/-}$ and WT 3D organotypic skin cultures were loaded onto a 2 cm C18 trap column (Thermo Fisher Scientific), connected in-line to a 50 cm C18 reverse-phase analytical column (Thermo Fisher Scientific) using 100% Buffer A (0.1% Formic acid in water) at 750 bar, using the EasyLC 1200 HPLC system (Thermo Fisher Scientific), and the column oven operating at 30 °C. Peptides were eluted over a 70-minute gradient ranging from 10% to 95% of 80% acetonitrile, 0.1% formic acid at 250 nL/min, and the Q-Exactive instrument (Thermo Fisher Scientific) was run in scheduled parallel reaction monitoring (PRM) mode for the *DMKN* $\beta\gamma^{-/-}$ 3D organotypic skin cultures. Full MS spectra were collected at a resolution of 70000, with an AGC target of $3e^6$ or maximum injection time of

10 ms and a scan range of 350–1400 m/z. The $MS^2$ spectra were obtained at a resolution of 35000, with an AGC target value of $1e^6$ or maximum injection time of 128 ms, a normalized collision energy of 27. For the *DMKN* $\alpha\beta^{-/-}$ 3D organotypic skin cultures the $MS^2$ spectra were obtained at a resolution of 17500. MS performance was verified for consistency by running complex cell lysate quality control standards, and chromatography was monitored to check for reproducibility.

Skyline (version 22.2.0.351) was used to analyze the PRM data(3). Target peptides were imported after setting filter parameters at $MS^1$ precursor mass analyzer to orbitrap, resolving power of 70000 at 400 m/z and $MS^2$ acquisition method set to PRM orbitrap resulting power of 30000 at 400 m/z. Endogenous peptides had a fixed modification of carbamido-methylation of cysteines (+57.021 Da). In addition, heavy peptides had fixed modifications of heavy lysine and heavy arginine amino acids on their C-terminus (+8.014 and +10.008 Da). Both an *in-silico* spectral library was generated via Prosit(4) and a manual spectral library was created using the same instrument setup in DDA mode. The dermokine-proteotypic peptide – LGFINWDAINK – was removed from data analysis as well as - FGTNTQGAVAQPGYGS. The transition peak areas and their boundaries were manually adjusted and derived from the heavy peptide retention times. Endogenous peptides were normalized based on their ratio to heavy peptides. Final calculations were based on the sum of transition areas. The $\log_2$ transformed fold changes of summed total dermokine transition areas were calculated.

**Mass spectrometry-based DIA phosphoproteomics and proteomics**

*DMKN* $\alpha\beta^{-/-}$, *DMKN* $\beta\gamma^{-/-}$ and WT keratinocytes were washed three times with PBS (PAN Biotech) and incubated in DMEM/F-12 (Thermo Fisher Scientific) without FBS for 24 h. *DMKN* $\alpha\beta^{-/-}$, *DMKN* $\beta\gamma^{-/-}$ and WT keratinocytes were either incubated with buffer (25 mM Tris-HCl, 100 mM glycine, pH 7.3, 10% glycerol) or treated with recombinant dermokine (50 ng/mL) for 1 h. Cells were scraped from the dishes and the cell suspension was transferred into a falcon tube and washed three times before frozen in liquid frozen in liquid nitrogen. After thawing, cells were immediately resuspended in 8 M Urea and 50 mM Tris-HCl (pH 8.0). 200 µg lysate, determined by a BCA kit (Thermo Fisher Scientific), per sample were reduced with dithiothreitol (DTT) (Sigma-Aldrich) (1 mM final concentration), alkylated by iodoacetamide (Sigma-Aldrich) (5 mM final concentration), and digested with Lys-C (FUJIFILM) in an enzyme/protein ratio of 1:100 (w/w) for 2 h, shaking, followed by an 8-fold dilution with 50 mM Tris-HCl (Sigma-Aldrich) (pH 8.0) to 1 M Urea (Sigma-Aldrich) and further digested overnight with trypsin (Promega) 1:100 (w/w). Protease activity was quenched by acidification with TFA (Sigma-Aldrich) to a final concentration of 1% and peptides were purified by SPE using HR-X columns

(Macherey-Nagel) in combination with C18 cartridges (Macherey-Nagel). The peptides were frozen in liquid nitrogen and lyophilized prior to phosphopeptide enrichment. The enrichment of phosphopeptides was automatically performed on a Bravo Automated Liquid Handling Platform (Agilent). The Fe (III)-NTA cartridges (Agilent) (5 µL) were initially primed with 200 µL 0.1% TFA (Sigma-Aldrich) in acetonitrile and then balanced with 0.1% TFA in 80% acetonitrile (Thermo Fisher Scientific) (equilibration/washing buffer). Peptides were redissolved in 200 µL of equilibration buffer and loaded onto the cartridges at a flow rate of 5 µL/min. Cartridges were then rinsed twice with 200 µL of washing buffer at a flow rate of 10 µL/min. Phosphopeptides were extracted first with 50 µL of 1% ammonia followed by 50 µL of 1% ammonia in 80% acetonitrile (Thermo Fisher Scientific) at a flow rate of 5 µL/min into 5 µL of 99% formic acid (Thermo Fisher Scientific). These samples were then concentrated via a lyophilizer and reconstituted in 20 µL of 0.1% formic acid (Thermo Fisher Scientific) for subsequent LC-MS/MS analysis.

Both the proteome and phosphoproteome of each condition, collected in triplicates, have been acquired using $HRMS^1$-DIA methods with a mass range from either 400 – 1000 m/z and 58 min for the proteome or 400 – 1400 m/z for the phosphoproteome and 140 min.

For each resuspended proteome (200 ng), peptides were analyzed using the '20 samples per day' method on the EvoSep One instrument (EvoSep). Peptides were eluted over a defined 58-min gradient and analyzed by an Orbitrap Exploris 480 mass spectrometer (Thermo Fisher Scientific), with following settings: Spray voltage was set to 2.3 kV, funnel RF level at 40, and heated capillary at 240 °C. Full MS spectra were collected at a resolution of 120000, with an AGC target of 300% or maximum injection time set to 'auto' and a scan range of 400–1000 m/z. The $MS^2$ spectra were obtained in DIA mode in the Orbitrap operating at a resolution of 30000, with an AGC target 1000% or maximum injection time set to 'auto', a normalized HCD collision energy of 32. The isolation window was set to 8 m/z with a 1 m/z overlap and window placement on. Each DIA experiment covered a range of 200 m/z resulting in three DIA experiments (400-600 m/z, 600-800 m/z and 800-1000 m/z). Between the DIA experiments a full MS scan was performed. MS performance was verified for consistency by running complex cell lysate quality control standards, and chromatography was monitored to check for reproducibility.

Each resuspended enriched keratinocyte phosphoproteome (1 µg) was analyzed with an Easy-nLC 1200 (Thermo Fisher Scientific) coupled to an Orbitrap Exploris 480 mass spectrometer (Thermo Fisher Scientific)

operated with a DIA method; 5 µL of each sample was injected. Peptides were separated on a fused silica HPLC-column tip (I.D. 75 µm, self-packed with ReproSil-Pur 120 C18-AQ, 1.9 µm (Dr. Maisch) to a length of 20 cm) using a gradient of mobile phase A (0.1% formic acid in water) and mobile phase B (0.1% formic acid in 80% acetonitrile in water). The column temperature was maintained at 50 °C using the EASYSpray oven. The total gradient time was 140 min and went from 6% to 23% acetonitrile (ACN) in 95 min, followed by 30 min to 38%. This was followed by 5 min increase to 60% ACN, and a washout by 10 min increase to 90% ACN. Flow rate was kept at 250 nL/min. Re-equilibration was done prior to sample pickup and prior to loading with a volume of 4 µL of 0.1% FA buffer at a maximum pressure of 750 bar. Spray voltage was set to 1.9 kV, funnel RF level at 40, and heated capillary at 275 °C. Full MS spectra were collected at a resolution of 120000, with an AGC target of 300% or maximum injection time set to 'auto' and a scan range of 400–1400 m/z. The $MS^2$ spectra were obtained in DIA mode in the Orbitrap operating at a resolution of 60000, with an AGC target 1000% or maximum injection time set to 'auto' and a normalized HCD collision energy of 32. The isolation window was set to 6 m/z with a 1 m/z overlap and window placement on, resulting in 175 DIA windows. Each DIA experiment covered a range of 200 m/z resulting in three DIA experiments (400-600 m/z, 600-800 m/z, 800-1000 m/z, 1000-1200 m/z and 1200-1400 m/z). Between the DIA experiments, a full MS scan was performed. All data were acquired in profile mode using positive polarity, application mode was set to peptide, FAIMS mode was not installed, advanced peak determination was set to true, and default charge state was set to 2. MS performance was verified for consistency by running complex cell lysate quality control standards, and chromatography was monitored to check for reproducibility. The median peak width was 0.91 min, and the full width at half maximum was 0.53 min. Each $MS^1$ window had 175 $MS^2$ DIA windows, achieving a 5.17 s cycle time and 10 median data points per peak on $MS^1$ level with a 140 min gradient length.

Two independent searches for the proteome and phosphopeptide samples were performed. For the proteome, raw files were analyzed using Spectronaut™ (Biognosys, version 18.5) directDIA+ (deep) default settings, spectra were matched against the homo sapiens reference FASTA database (UP000005640; downloaded from Uniprot on 27th of December 2022). Dynamic modifications were set as Oxidation (M) and Acetyl on protein N-termini. Cysteine carbamidomethyl was set as a static modification. Protein quantitation was done on the $MS^1$ level, data filtering set to Q-value and the data were normalized by RT-dependent local regression model and protein groups were inferred by IDPicker. For phosphopeptides, raw files were analyzed using Spectronaut™ (Biognosys, version 17.4) directDIA+ (deep), spectra were matched against the homo sapiens

database (UP000005640; downloaded from Uniprot on 27th of December 2022) with the BGS Phospho PTM workflow with the following modifications: deamidation (NQ) and oxidation (M) were added as dynamic modifications together with Phospho (STY) and Acetyl on protein N-termini. Cysteine carbamidomethyl was set as a static modification. The proteomics Supplemental Table was run-wise imputed via Spectronaut™ (Biognosys). All results were filtered to a 1% FDR, data filtering set to Q-value and protein quantitation done on the $MS^1$ level. The data was cross-normalized by a RT-dependent local regression model on modified (phospho)-peptides. The PTM probability cutoff was set to 0.

The phosphoproteomic data analysis was performed using modified function from the “PhosR” package embedded in custom scripts in R (version 4.3.2). Data at protein level was processed using R (version 4.3.2). The Supplemented WT samples were not considered for any further analysis due to the high coefficient of variation. Due to the nature of DIA, all peptides were acquired, recording single low-intense phosphorylated precursors (singletons)(5). To remove singletons, we excluded phosphorylated peptides that contained low quality chromatography peaks (>0.6), utilizing Spectronaut’s ability to evaluate the shape of chromatographic peaks (FG.ShapeQualityScore ($MS^1$)). We then imported the filtered phosphorylated peptides into MaxQuant and assigned site localization probabilities.

For the analysis only peptides with a site localization probability threshold of at least 0.75 were considered in the bioinformatic analysis(6). Next, we excluded phosphorylated sites that were less than 70% present in at least one condition. Finally, we removed phosphorylated sites that contained any missing value after removing all phosphorylated sites with any missing values we ended up with 4453 fully quantifiable phosphorylated sites, including 299 tyrosine phosphorylated sites without specific enrichment methods. For kinase-substrate enrichment analysis using data shown in Figure 3, the filtered Spectronaut™ (Biognosys, version 18.5) reports were transformed into modification-specific peptide-like reports using the plugin peptide collapse in Perseus (version 2.0.9.0) with “EG.PTMAssayProbability” as grouping column. A localization cutoff of 0.75 was applied and the same variable PTMs as listed above, and summed intensities were $log_2$-transformed. No imputation was performed in the phosphoproteomics Supplemental Table . The limma package was employed to calculate the fold change as well as the moderated p-value for the comparisons between genotypes for both the phosphorylated peptides and the $log_2$-transformed protein abundance values. To analyze the proteome, we used the limma package in R and the volcano plots were plotted using ggplot2. Gene ontology analysis was performed using Metascape. The phosphoproteomics Supplemental Table s were aligned with upstream

kinases by inferring kinase activity through clustering phosphorylated sites based on either their known kinase targets (profile score) or their common phosphorylation motifs (motif score), following the Kinase-Substrate prediction approach(7, 8). In brief, phosphorylated sites were z-transformed and mapped to known kinases using PhosphoSitePlus® data(8). Profile and motif scores were calculated, averaged, and used to determine a combined score for each site(8). The combined scores were visualized in a heatmap and was clustered as circular signalome map(8). The kinase activity score is computing the kinase-substrate profile for each kinase by calculating the median phosphosite quantification for kinase in the quantification matrix(8). The kinases and their respective kinase activity score were tested for significance (one-way ANOVA) among all genotypes. Clusters from the circular signalome map were subjected to GO analysis, including all ontologies and showing the top 3 GO terms, via the clusterProfiler package(8).

To normalize the intensity of the phosphorylated peptide we divided the raw intensities of the phosphorylated sites through the respective protein quantities(9) . All Figure were plotted in R using ggplot2.

**Cell lysis and SDS-PAGE immunoblotting**

Cells from endogenous *DMKN* $\beta\gamma^{-/-}$, *DMKN* $\alpha\beta^{-/-}$ and WT keratinocytes as well as supplemented *DMKN* $\beta\gamma^{-/-}$, *DMKN* $\alpha\beta^{-/-}$ and WT keratinocytes were washed in ice-cold PBS once before being scraped into lysis buffer (25 mM HEPES pH 7.4, 150 mM KCl, 2 mM MgCl2, 1 mM EGTA, 0.5% (v/v) Triton X-100) supplemented with EDTA-free Protease Inhibitor Cocktail (Sigma-Aldrich), PhosSTOP (Roche), and 1 µM DTT. Lysates were centrifuged at 13000 rpm at 4 ˚C for 10 minutes. Supernatant was transferred to fresh tubes, concentrations adjusted based on Bradford assay (BioRad) to, 1 µg/µL and proteins were denatured by adding Laemmli sample buffer (LSB) followed by heating for 5 minutes at 70 ˚C. Samples were loaded on 9 % Bis-Tris gels and run at 150 V in Tris running buffer or on 4-12 % Bis-Tris gels (Invitrogen) and run in MOPS buffer (Invitrogen). Proteins were transferred onto 0.2 µM nitrocellulose membranes (BioRad) using the Trans-Blot Turbo System (BioRad), and membranes were blocked for 30 min in 5 % milk in Tris Buffered Saline with Tween 20 (TBS-T). After blocking, membranes were incubated with primary antibodies in 3% BSA-TBS-T overnight at 4 ˚C while shaking. After washing in TBS-T for 5 minutes, membranes were incubated with HRP-coupled secondary antibodies for 45 minutes at room temperature. After 3 x 5 minutes TBS-T washes, protein bands were developed using ECL Western Blotting Substrate (BioRad). Chemiluminescence was detected using BioRad's ChemiDoc Imaging Systems and software.

**Transfection and RNA interference**

Cells were cultured in DMEM/F12 media (Thermo Fisher Scientific) with Lipofectamine (Invitrogen) according to the manufacturer's instructions, and all subsequent assays were conducted 72 h post-transfection. Double-stranded, validated Stealth siRNA oligonucleotides targeting human p120 human (HSS175607 (5`-CAGCUCGAGGCUAUGAGCUCUUAUU-3`), HSS102463 (5`-GGCUAGAGGAUGACCAGCGUAGUAU-3`) and HSS102464 (5`-GCAGCUCCCAAUGUUGCCAACAAUA-3´), called siRNA#1, siRNA#2 and siRNA#3 respectively) were purchased from Invitrogen. As a negative control, duplexes with irrelevant sequences (5'-GGGAUACCUAGACGUUCUA-3', called scrambled siRNA) were purchased from Sigma-Aldrich. *DMKN* $\beta\gamma^{-/-}$, *DMKN* $\alpha\beta^{-/-}$ or WT keratinocytes were transfected either with siRNA#1, siRNA#2, siRNA#3, scrambled siRNA or lipofectamine alone. Immunoblotting, as described previously, of *DMKN* $\beta\gamma^{-/-}$, *DMKN* $\alpha\beta^{-/-}$ or WT keratinocytes transfected either with siRNA#1, siRNA#2, siRNA#3 or lipofectamine alone (control) was performed to test for p120 expression.

**Cell-cell adhesion assay**

This assay was modified and performed as described before(10). Briefly, cells from *DMKN* $\beta\gamma^{-/-}$, *DMKN* $\alpha\beta^{-/-}$ and WT keratinocytes were detached using Trypsin-EDTA (Thermo Fisher Scientific). Cells were washed with PBS, counted and resuspended in DMEM/F12 (Thermo Fisher Scientific) containing 25 mM HEPES and 1 mM $CaCl_2$ to a concentration of 200.000 cells/mL. 10 mL of each cell suspension was placed in the respective non-adhesive 100 mm dish (VWR), as previously described. The dishes were placed in an agitating incubator (Infors AG) at 37 ˚C while shaking (30 rpm) for 100 minutes. Immediately after cluster formation, 25 images were automatically captured with an EVOS M5000 (Thermo Fisher Scientific) per dish in a counterclockwise manner. Clusters of more than 4 cells from each image from each replicate were counted(10)Click or tap here to enter text. and analyzed via GraphPad Prism (Dotmatics). A one-way ANOVA was used to detect changes among the conditions and illustrated as violin plots.

As described previously, *DMKN* $\beta\gamma^{-/-}$, *DMKN* $\alpha\beta^{-/-}$ or WT keratinocytes were transfected either with siRNA#1, siRNA#2, siRNA#3 or scrambled siRNA in 6 well plates for 72 hours. The transfected cells were dissociated using Trypsin-EDTA (Thermo Fisher Scientific) and treated as described above. Immediately after the assay, 4 randomly chosen images were captured per dish to capture clusters of more than 4 cel(11). Ke. Cell cluster (≥4) were counted, summarized per replicate and each siRNA transfected *DMKN* $\alpha\beta^{-/-}$, *DMKN* $\beta\gamma^{-/-}$, WT keratinocyte (10) replicate was normalized to the average WT cell cluster number. Subsequently all normalized

siRNA cell cluster values were averaged for each genotype. A two-way ANOVA and a Fishers Least Significant Difference test was used to show changes among the conditions.

**Dispase-based dissociation assay and quantification**

This assay was performed as described before (12). In brief, *DMKN* $\beta\gamma^{-/-}$, *DMKN* $\alpha\beta^{-/-}$ or WT keratinocytes were transfected with either siRNA#1, siRNA#2, siRNA#3, vehicle (lipofectamine) scrambled siRNA in T-25 cell culture dish for 72 hours. Cells were detached, counted, and 3e5 cells were placed in 24-well plates for 72 hours. Cells were washed three times with PBS and incubated with 350 µL per well of dispase solution (Sigma-Aldrich) for 120 minutes at 37 ˚C. Afterward, the dispase solution was gently aspirated, 350 µL of PBS per well was added, and cells were gently incubated with 20 µL of 5 µg/µL MTT staining solution for 20 minutes at 37 ˚C. The staining solution was gently aspirated, and 350 µL of PBS was added per well. Dissociated monolayers were pipetted once up-and-down to apply mechanical stress.

To quantify the fragmentation, the plates were imaged using BioRad's ChemiDoc Imaging Systems (white tray, Coomassie Blue settings). The images were analyzed using Qupath (version 0.5.0) and ImageJ as described before(12). Briefly, the entirity of each well was marked by selecting the area within the well, copied into a new file, and the threshold was automatically adjusted. Particles were counted and analyzed via GraphPad Prism (Dotmatics). A two-way ANOVA and Fisher's Least Significant Difference test were used to detect changes among the conditions and illustrated as truncated violin plots.

The same assay was modified for ROCK inhibition. Briefly, cells were incubated with scrambled or p120 siRNA (HSS102464) and incubated for 48 h in a 24-well plate. Subsequently, ROCK inhibitor (10 µM final concentration) (Selleck Chemicals; Y-27632) or DMSO was added for 24 h. The remainder of the assay was performed as before. Data were normalized to DMSO treated values.

**Generation of p120 mutants**

Plasmids were ordered from Genscript using their custom plasmid design software. In brief, the canonical *p120* coding sequence was inserted into the empty pcDNA3.1(+)-C-HA plasmid. To make the construct resistant to the p120 siRNA, a silent point mutation (603 GCT to 603 GCG) was introduced at the siRNA (HSS102464) target site (5`-GCAGCUCCCAAUGUUGCCAACAAUA-3´). To generate the phosphomimetic mutants, serine 252 (AGT) and serine 268 (AGC) were each mutated to GAT, resulting in substitution of aspartic acid at both positions.

**Antibodies**

Antibodies dilutions were optimized for each application and validated for the species on the manufacturer's websites. Antibodies purchased from Cell Signaling Technology: Catenin δ-1 Antibody (#4989) (rabbit polyclonal); Phospho-Catenin δ-1 (Ser252) Antibody (#8477) (rabbit polyclonal); Phospho-Catenin δ-1 (Tyr228) Antibody (#2911) (rabbit polyclonal); purchased from Abcam (Abcam, Cambridge, United Kingdom): Monoclonal Anti-Vinculin antibody produced in Mouse mAb (#V9131); Anti-Integrin alpha 6 antibody [EPR18124] Rabbit mAb (#ab181551); Anti-Cytokeratin 10 antibody [EP1607IHCY] - Cytoskeleton Marker Rabbit mAb (#ab76318); Anti-Cytokeratin 14 antibody [LL002] Mouse mAb (#ab7800); Anti-delta 1 Catenin/CAS (phospho T310) antibody [EPR2382] Rabbit mAb (#ab81318); Anti-DMKN antibody (rabbit polyclonal) (#ab246965); purchased from other suppliers: Phospho-delta Catenin (Ser268) Polyclonal Antibody (rabbit polyclonal) (Thermo Fisher Scientific, #PA5-77935); DMKN Polyclonal antibody (DMKN Polyclonal Antibody for WB, IP, ELISA) (rabbit polyclonal) (Proteintech, #16252-1-AP); alpha Tubulin Antibody (YOL1/34) - BSA Free Rat mAb (Novus Biologicals, #NB100-1639); HA Antibody [12CA5] (mouse monoclonal) (Sigma-Aldrich, #11583816001); gamma Tubulin Antibody (mouse monoclonal) (Sigmal-Aldrich, #T5326); Rabbit anti-IgG-Cy3 (Jackson laboratories; #111-165-003); Goat Anti-Rabbit Ig, Human ads-HRP (Rabbit polyclonal) (Southern biotech, #4010-05); Goat Anti-Mouse IgG, Human ads-HRP (Mouse polyclonal) (Southern biotech, #1030-05); Goat Anti-Rat IgG-HRP (Rat polyclonal) (Southern biotech, #3030-05); Monoclonal Anti-Vinculin antibody produced in Mouse mAb (Sigma-Aldrich, #V9131); Rabbit anti-Ki-67 (Abcam, #16667); Alexa 488 GOAT Anti mouse igG (Invitrogen #A-11001); Alexa 568 Goat Anti rabbit igG (Invitrogen; #A-11011). The anti-dermokine antibody recognizes the last C-terminal 200 amino acids (AA) of dermokine-β(13). The C-terminal 66 AA of dermokine-α are equivalent to the C-terminus of dermokine-β, while dermokine-α has 24 differing AA.

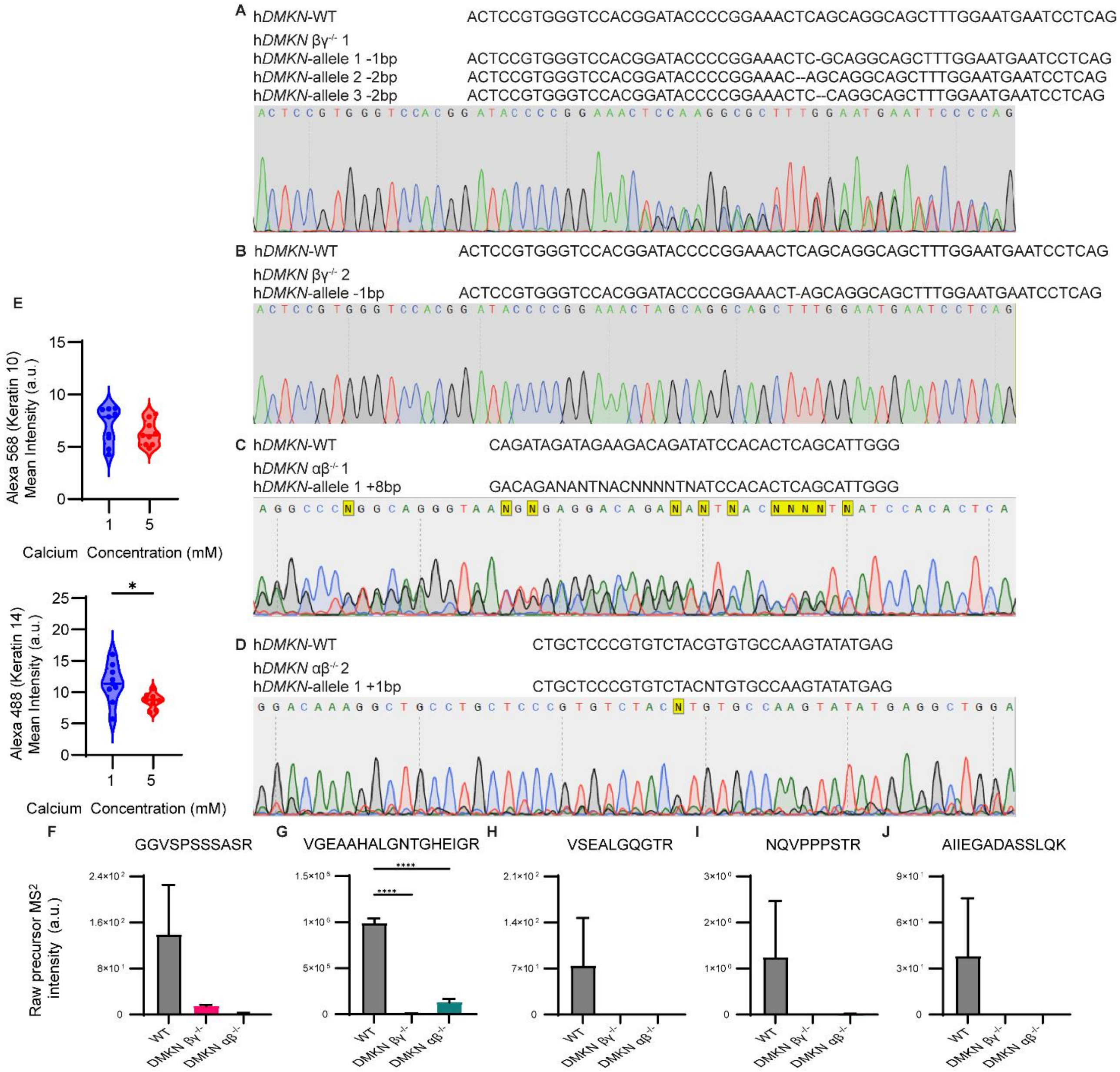


**Supplemental Figure 1. Sanger sequencing validates the ablation of human *DMKN* αβ or *DMKN* βγ from keratinocytes. (A and B)** Results of the Sanger sequencing of the CRISPR/*Cas9* target sequence and the Sanger sequence of the *DMKN* $\beta\gamma^{-/-}$ clone 1 (**A**) or *DMKN* $\beta\gamma^{-/-}$ clone 2 (**B**) keratinocytes. **(C)** Sanger sequences of Chromosome 19 (GRCh 38.p14) region 35500725 to 35500763 are shown for both WT and *DMKN* $\alpha\beta^{-/-}$ keratinocytes clone 1 illustrating indel (insertion/deletion) sites. **(D)** The Sanger sequence of region 35501517 to 35501551 of Chromosome 19 (GRCh 38.p14) are shown for both WT and *DMKN* $\alpha\beta^{-/-}$ keratinocytes clone 12. Both (**C**) and (**D**) show the presence of indels after the excision of exon 18. **(E)** Quantification of immunofluorescent staining of differentiation marker KRT10 and proliferation marker KRT14 in response to keratinocytes incubated with either 1 mM or 5 mM calcium. A.u.: Arbitrary unit. Values are N = 3 different images per condition. * = $P < 0.05$, (unpaired t-test) **(F-J)** Targeted proteomics analysis of proteotypic dermokine peptides, values are raw $MS^2$ precursor intensities. A.u.: Arbitrary unit. Values are N = 3 biological

3D organotypic skin culture replicates. **** = P < 0.0001 (one-way ANOVA and a Fishers Least Significant Difference test).

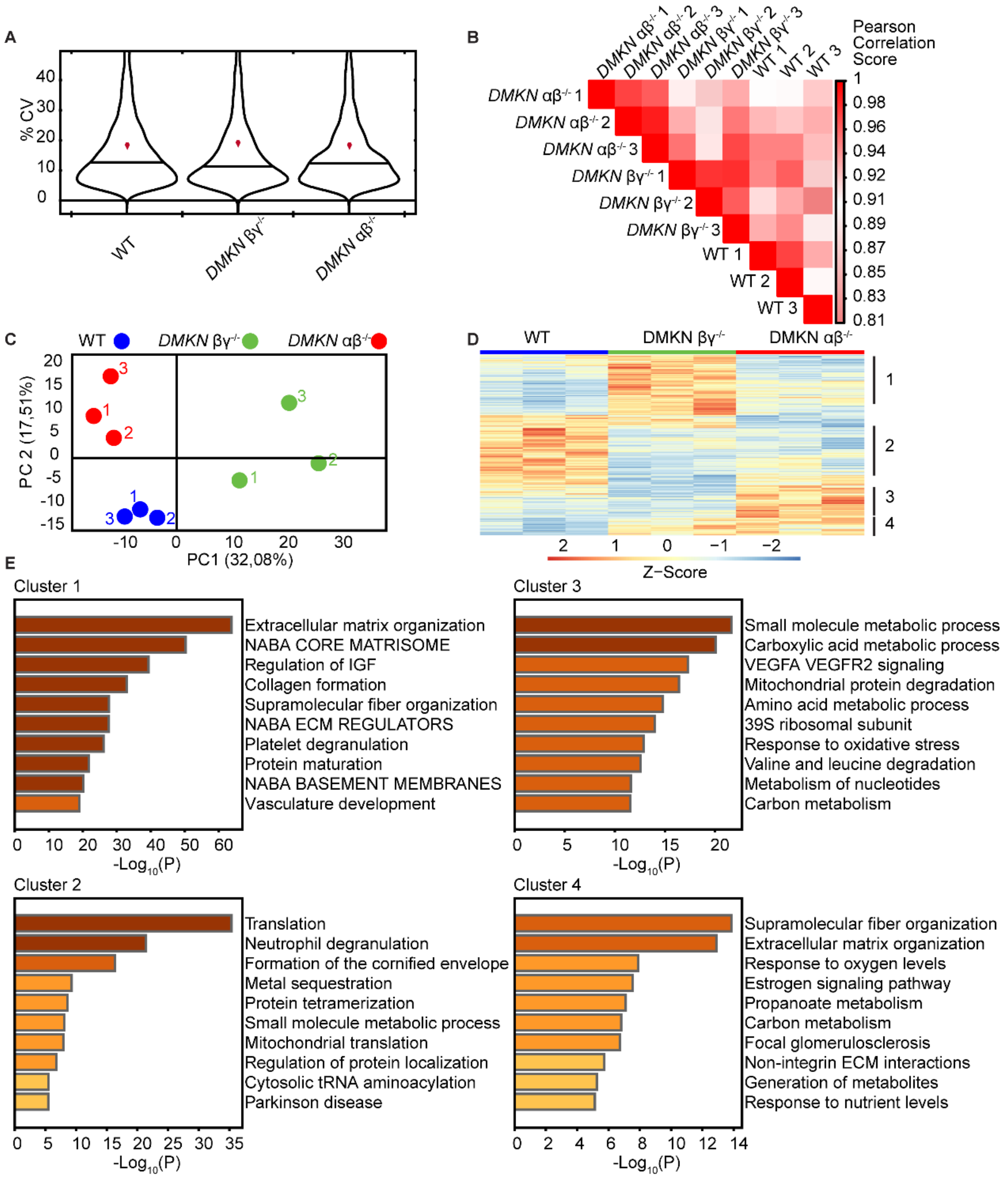


**Supplemental Figure 2: Proteomic analysis of the WT and DMKN KO 3D organotypic skin cultures demonstrates high data quality. (A)** Coefficient of variation (CV) of the WT, *DMKN* αβ-/- and *DMKN* βγ-/- 3D organotypic skin culture proteomes. **(B)** Pearson **c**orrelation analysis of WT, *DMKN* αβ-/- or *DMKN* βγ-/- 3D organotypic skin cultures proteomes. **(C)** Principal component analysis (PCA) of the indicated data sets. **(D)** Differences in the proteomes of *DMKN* αβ-/-, *DMKN* βγ-/- or WT 3D organotypic skin cultures visualized as heatmap, and four clusters numbered 1 to 4 on the right. **(E)** Gene Ontology enrichment analysis of the different *DMKN* KO proteome clusters shown in **(D)**.

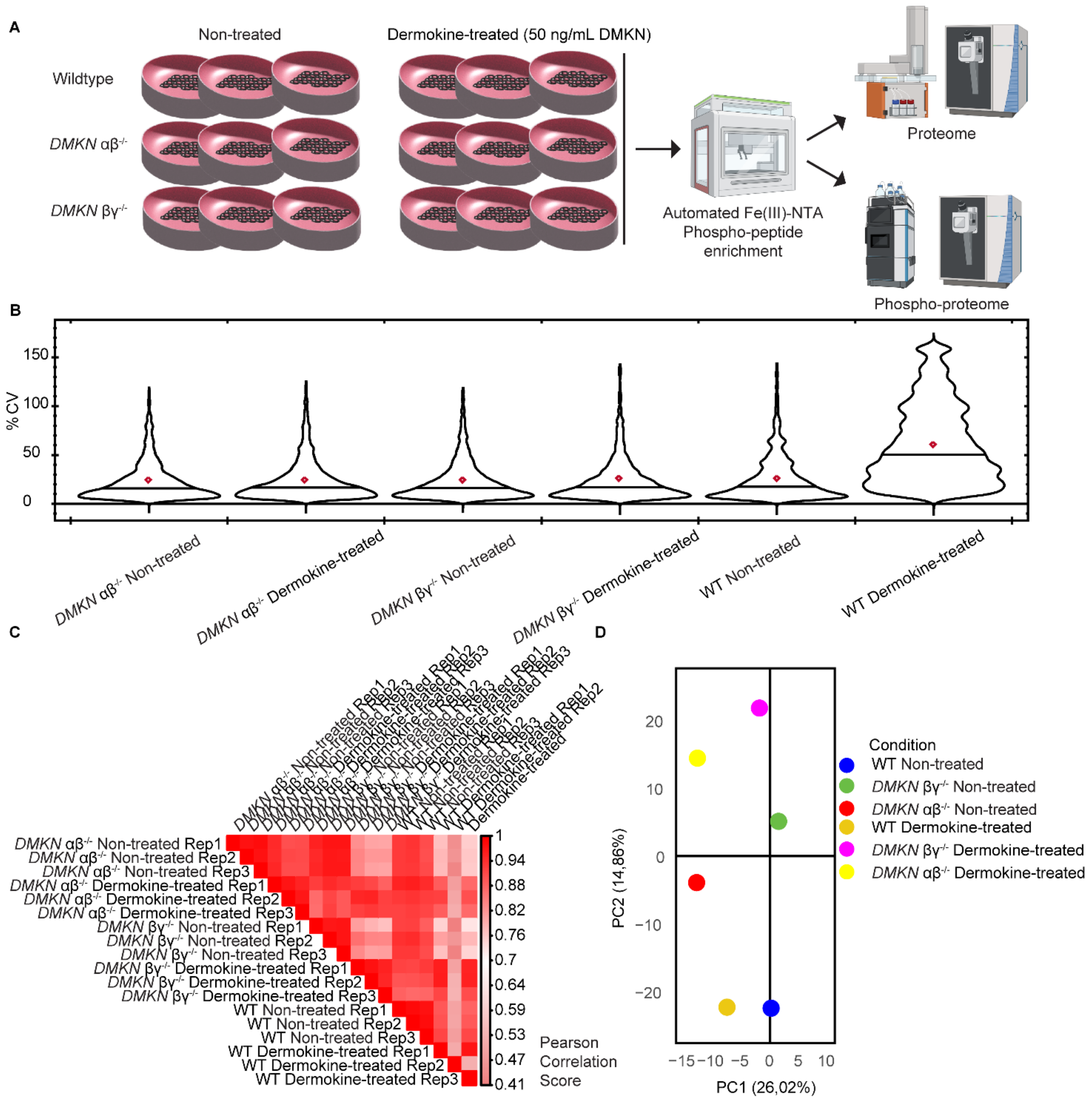


**Supplemental Figure 3: Supplemental evaluation of proteomics analysis of WT, *DMKN* αβ$^{-/-}$ and *DMKN* βγ$^{-/-}$ keratinocytes detects changes. (A)** Overview of the experimental setup which includes non-treated *DMKN* αβ$^{-/-}$ or *DMKN* βγ$^{-/-}$ and WT keratinocytes together with dermokine-treated *DMKN* αβ$^{-/-}$ or *DMKN* βγ$^{-/-}$ and WT keratinocytes. The data was acquired on an Exploris Orbitrap with a 140-minute gradient in an optimized HRMS$^1$-DIA mode. **(B)** Coefficient of variation (CV) of non-treated and dermokine-treated *DMKN* αβ$^{-/-}$ or *DMKN* βγ$^{-/-}$ and WT keratinocytes. **(C)** Correlation analysis of either non-treated or dermokine-treated WT, *DMKN* αβ$^{-/-}$ or *DMKN* βγ$^{-/-}$ proteomes. **(D)** PCA of the median of each replicate within the indicated data sets.

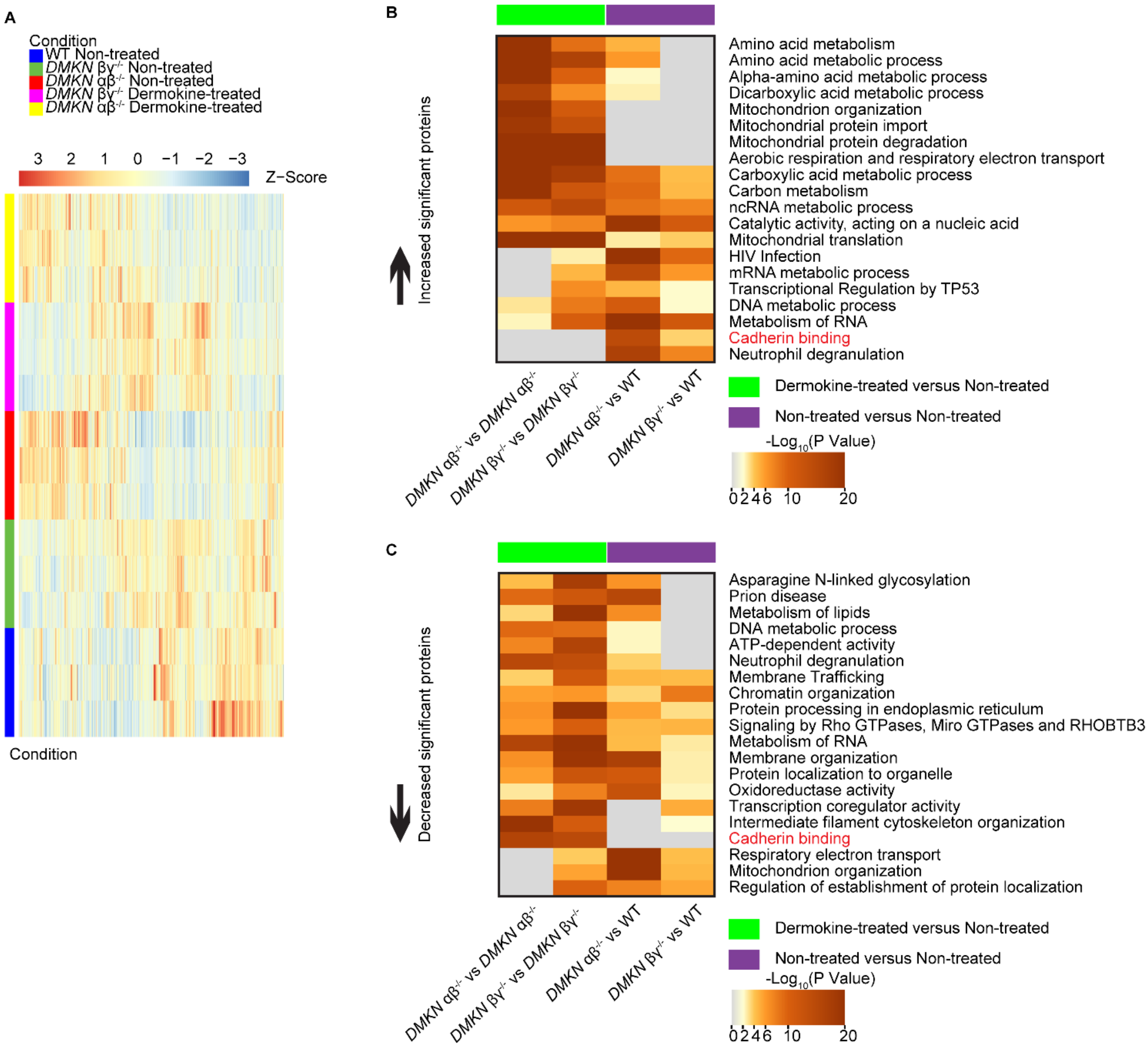


**Supplemental Figure 4: Pathway analysis of WT, *DMKN* αβ$^{-/-}$ and *DMKN* βγ$^{-/-}$ reveals changes in the proteome. (A)** Scaled proteome analysis of either *DMKN* αβ$^{-/-}$ and *DMKN* βγ$^{-/-}$ keratinocytes treated with recombinant dermokine or vehicle and of knockout *vs.* WT keratinocyte proteomes. **(B and C)** Gene ontology analysis of the *DMKN* βγ$^{-/-}$, *DMKN* αβ$^{-/-}$ and WT keratinocyte proteome reveals pathways that are significantly increased (**B**) or decreased (**C**) in *DMKN* βγ$^{-/-}$ or *DMKN* αβ$^{-/-}$ keratinocytes after treatment with recombinant dermokine.

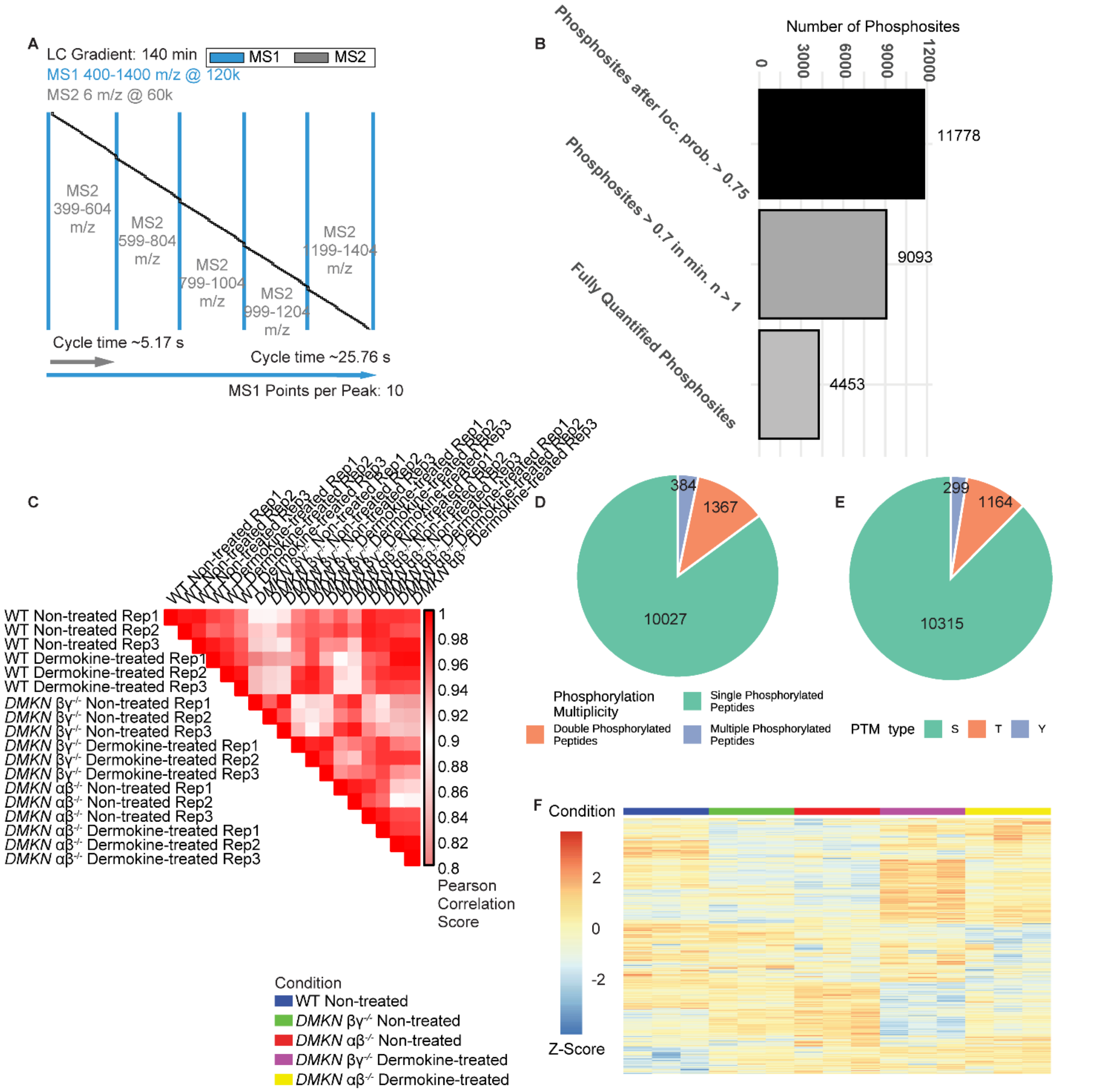


**Supplemental Figure 5: Quality assessment of the WT, *DMKN* αβ$^{-/-}$ and *DMKN* βγ$^{-/-}$ keratinocyte phosphoproteome dataset. (A)** Workflow of the method used to acquire the phosphoproteome. **(B)** Total number of acquired phosphorylated sites after applying the site localization filter of 0.75 and excluding non-fully quantified phosphorylated sites. **(C)** Pearson correlation analysis of WT, *DMKN* αβ$^{-/-}$ or *DMKN* βγ$^{-/-}$ proteomes. **(D)** Distribution of singly doubly or multiple phosphorylated peptides. **(E)** Distribution of phosphorylated serine, threonine and tyrosine sites. **(F)** Scaled phosphoproteome intensities showing differences between conditions.

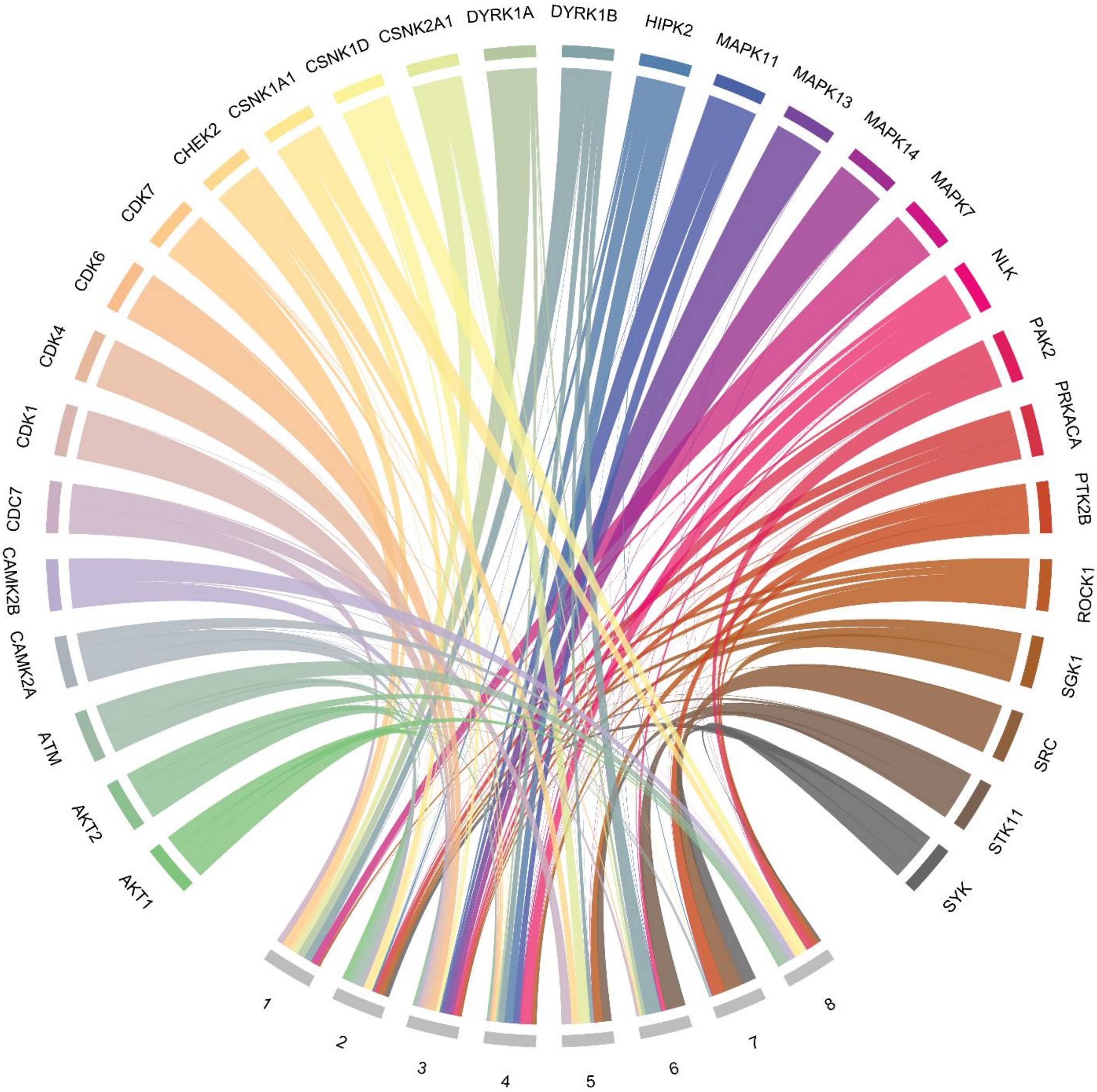


**Supplemental Figure 6: Clustering of phosphorylated proteins based on kinase preference shows phosphorylated site modules.** Circular signalome map clustering phosphorylated sites according to kinase preference. The calculated kinase-substrate scoring of phosphorylated sites demonstrate that sites cluster into phosphorylation modules. Cluster 5 and 7 contain phosphorylated tyrosine and threonine sites. Module 7 includes PTK2B, SRC and SYK activity.

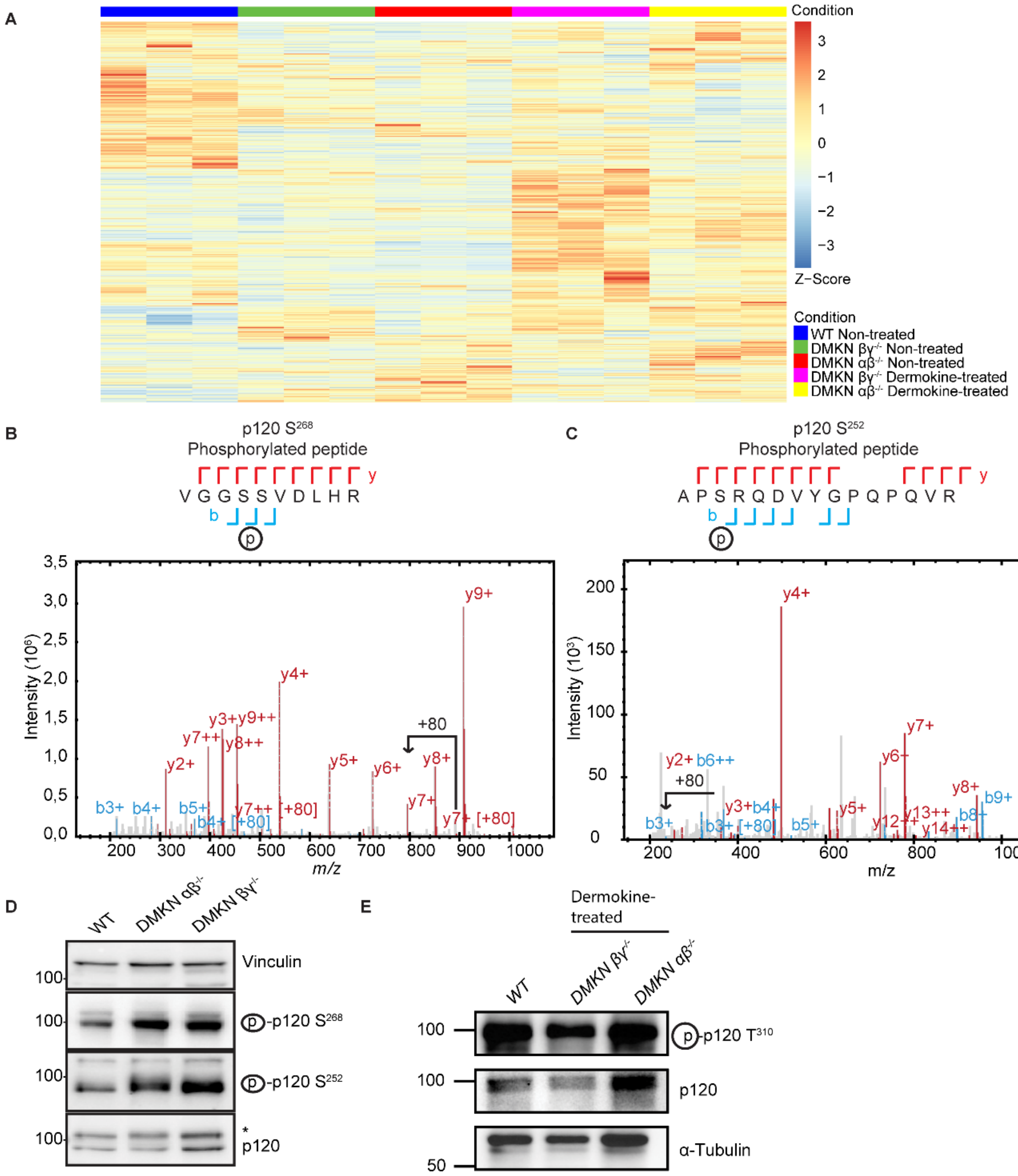


**Supplemental Figure 7: WT, *DMKN* $\alpha\beta^{-/-}$, and *DMKN* $\beta\gamma^{-/-}$ keratinocyte phosphoproteomes show changes in p120 phosphorylation. (A)** All phosphorylated proteins were normalized to their protein expression and scaled, demonstrating differences between conditions. **(B and C)** MS/MS spectra of the tryptic phosphorylated peptide VGGpSSVDLHR from p120 at $S^{268}$ **(B)** and APpSRQDDVYGPSQPQVR from p120 at $S^{252}$ **(C)**. **(D)** Immunoblotting with the indicated antibodies of p120 phosphorylation ($S^{252}$ and $S^{268}$) in human *DMKN* $\alpha\beta^{-/-}$, *DMKN* $\beta\gamma^{-/-}$ and WT keratinocytes. **(E)** Immunoblotting with indicated antibodies of p120 phosphorylation $T^{310}$ in WT and supplemented *DMKN* $\alpha\beta^{-/-}$, *DMKN* $\beta\gamma^{-/-}$ keratinocytes.

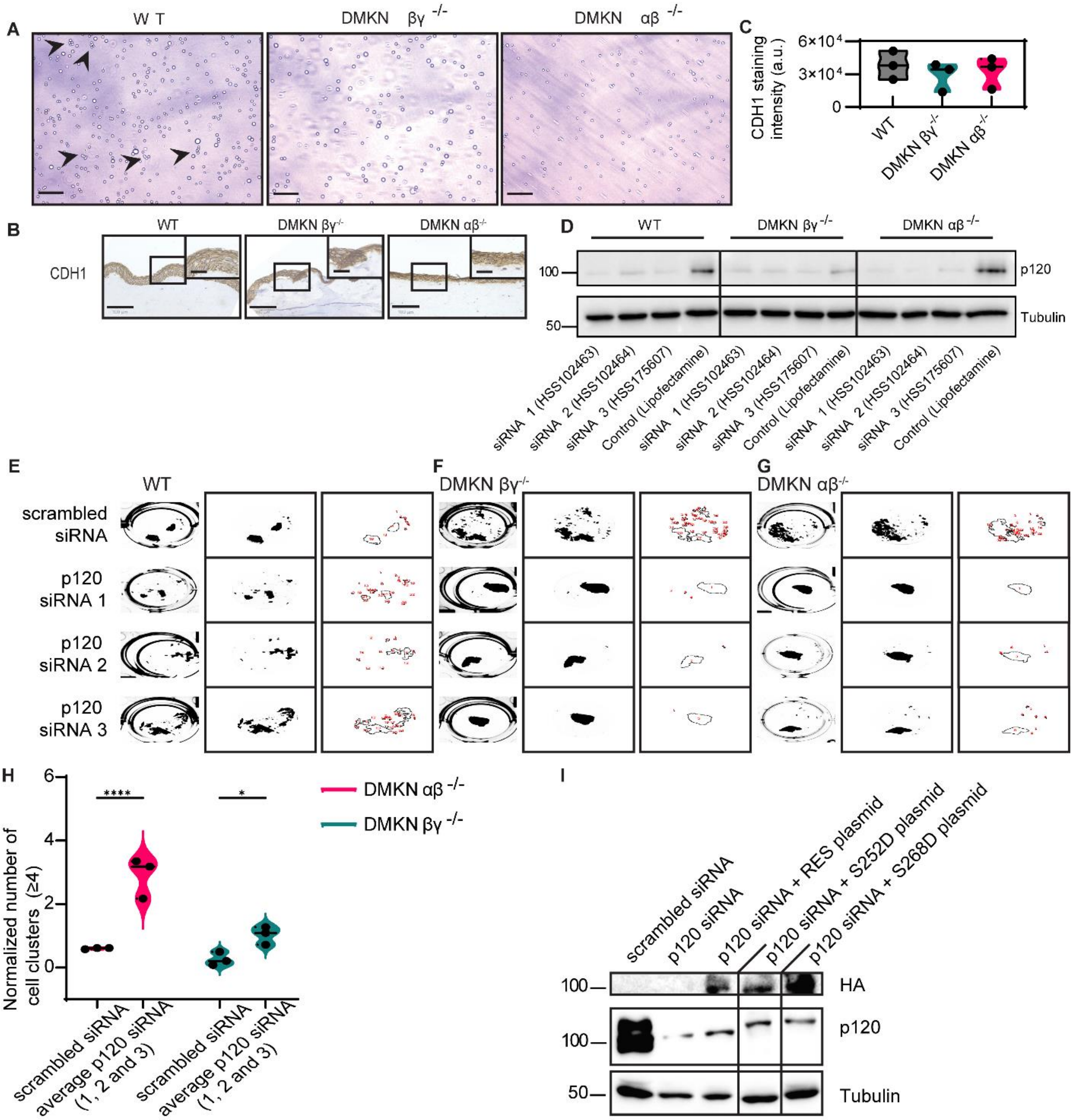


**Supplemental Figure 8: Dermokine mediates cell-cell adhesion. (A)** Representative images of the cell clusters from the cell-cell adhesion experiment quantified in Figure 5A. Arrows indicate the presence of four or more cell clustering together. Scale bar = 200 μm. **(B and C)** Representative images (**B**) and quantification (**C**) of human *DMKN* $\alpha\beta^{-/-}$, *DMKN* $\beta\gamma^{-/-}$ and WT 3D organotypic skin cultures stained with anti-cadherin-1. N = 3 different 3D organotypic skin cultures, scale bar = 100 μm and scale bar (magnified image) = 50 μm. One-way ANOVA and a Fishers Least Significant Difference test. A.u.: Arbitrary unit. **(D)** Immunoblotting with the indicated antibodies of lysates from *DMKN* $\alpha\beta^{-/-}$, *DMKN* $\beta\gamma^{-/-}$ and WT keratinocytes incubated for 72 hours with p120 siRNA 1, 2 and 3 and a control (lipofectamine). **(E, F and G)** Representative images from the dispase dissociation assay showing cell culture dishes (left), unsegmented cell fragments (middle), and segmented cells (right) for WT (**D**), *DMKN* $\beta\gamma^{-/-}$ (**E**), and *DMKN* $\alpha\beta^{-/-}$ (**F**) keratinocytes. Cells were transfected for 72 h with

either scrambled siRNA or one of three p120 siRNAs (siRNA 1, 2 or 3). **(H)** *DMKN* $αβ^{-/-}$, *DMKN* $βγ^{-/-}$ and WT keratinocytes transfected for 72 h with p120 siRNA (1, 2 and 3) as well as scrambled siRNA were subjected to the cell-cell adhesion assay. The number of cell cluster (≥4) of *DMKN* $αβ^{-/-}$ and *DMKN* $βγ^{-/-}$ keratinocyte were counted and normalized to the average WT number of cell cluster within the same siRNA treatment. Shown are the siRNA averages for each genotype. N = 3 biological replicates. * = P < 0.05, **** = P < 0.0001 (two-way ANOVA and a Fishers Least Significant Difference test) **(I)** Immunoblotting with the indicated antibodies of lysates from *DMKN* $αβ^{-/-}$, *DMKN* $βγ^{-/-}$ and WT keratinocytes incubated for 72 hours with scrambled siRNA, p120 siRNA, p120 siRNA and p120 resistant plasmid, p120 siRNA and p120 resistant plasmid that included the S252D or the S268D mutation. Irrelevant conditions were removed. Supplementary data shows the uncropped immunoblots. N=3 biological replicates.

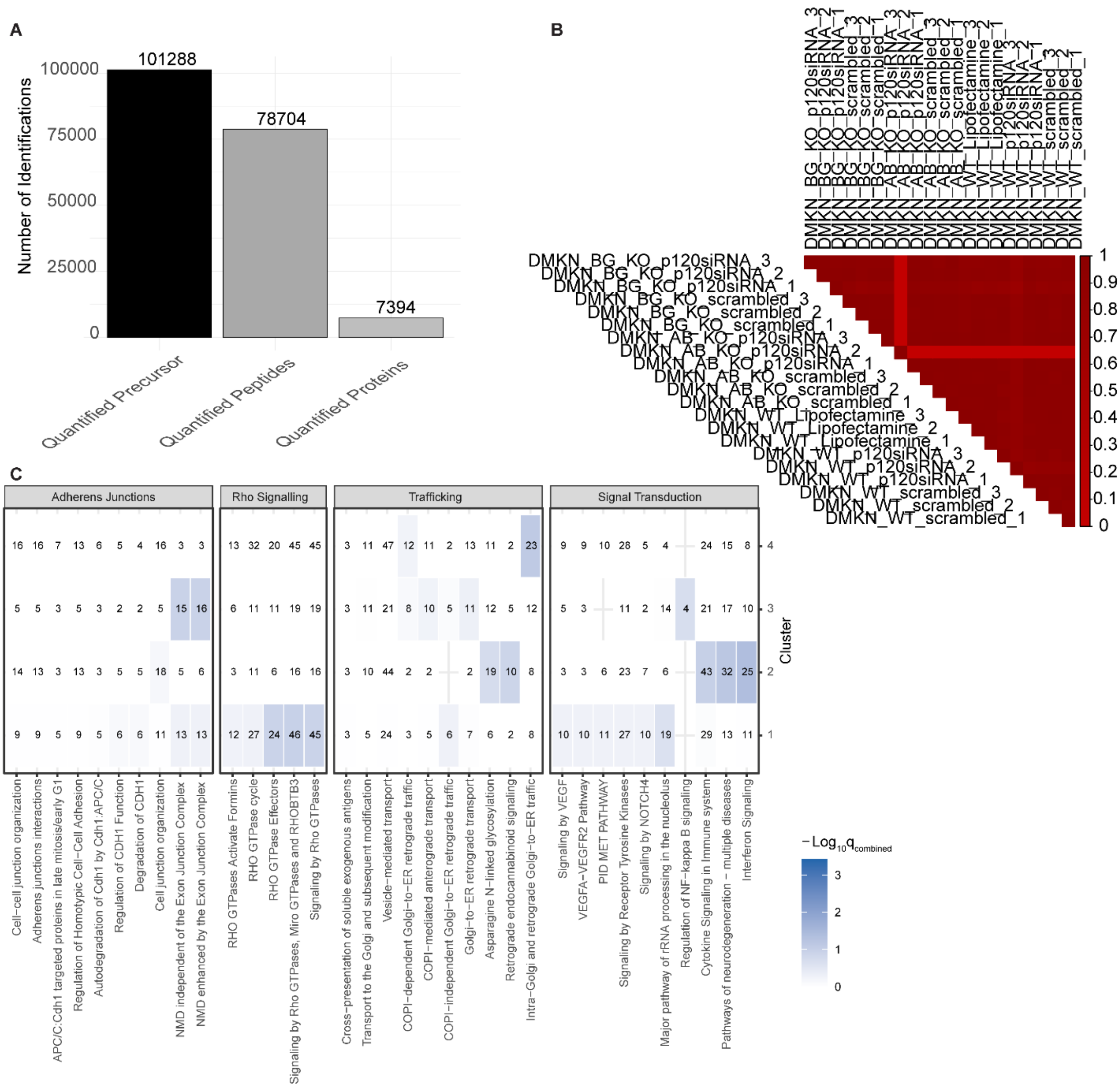


**Supplemental Figure 9: Quality assessment of the proteome collected upon perturbation or not of p120 in WT and KO cells. (A)** Total number of acquired precursors, peptides and proteins. **(B)** Pearson correlation analysis of WT, *DMKN* αβ$^{-/-}$ or *DMKN* βγ$^{-/-}$ proteomes. **(C)** Pathway enrichment of cluster 1, 2, 3 and 4 depicted in Figure 6A. The color scale ($-Log_{10}(q_{combined})$) represents the aggregated and multiple-testing corrected significance of the module in each cluster. Filled values are number of overlapping protein names in that term and cluster.

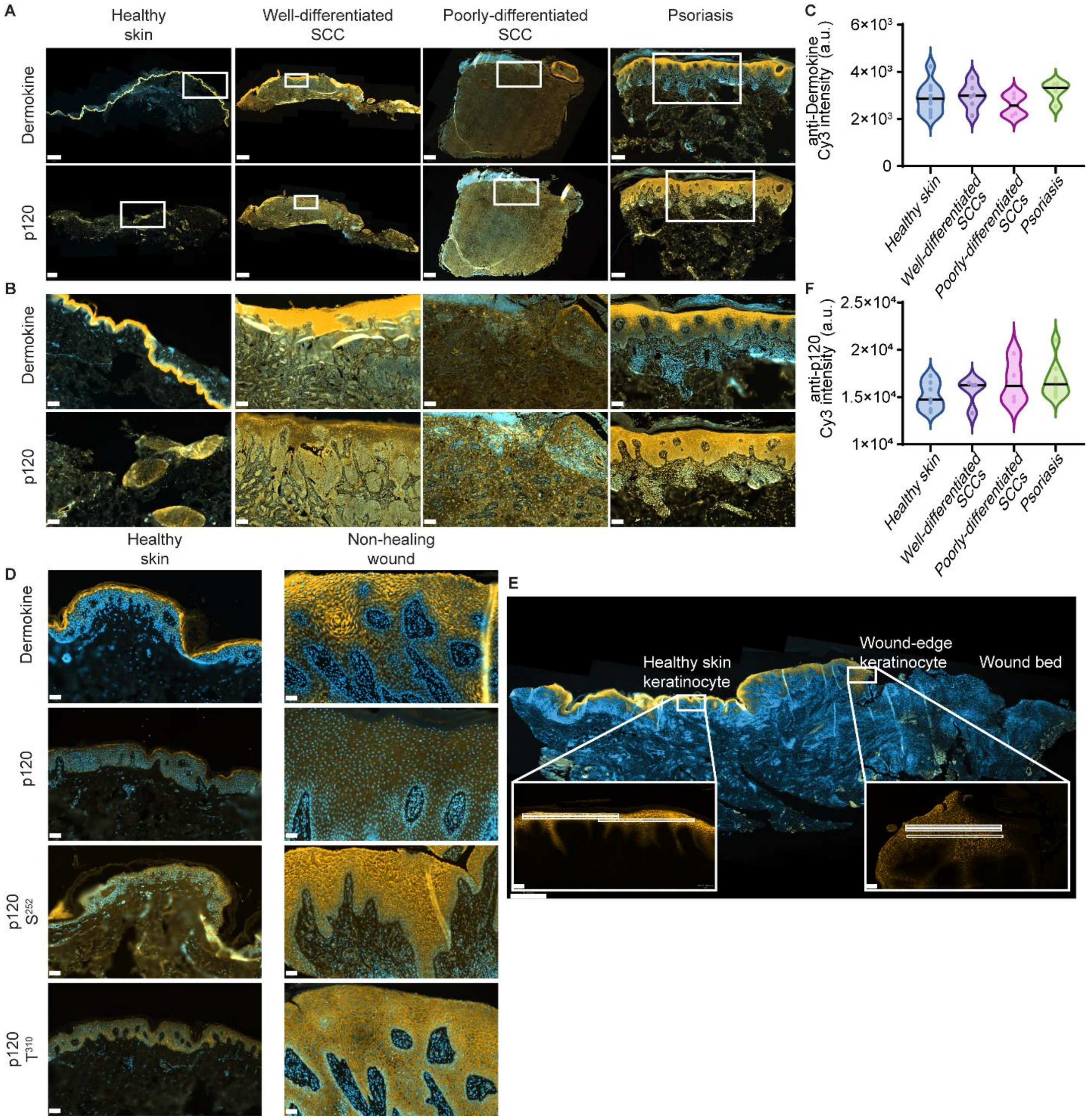


**Supplemental Figure 10: Immunofluorescence staining of healthy and psoriatic skin and non-differentiated squamous cell carcinoma. (A)** Immunofluorescence images of patient tissue of healthy skin, well- and poorly- differentiated SCCs and psoriatic skin with the indicated antibodies. The scale bar for the anti-dermokine healthy skin image is 800 µm and for the anti-p120 image 500 µm. The scale bar for the well-differentiated SCC images is 1 mm, 500 µm for the poorly-differentiated SCC images and 250 µm for the images of psoriatic skin. **(B)** Magnified images from the squared area indicated in (**A**). The scale bar for the magnified images is 100 µm. **(C and F)** Quantification of (**A**). Values are either raw Cy3 intensities (anti-dermokine (**C**)) or -p120 (**F**) of samples from at least N = 3 different patients **(D)** Immunofluorescence images of patient tissue of healthy skin and non-healing wounds with the indicated antibodies from the squared insert shown in Figure 7A). The scale bar is 50 µm. **(E)** Immunofluorescence staining of a non-healing wound bed

(sample HS17.88) from a venous leg ulcer and the surrounding healthy skin. Scalebar = 1 mm (full image) and 100 µm (detailed view), respectively.

**Supplemental table legends:**

**Supplemental table 1**: Shows dermokine proteotypic peptides and their fragment ions (both isotopic and endogenous) in WT, *DMKN* $\alpha\beta^{-/-}$ and *DMKN* $\beta\gamma^{-/-}$ 3D organotypic skin cultures.

**Supplemental table 2**: Shows LC-MS-based proteomics quantification of WT, *DMKN* $\alpha\beta^{-/-}$ and *DMKN* $\beta\gamma^{-/-}$ 3D organotypic skin cultures.

**Supplemental table 3**: Shows LC-MS-based proteomics quantification of non-treated and dermokine-treated WT, *DMKN* $\alpha\beta^{-/-}$ and *DMKN* $\beta\gamma^{-/-}$ keratinocytes.

**Supplemental table 4**: Shows quantified phosphorylated sites of non-treated and dermokine-treated WT, *DMKN* $\alpha\beta^{-/-}$ and *DMKN* $\beta\gamma^{-/-}$ keratinocytes.

**Supplemental table 5**: Shows the combined score of phosphorylated sites, computed via kinase-substrate prediction from non-treated and dermokine-treated WT, *DMKN* $\alpha\beta^{-/-}$ and *DMKN* $\beta\gamma^{-/-}$ keratinocytes. Shows the kinase activity score of 94 kinases, computed via kinase-substrate prediction from endogenous and supplemented WT, *DMKN* $\alpha\beta^{-/-}$ and *DMKN* $\beta\gamma^{-/-}$ keratinocytes. Shows the 8 cluster of phosphorylated sites sharing similar kinase profiles and regulations. Shows kinases associated with each phosphorylated site and how many sites each phosphosite displayed across all modules in the pathways associated with cadherin binding and adherens junctions.

**Supplemental table 6**: Shows all quantified proteins from p120 perturbed and unperturbed WT, *DMKN* $\alpha\beta^{-/-}$ and *DMKN* $\alpha\beta^{-/-}$ keratinocytes.

**Supplemental table 7**: Shows all proteins from Cluster 1, 2, 3 and 4.

**Supplemental table 8**: Shows Gene Ontology analysis results for proteins from Cluster 1, 2, 3 and 4.

**Supplemental table 9**: Shows human donor information.

**Supplemental references:**


1. Wickham H. *ggplot2: Elegant Graphics for Data Analysis*. Springer-Verlag New York; 2016.

2. Zhou Y, et al. Metascape provides a biologist-oriented resource for the analysis of systems-level datasets. *Nat Commun*. 2019;10(1).

3. MacLean B, et al. Skyline: an open source document editor for creating and analyzing targeted proteomics experiments. *Bioinformatics*. 2010;26(7):966–968.

4. Gessulat S, et al. Prosit: proteome-wide prediction of peptide tandem mass spectra by deep learning. *Nat Methods*. 2019;16(6):509–518.

5. auf dem Keller U, et al. A statistics-based platform for quantitative N-terminome analysis and identification of protease cleavage products. *Mol Cell Proteomics*. 2010;9(5):912–927.

6. Watson J, et al. Spatially resolved phosphoproteomics reveals fibroblast growth factor receptor recycling-driven regulation of autophagy and survival. *Nat Commun*. 2022;13(1):6589.

7. Casado P, et al. Kinase-substrate enrichment analysis provides insights into the heterogeneity of signaling pathway activation in leukemia cells. *Sci Signal*. 2013;6(268):rs6.

8. Kim HJ, et al. PhosR enables processing and functional analysis of phosphoproteomic data. *Cell Rep*. 2021;34(8).

9. Kauko O, et al. Label-free quantitative phosphoproteomics with novel pairwise abundance normalization reveals synergistic RAS and CIP2A signaling. *Scientific Reports 2015 5:1*. 2015;5(1):1–17.

10. Dejana E, Orsenigo F, Lampugnani MG. The role of adherens junctions and VE-cadherin in the control of vascular permeability. *J Cell Sci*. 2008;121(13):2115–2122.

11. Candi E, Schmidt R, Melino G. The cornified envelope: a model of cell death in the skin. *Nature Reviews Molecular Cell Biology 2005 6:4*. 2005;6(4):328–340.

12. Schmidt MF, et al. Pitfalls in the Application of Dispase-Based Keratinocyte Dissociation Assay for In Vitro Analysis of Pemphigus Vulgaris. *Vaccines (Basel)*. 2022;10(2).

13. Uhlén M, et al. Tissue-based map of the human proteome. *Science (1979)*. 2015;347(6220).
