# Supplementary material for: Multiomics analysis reveals dermokine as a regulator of keratinocyte differentiation and adhesion
Source: JCI Insight. 2026 Apr 23;11(13):e197838. doi: 10.1172/jci.insight.197838 (PMC13460826; doi:10.1172/jci.insight.197838)

Full unedited blot/gel for Supplemental Figure 7D

p120 S268

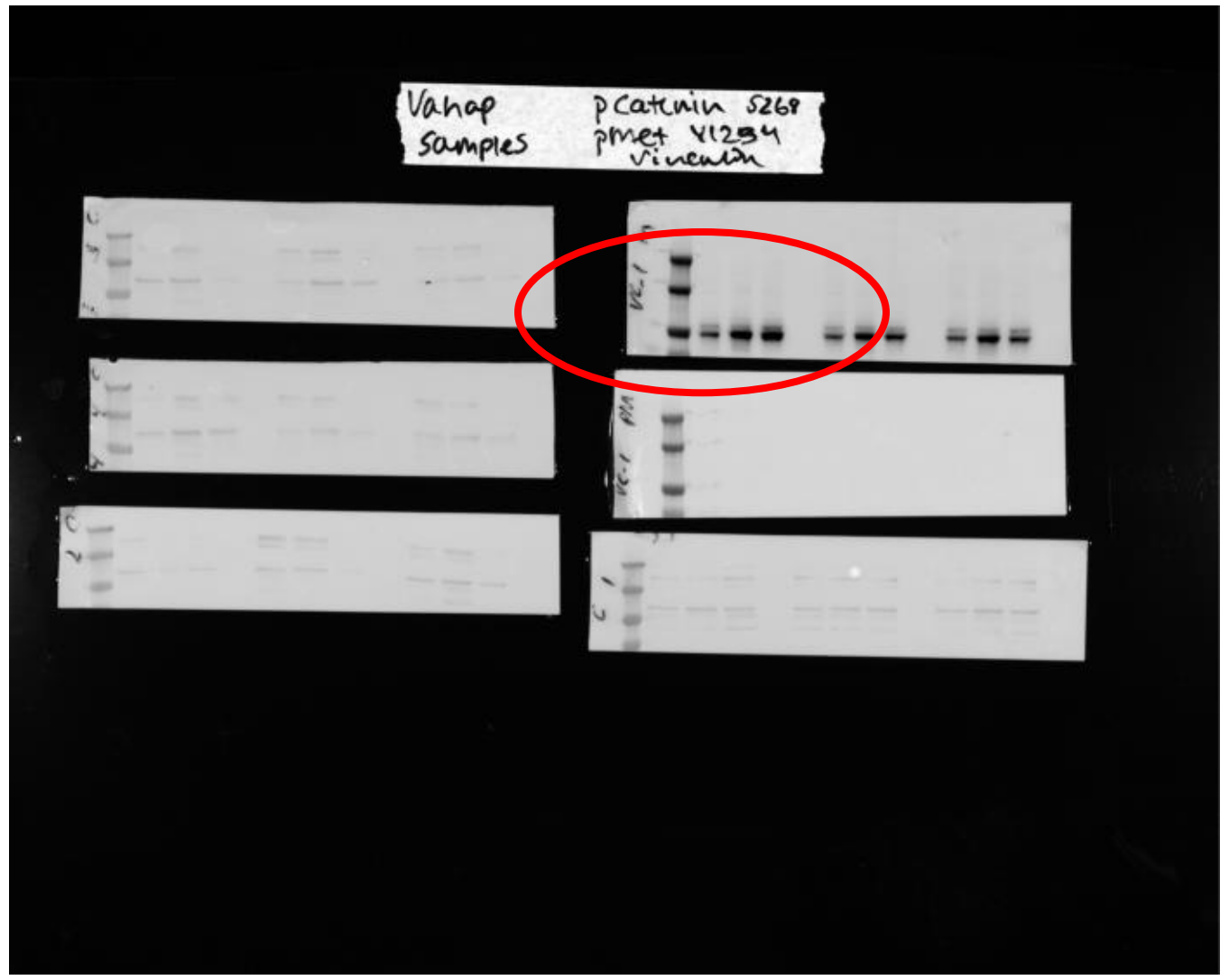

p120 S252

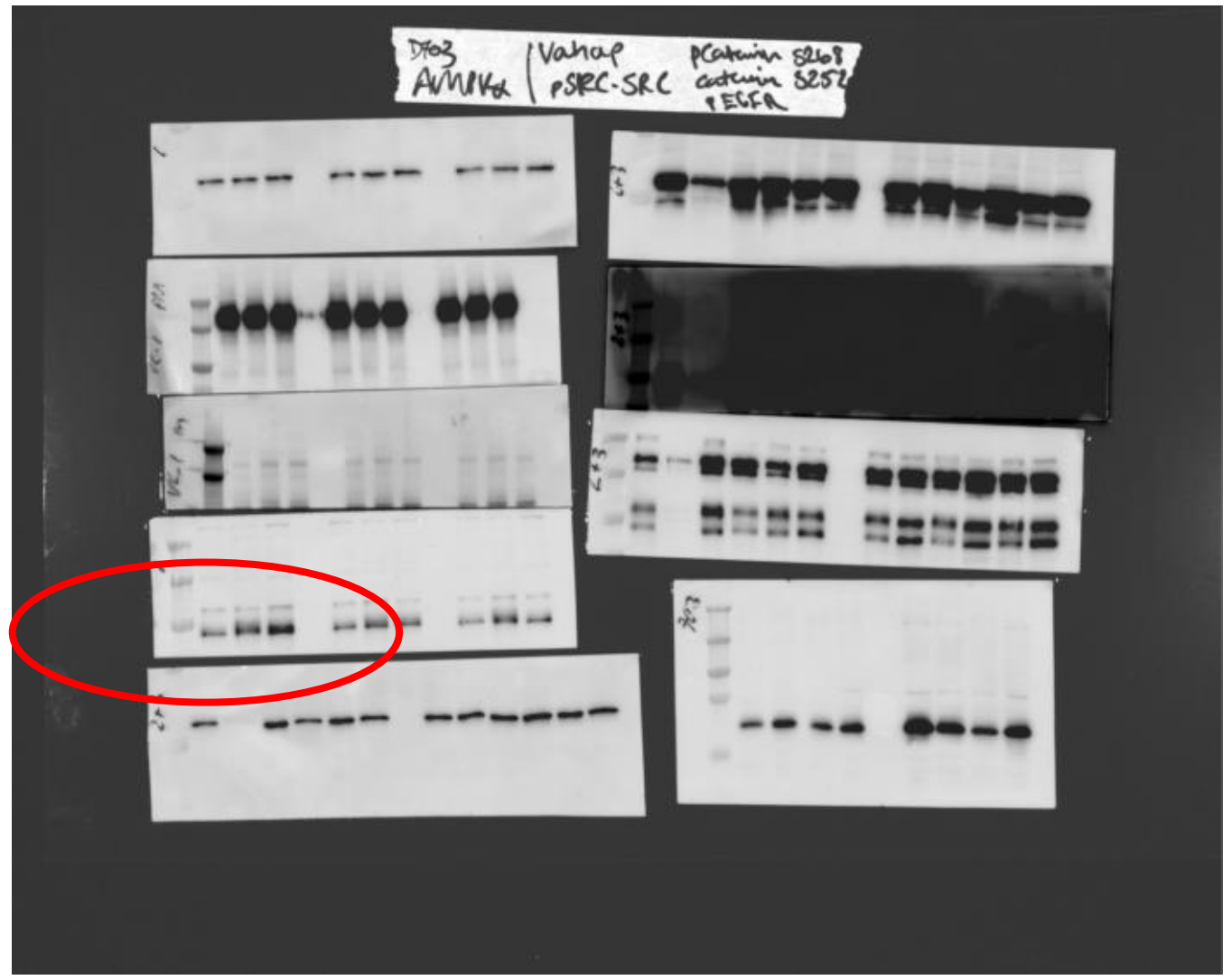

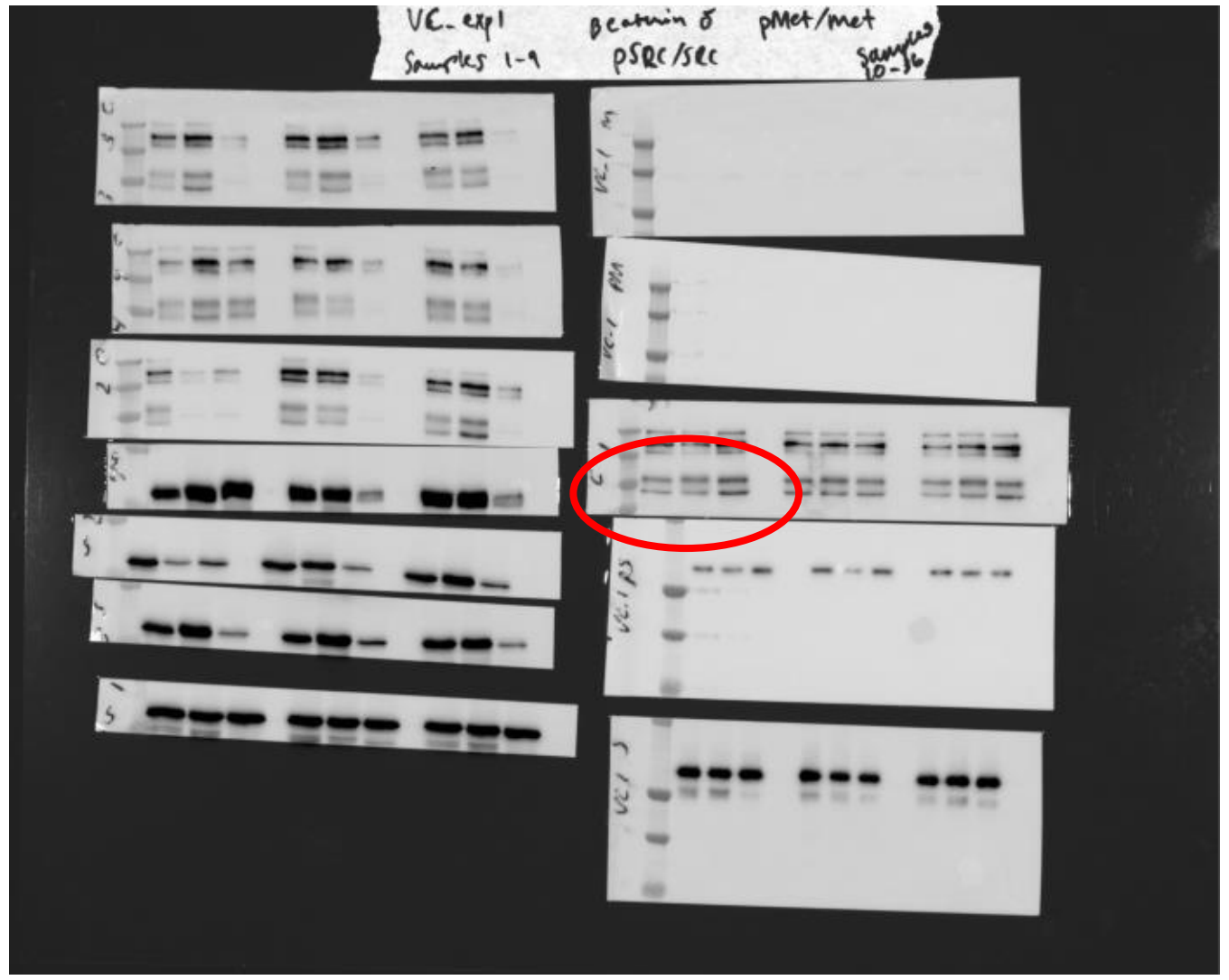

Total p120

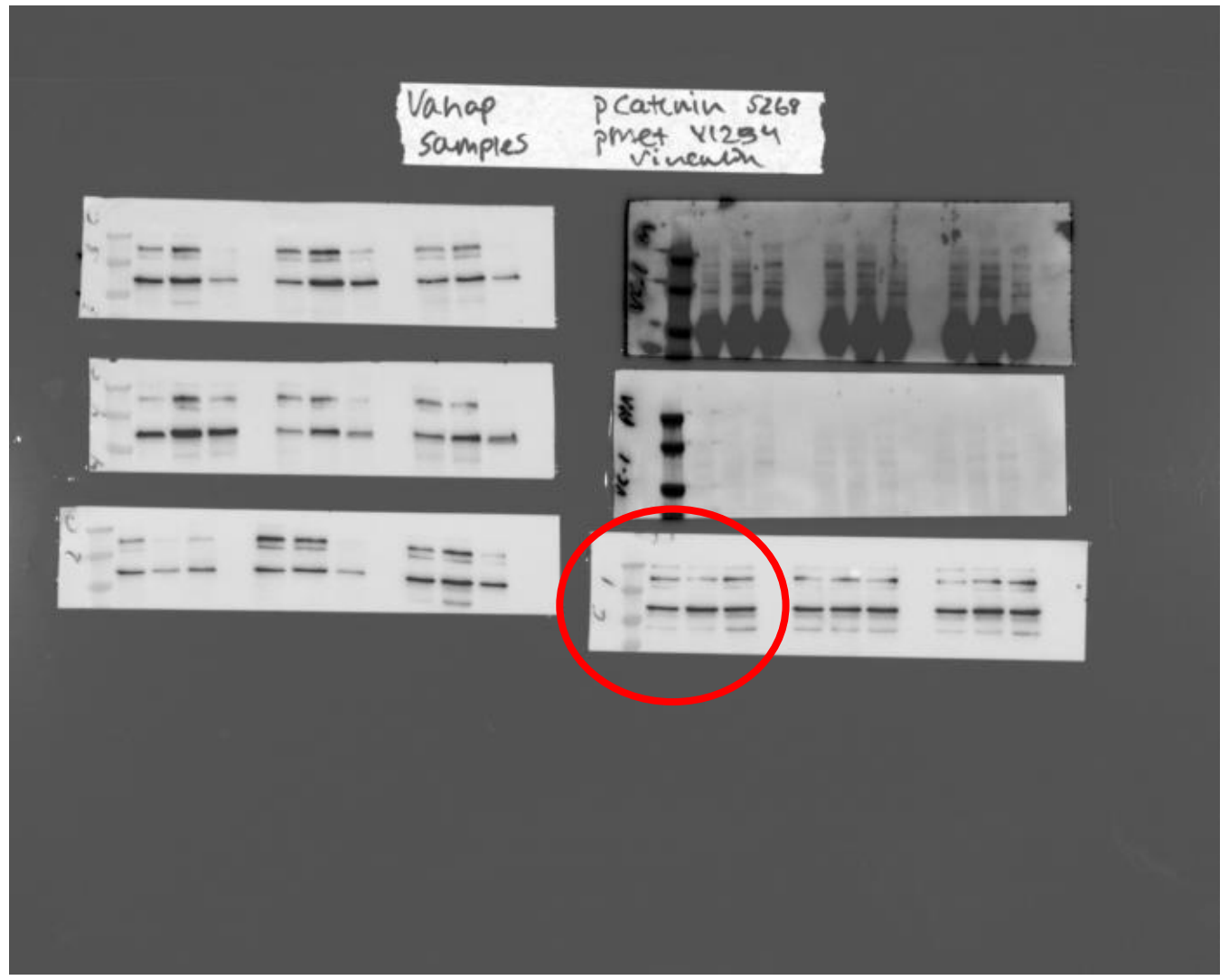

VCL

Full unedited blot/gel for Supplemental Figure 7E

α–Tubulin

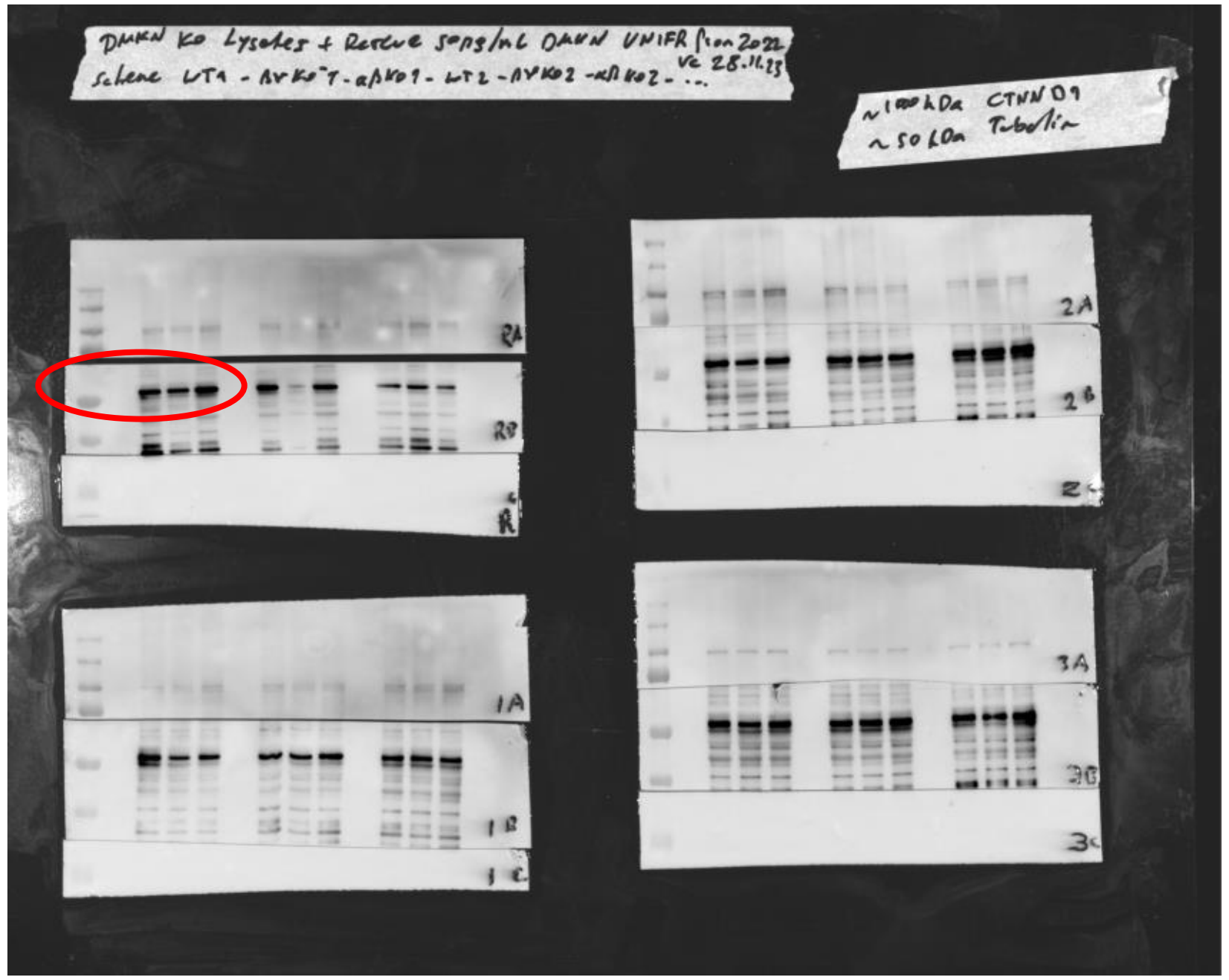

p120 T310

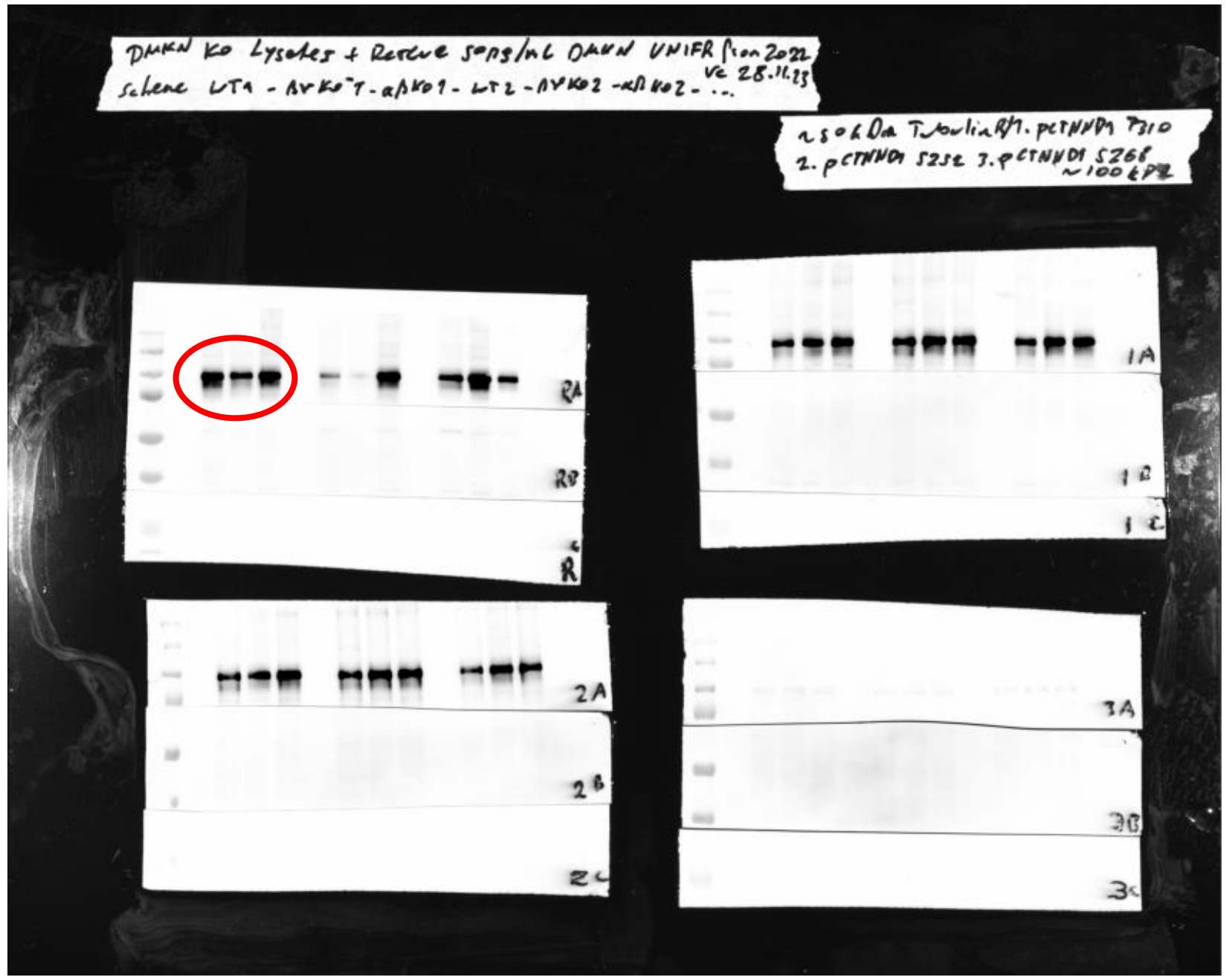

Total p120

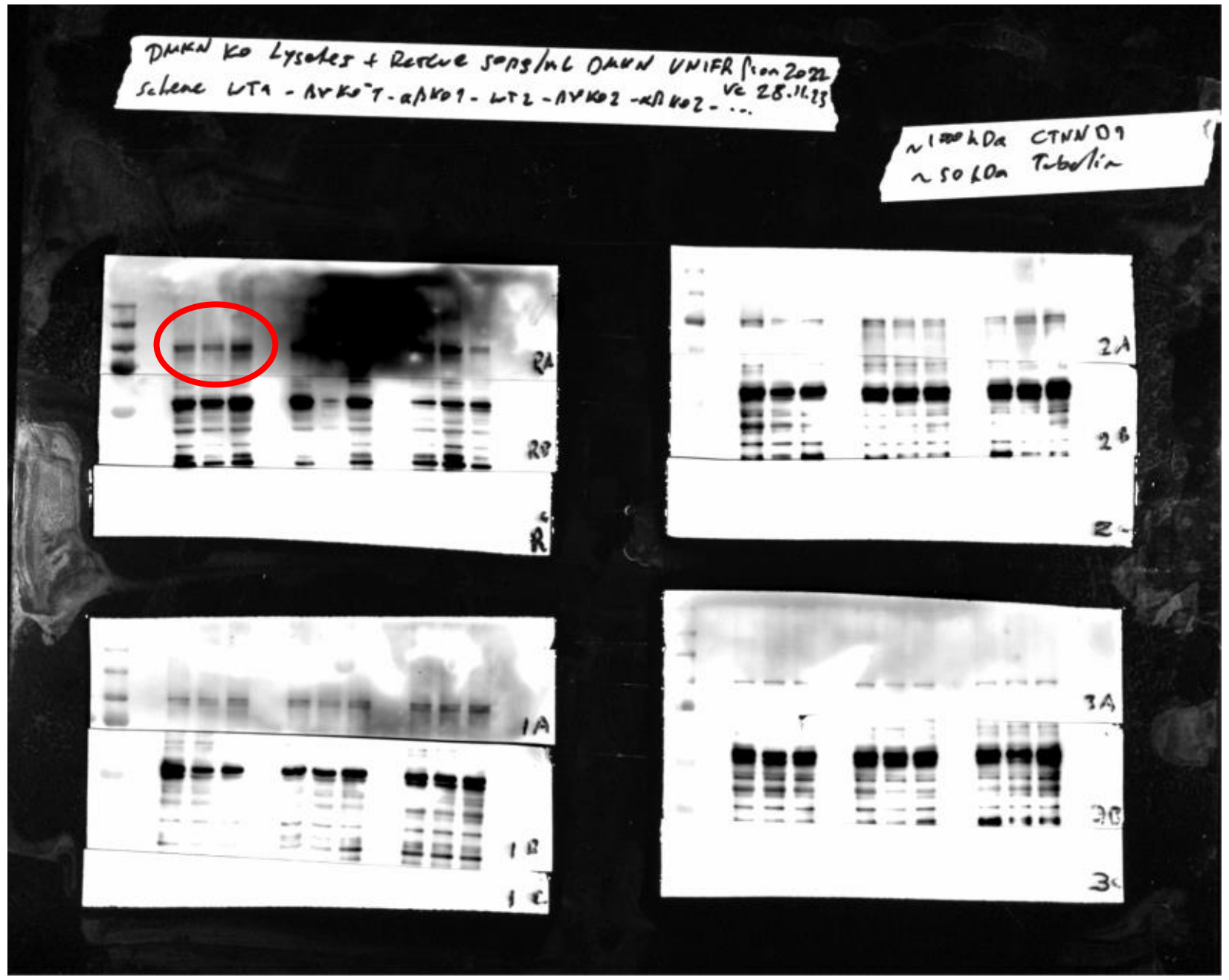

Full unedited blot/gel for Supplemental Figure 8D

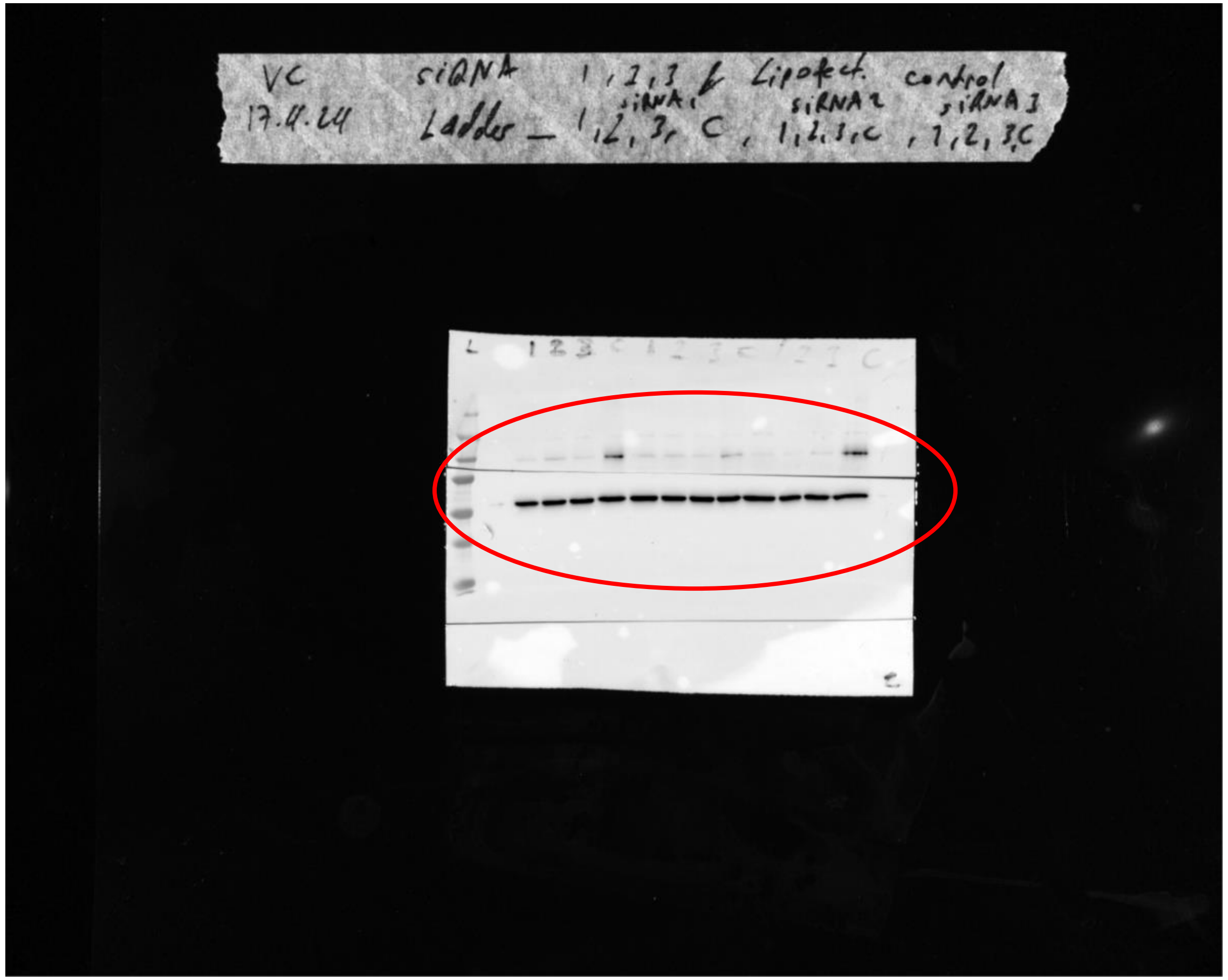

Full unedited blot/gel for Supplemental Figure 8I

p120

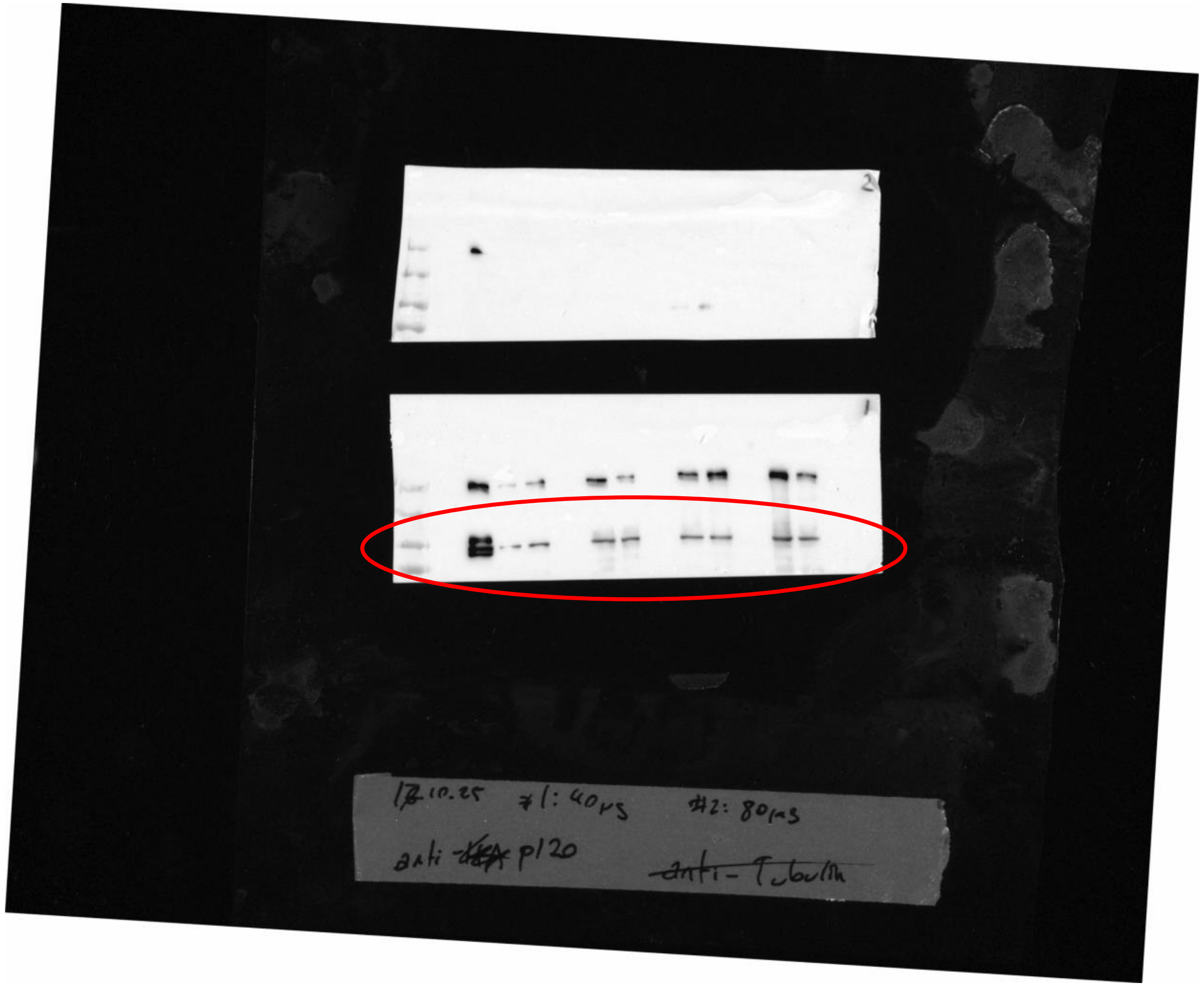

HA

α–Tubulin

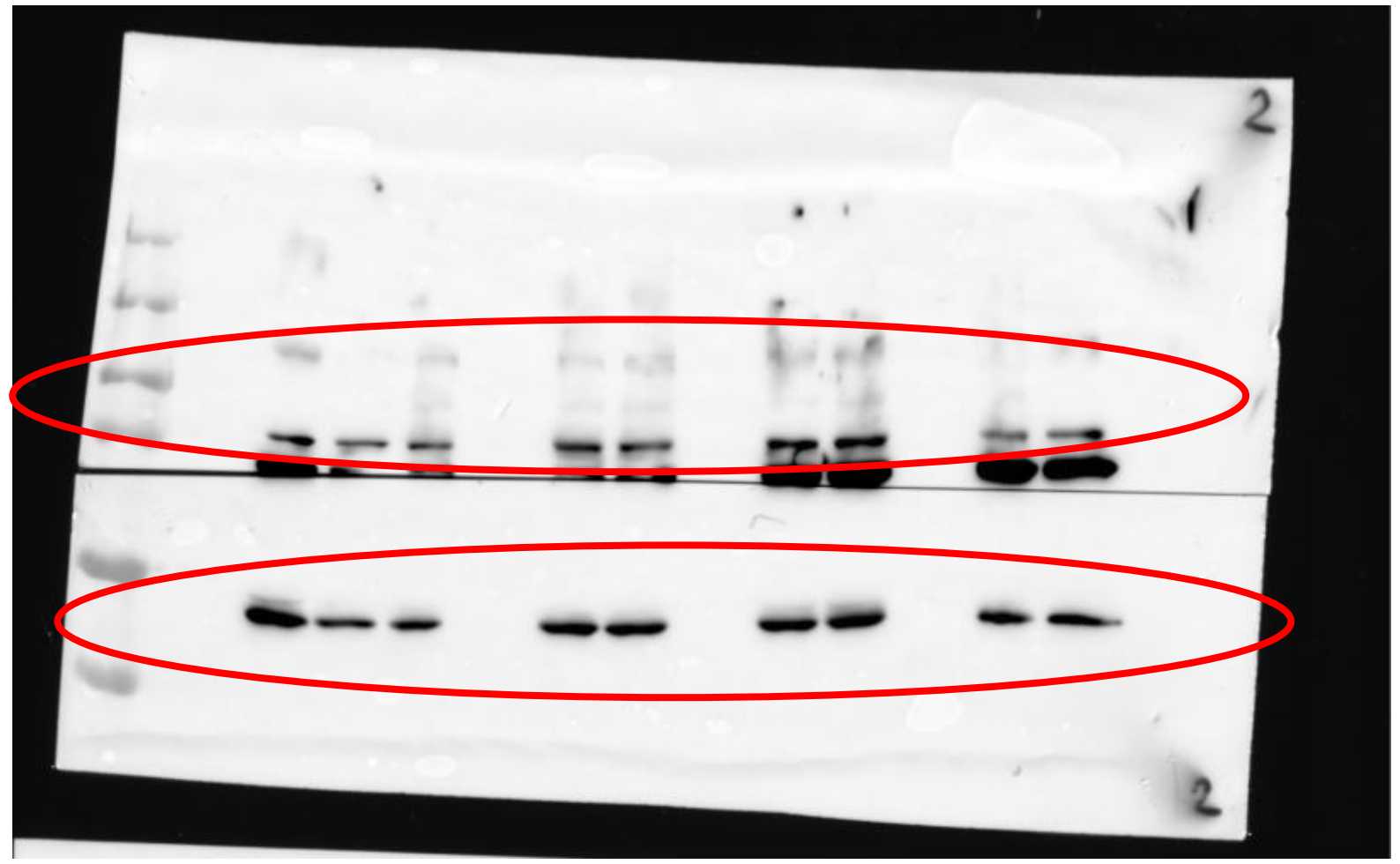

Cropped immunoblot conditions annotated S8I

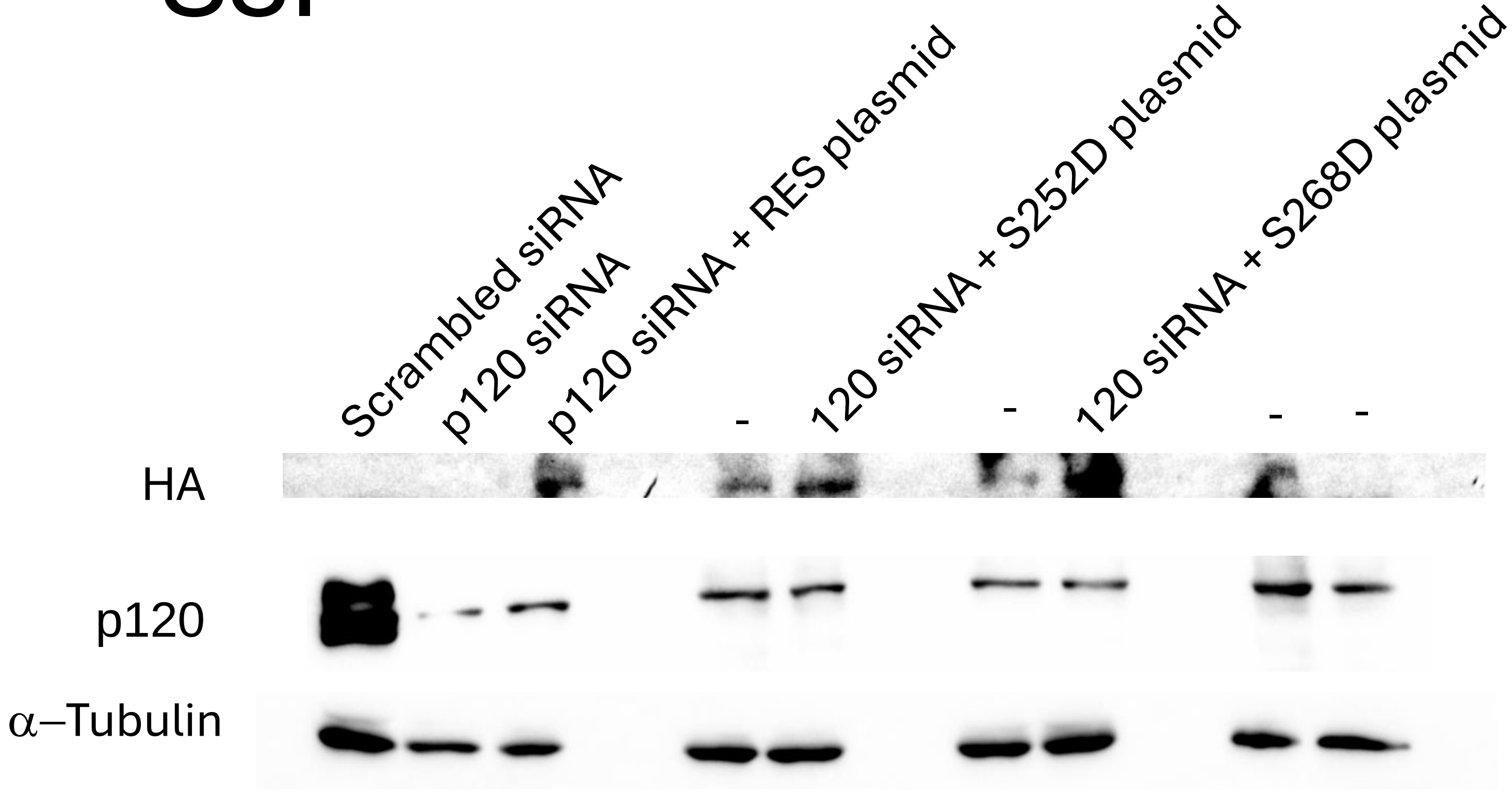

Supplement: Supplementary file 2 [file jciinsight-11-197838-s066.pdf]
